# Chromosomal Copy Number Variation, Selection and Uneven Rates of Recombination Reveal Cryptic Genome Diversity Linked to Pathogenicity

**DOI:** 10.1371/journal.pgen.1003703

**Published:** 2013-08-15

**Authors:** Rhys A. Farrer, Daniel A. Henk, Trenton W. J. Garner, Francois Balloux, Douglas C. Woodhams, Matthew C. Fisher

**Affiliations:** 1 The Department of Infectious Disease Epidemiology, Imperial College London, London, United Kingdom; 2 Institute of Zoology, Zoological Society of London, London, United Kingdom; 3 Department of Genetics, Evolution and Environment, University College London, London, United Kingdom; 4 Department of Ecology & Evolutionary Biology, University of Colorado, Boulder, Colorado, United States of America; Duke University Medical Center, United States of America

## Abstract

Pathogenic fungi constitute a growing threat to both plant and animal species on a global scale. Despite a clonal mode of reproduction dominating the population genetic structure of many fungi, putatively asexual species are known to adapt rapidly when confronted by efforts to control their growth and transmission. However, the mechanisms by which adaptive diversity is generated across a clonal background are often poorly understood. We sequenced a global panel of the emergent amphibian pathogen, *Batrachochytrium dendrobatidis* (*Bd*), to high depth and characterized rapidly changing features of its genome that we believe hold the key to the worldwide success of this organism. Our analyses show three processes that contribute to the generation of *de novo* diversity. Firstly, we show that the majority of wild isolates manifest chromosomal copy number variation that changes over short timescales. Secondly, we show that cryptic recombination occurs within all lineages of *Bd,* leading to large regions of the genome being in linkage equilibrium, and is preferentially associated with classes of genes of known importance for virulence in other pathosystems. Finally, we show that these classes of genes are under directional selection, and that this has predominantly targeted the Global Panzootic Lineage (*Bd*GPL). Our analyses show that *Bd* manifests an unusually dynamic genome that may have been shaped by its association with the amphibian host. The rates of variation that we document likely explain the high levels of phenotypic variability that have been reported for *Bd*, and suggests that the dynamic genome of this pathogen has contributed to its success across multiple biomes and host-species.

## Author Summary

Pathogenic fungi constitute a growing threat to both plant and animal species on a global scale. However, many features of the fungal genome that enable them to successfully adapt to infect diverse hosts and ecological niches remain cryptic, especially for newly evolved emerging lineages. In this paper, we report three novel features of genome diversity linked to pathogenicity in the emerging amphibian pathogen, *Batrachochytrium dendrobatidis* (*Bd*). Firstly, we identified widespread chromosome copy number variation (CCNV) across our lineages, with individual isolates harboring between 2 to 5 copies of each chromosome and rapid rates of CCNV occurring in culture. In addition, by using *in vitro* divergence of replicate lines of *Bd*, we showed that changes in ploidy can occur within as few as 40 generations. Secondly, we identified uneven rates of recombination across the genomes and lineages, revealing hot spots in known classes of virulence factors. Finally we identified significant evidence of diversifying selection across the secretome of *Bd*, and showed that selection also targets putative virulence factors. These findings add to our knowledge of genome-dynamicity and modes of evolution manifested by eukaryote microbial pathogens, and may explain the varied phenotypic responses observed in *Bd*.

## Introduction

A diverse cadre of fungi and fungal-like oomycetes have recently taken centre stage as emerging infectious diseases (EIDs) owing to their increasing impact on animals, plants and wider ecosystem health [1]. The widespread emergence of this class of pathogens shows that they are able to successfully adapt to infect diverse hosts and ecological niches, suggesting that their genomes are able to respond rapidly to natural selection [1], [2]. This idea finds widespread support; for example, horizontal transfer of whole chromosomes [3] and accelerated evolution across functional domains in effector genes [4] are associated with rapid host-adaptation and changes in virulence across lineages and species. Maintaining the pool of genetic diversity necessary to respond to selection is facilitated by the ability of fungi to utilise multiple reproductive modes, including cryptic recombination that enables inbreeding, outcrossing, hybridization, and the generation of diversity *via* parasexual mechanisms [5]. These features are suspected to have contributed to the rise of contemporary fungal EIDs, which play a major role in host population declines across a broad swathe of plant and animal species [1], [6], [7].

In recent years, whole genome sequencing has led to the characterization of novel mechanisms driving dynamic genome structure in microbial eukaryotes. In particular, it is increasingly apparent that pathogenic fungi manifest highly plastic genome architecture in the form of variable numbers of individual chromosomes, known as chromosomal copy-number variation (CCNV) or aneuploidy. This feature has been identified across the fungal phylum Ascomycota, ranging from *Botrytis cinerea* [8], *Histoplasma capsulatum* [9], *Saccharomyces cerevisiae* [10], *Candida albicans* [11] and the Basidiomycota *Cryptococcus neoformans* [12], [13], [14]. The mechanism(s) generating chromosomal CCNV in fungi are not yet well understood, but are thought to occur as a consequence of nondisjunction following meiotic or mitotic segregation [15], followed by selection operating to stabilise the chromosomal aneuploidies [13]. Although stress occurring as a consequence of either host response or exposure to antifungal drugs has been linked to a rapid rate of CCNV in *Candida* [16], it is currently unclear to what extent this contributes to broader rates of CCNV in fungi. However, dynamic numbers of chromosomes could offer routes to potentially advantageous phenotypic changes *via* several mechanisms such as over expression of virulence-factors [13] or drug efflux pumps [17], the maintenance of diversity through homologous recombination [18], increased rates of mutation and larger effective population sizes [19], or by purging deleterious mutations through non-disjunction during chromosomal segregation [20]. Thus, CCNV likely represents an important, yet uncharacterized, source of *de novo* variation and adaptive potential in many fungi and other non-model eukaryote microbial pathogens.

A contemporary EID that is gaining substantial notoriety is the aquatic chytrid fungus *Batrachochytrium dendrobatidis* (*Bd*), which has so far been identified in over 50 countries worldwide and infecting over 500 species of amphibians [21] (http://www.bd-maps.net). One of the most enigmatic aspects of *Bd*'s population genetic structure has been the low levels of genetic variation identified between globally distributed isolates. However, recent studies have shown the existence of up to five separate lineages [22], [23], [24], one of which is shown to have undergone a worldwide range expansion in the 20th Century. We recently compared the genomic diversity of this ‘Global Panzootic Lineage’ (*Bd*GPL) against that of a separate, distantly related (∼1,000 ybp) lineage that appears to have originated in South Africa (named *Bd*CAPE), using SOLiD sequencing. *Bd*GPL was found to harbour evidence of historical recombination, manifested as patchily distributed heterozygosity, and phylogenetic incongruency across small spatial scales that we hypothesised has resulted from ongoing recombination [22]. Therefore, despite the lack of any known sexual meiotic mechanisms in its life cycle, *Bd* clearly has a more dynamic genome than a purely clonal, mitotic mode of reproduction would suggest. Here, we describe a new global panel of isolates that were subjected to high-depth Illumina sequencing in order to better understand cryptic genomic features that are associated with the rapid ascendancy of this pathogen.

## Results

We sequenced 22 isolates of *Bd* with a geographical distribution spanning five continents to a high depth (52–195X; Table 1) using the Illumina HiSeq 2000 platform. Sequences are deposited in the NCBI Short Read Archive under the submission accession number SRA058657. These reads were then aligned to a reference sequence assembly for isolate JEL423 [25] using BWA [26] and polymorphisms were identified using BiSCaP [27] (Text S1: Optimization of alignments and SNP calling parameters). In total, we identified 904,000 SNPs, 761,000 bi-allelic heterozygous positions and 95,000 multi-allelic heterozygous positions (Table S1), which were distributed across 425,000 loci. Of those sites, 8,457 were homozygous across all of the 42 sequenced isolates (Illumina dataset presented here, SOLiD dataset presented previously [22]), which when combined resulted in concordant phylogenetic trees (Fig. S3). These phylogenies showed that our new panel of isolates belonged to three (*Bd*GPL, *Bd*CAPE, *Bd*CH) of the five suspected lineages of *Bd* [22], [23], [24] (Figs. S1, S2, S3) and extended both *Bd*GPL and *Bd*CAPE's known geographic range (*Bd*GPL into Switzerland and Ethiopia and *Bd*CAPE into France). Across the *Bd* JEL423 genome, 96% was covered by at least four reads in every isolate. Additionally, 65% of the total identified variant sites (275,000; 11.8 Kb^1^) were called as either reference or polymorphic in all 22 isolates amounting to >10X the number of ‘covered in all’ polymorphic loci previously found using the ABI SOLiD 3 platform [22], owing to the higher depth of sequencing coverage (Table 1).

**Table 1:** Samples used and details of alignments.

Collection site	Amphibian host	Year	Collector	Culture reference	Passage number	Sequenced depth (X)	Aligned depth (X)
Canada, Vancouver Island	*L. catesbeianus*	2009	PH	VC1 (CA)	>3	52.75	49.17
England, Cumbria	*E. calamita*	2010	PM	SFBC014 (GB)	2	115.32	106.96
Ethiopia, Hotcho	*A. enseticola*	2011	DG	ETH2 (ET)	2	68.90	58.66
Ethiopia, Telilia Stream near Rira	*Leptopelis sp.*	2011	DG	ETH4 (ET)	2	166.03	152.87
France, Lac d'Aule	*A. obstetricans*	2010	MF	AUL (FR)	2	195.89	175.48
France, Loire et Cher	*L. catesbeianus*	2010	CM	RC5.1 (FR)	3	85.28	67.65
France, Madamette	*A. obstetricans*	2010	MF	MAD (FR)	2	127.41	110.29
Mallorca, Torrent des Ferrerets	*A. muletensis*	2007	MF	TF5a1 (ES)	>3	150.33	133.33
Panama, Guabal	*P. lemur*	2004	JEL	JEL423 (PA)	>3	53.32	48.48
Sardinia, Affluente Pisharoni	*D. sardus*	2010	TG	AP15 (IT)	2	179.93	164.91
Sardinia, Monte Olia	*D. sardus*	2010	TG	MODS27 (IT)	2	52.84	49.17
Sardinia, Monte Olia	*D. sardus*	2010	TG	MODS28 (IT)	2	160.15	148.47
Sardinia, Scuponi	*D. sardus*	2010	TG	SP10 (IT)	2	129.60	115.46
South Africa, Mount, KZN	*A. vertebralis*	2010	TG	MG1 (ZA)	2	81.71	61.64
South Africa, Pinetown Kwazulu	*A. angolensis*	2011	TG	SA1d (ZA)	2/3	148.72	136.69
South Africa, Pinetown Kwazulu	*A. angolensis*	2010	TG	SA4c (ZA)	2	180.52	161.82
South Africa, SilverMine, KZN	*A. fuscigula*	2010	TG	MG4 (ZA)	2	131.16	122.30
Switzerland, Gamlikon	*A. obstetricans*	2007	TG	ACON (CH)	>3	167.19	144.62
Switzerland, Gamlikon	*A. obstetricans*	2008	TG	APEP (CH)	>43	110.43	100.99
Switzerland, Gamlikon	*A. obstetricans*	2007	TG	CON2A (CH)	>43	115.29	102.17
Switzerland, Itingen	*A. obstetricans*	2010	RF	BLI1 (CH)	2	52.76	49.32
Switzerland, Waltisberg	*A. obstetricans*	2010	RF	BEW2 (CH)	2	144.54	132.07

*Bd* isolates and locations that were resequenced. The first 4 columns provide information for the recommended naming scheme outlined by Berger *et al.* [47]. Passage numbers are best approximations from records prior to DNA extractions in January and May 2011. The sequenced depth and aligned depth were calculated from the number of nucleotides in all or aligned reads respectively and divided by 24 Mb (the length of the *Bd* JEL423 genome assembly). All isolates represent novel sequences, apart from JEL423 and TF5a1 [22]. Amphibian hosts include *Afrixalus enseticola* (Ethiopian Banana frog), *Alytes muletensis* (Mallorcan Midwife Toad), *Alytes obstetricans* (Common Midwife Toad), *Amietia angolensis* (Angola River Frog), *Amietia fuscigula* (Cape River Frog), *Amietia vertebralis* (Ice Frog), *Discoglossus sardus* (Tyrrhenian Painted Frog), *Epidalea calamita* (Natterjack Toad), *Leptopelis sp.* (Big eyed Tree Frog), *Lithobates catesbeianus* (American Bullfrog), *Phyllomedusa lemur* (Lemur Leaf Frog). CM = Claude Miaud, DG = David Gower, JEL = Joyce Longcore, MF = Matthew Fisher, PH = Phineas Hamilton, PM = Peter Minting, RF = Rhys Farrer, TG = Trent Garner.

### Chromosomal Copy Number Variation (CCNV)

**Figure 1:**
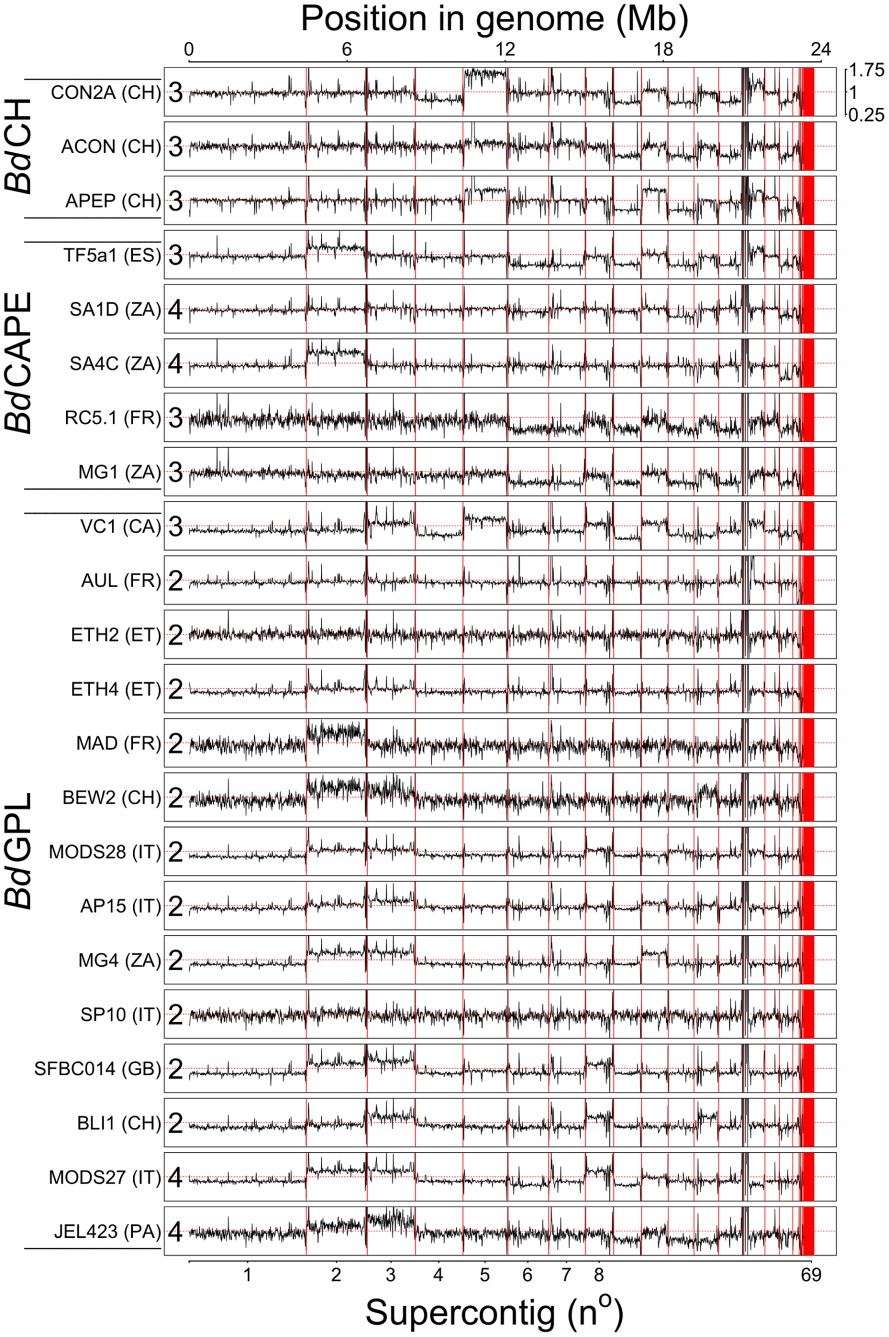
Read depth across 22 genomes was normalised by total alignment depth and plotted against location in the genome using a 10 Kb long non-overlapping sliding window. Base ploidy levels were determined using allele frequencies for supercontig 1 and shown at the start of each plot. Intra-chromosome read depth is largely consistent amongst the isolates, except over supercontig 14 due to a long stretch of rDNA. Shifts in read-depth between chromosomes demonstrate variation in chromosome copy number.

Comparing the depth of read coverage over each chromosome using 10 Kb non-overlapping sliding windows revealed CCNV present in isolates belonging to all three lineages of *Bd* and affecting nine of the largest fifteen supercontigs (Figs. 1 and S4). *t*-tests on the mean depths across windows compared with those in the largest supercontig confirmed a significant increase in read-depth across 36 supercontigs, and a significant decrease in depth across 25 supercontigs in 18 of the 22 sequenced isolates (Fig. S5). To further verify relative ploidy within an isolate and the order of ploidy-changes, we inferred whether individual bases were ‘evenly’- or ‘oddly’-distributed across Illumina reads within a single genome by binning their frequencies into histograms for each chromosome. The expectation here is that a chromosome with an even ploidy will tend towards a 50∶50 distribution across each single SNP, while chromosomes with an odd ploidy will tend towards a 33∶66 or 33∶33∶33 ratio across SNP-calls (Figs. S5, S6). This method identified even- or odd-ploidies for 92% of the chromosomes tested with >95% bootstrap support (Table S2).

Thirteen *Bd*GPL and two *Bd*CAPE isolates had greater numbers of bi-alleles than tri-alleles (corresponding to an even ploidy that most parsimoniously corresponds to diploidy) (Table S2), and six isolates belonging to all three separate lineages that had greater numbers of tri-alleles than bi-alleles (corresponding to an odd ploidy that most parsimoniously corresponds to triploidy). The remaining four isolates (*Bd*GPL JEL423 & MODS27, *Bd*CAPE SA1d & SA4c) had significant *p*-values showing between 1–3 chromosomes in lower ploidy levels relative to the remaining bi-allelic genome. Over these lower-ploidy chromosomes we observed greater numbers of tri-alleles than bi-alleles and no decrease in heterozygous base-calls (both of which should occur if these chromosomes were haploid). We therefore conclude that these four isolates have tetraploid genomes with the identified losses in read-depth corresponding to chromosomes that have lost a single copy and are now trisomic.

**Figure 2:**
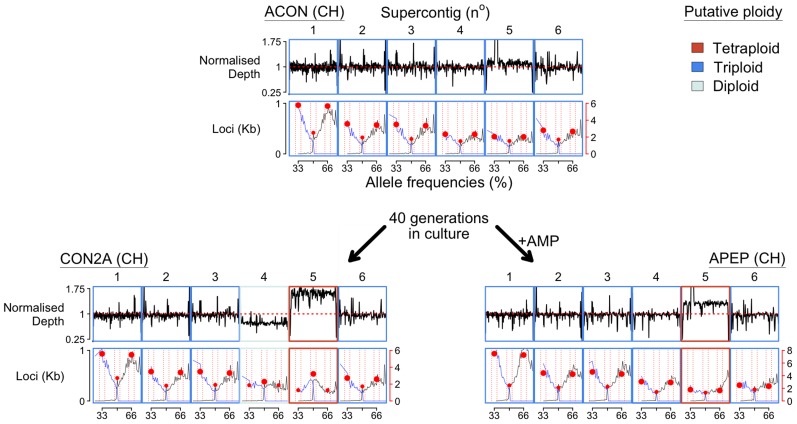
Chromosome copy number variation was identified across the three *Bd*CH isolates (ACON and its progenitors CON2A and APEP) following 40 generations in culture with or without the addition of anti-microbial peptides (AMP), respectively. Read depth is normalised to total alignment depth. A tally of all loci (per kilobase) with between 25–75% reads agreeing with the reference nucleotide are shown below, and summarised by the most common allele (black line), the second most common allele (blue line), and bins between 32–34, 49–51 and 65–67% (red circles). ACON is putatively triploid across the largest six supercontigs, whereas CON2A has lost a copy of supercontig IV and gained a copy of supercontigs V. APEP has gained a copy of supercontigs V.

We were able to take advantage of replicate lines of *Bd*CH, which were passaged for 40 generations with and without exposure to skin antimicrobial peptides collected from the water frog *Pelophylax esculentus*. In these culture lines, the ancestral putatively triploid isolate (*Bd*CH ACON) differentially lost and gained copies of supercontig IV and V respectively when passaged without selection (*Bd*CH CON2A), and gained a copy of supercontig V following treatment with antimicrobial peptide (*Bd*CH APEP), which resulted in a significant reduction in mean growth inhibition (Text S1: *In vitro* Divergence of Independent Replicate Lines of *Bd*CH; Fig. 2). Due to the fact that most of our isolates exhibiting CCNV were sequenced shortly following isolation from nature without sequential passage, we know that CCNV is occurring frequently in both wild and cultured isolates. The rapidity that these mutations are accumulating across our isolates shows that aneuploidies in *Bd* are occurring at rates that will generate genome diversity within the timescale of a single host infection.

### Recombination

In order to detect the presence and frequency of recombination events we determined the phase of bi-allelic heterozygous polymorphisms (Table S1, Figs. S7, S8, S9). We focused our attention on SNPs that were supported by a high percent of uniquely mapped reads (Table S1) and reads agreeing with the phasing (Fig. S9). By performing pairwise comparisons of shared phased positions between each of our isolates, we found >99% of these sites remained in the same phase for intra-lineage comparisons and >92% for inter-lineage comparisons (Fig. S10). However, we also identified 4,974 haplotypes demonstrating crossovers (Fig. S11) where all four pairwise combinations of bases were observed. Of these, 2,007 occurred at unique positions/loci in the genome. Every pair of isolates that we compared (except between *Bd*GPL isolates MAD (FR) and AUL (FR)) showed at least one haplotype that included an inferred crossover (Fig. S11). This was surprising given many of the isolates share a very recent common ancestor. For instance, we found that two isolates (MODS27 and MODS28) which were recovered from *Discoglossus sardus* at a single site in Sardinia on a single collection trip and are closely related (Fig. S2) had accumulated three crossovers. This shows that recombinant genotypes can accumulate even within highly-related free-living populations of *Bd*, a feature of this chytrid's population genetics that was first remarked upon by Morgan *et al.* in populations of Sierra Nevadan *Rana muscosa* [28].

**Figure 3:**
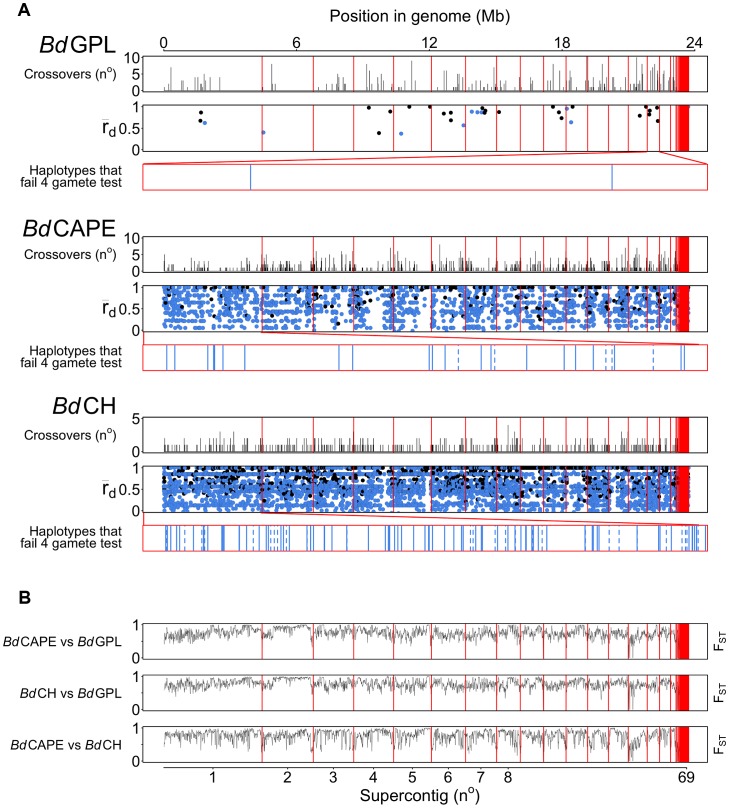
Crossovers were detected with pairwise comparisons for each *Bd* isolate across every supercontig. (A) Crossovers detected between isolates from each of the lineages were tallied and plotted across 10 Kb windows. rBarD was calculated for all haplotypes taken across phased regions of the genome. Haplotypes in linkage equilibrium are shown in blue and those in disequilibrium are shown in black. The supercontigs with the greatest number of haplotypes in linkage equilibrium are shown below rBarD values in red boxes. Haplotypes over genes are shown as a solid black line and haplotypes over intergenic regions are shown with a dotted line. (B) Fixation Indices (F_ST_) were calculated between each of the lineages using 10 Kb windows revealing no strong evidence for introgression between each of the three lineages sequenced.

The greatest proportion of phased positions demonstrating crossovers were found to occur between the three lineages, demonstrating an accumulation of recombinant haplotypes that scales with time of divergence (Fig. S11). For example, as many as 7.3% of the phased positions revealed crossovers that have accumulated since isolates *Bd*GPL AP15 (IT) and *Bd*CH ACON (CH) were separated. Crossovers were identified in every major chromosome, and predominantly identified in intergenic regions (143 Mb^−1^ compared with 57 Mb^−1^ for coding regions and 65 Mb^−1^ for introns) (Figs. 3A, S12, S13).

Crossovers were also found to occur with a higher frequency amongst isolates belonging to the lineages *Bd*CAPE and *Bd*CH (between 0.6 and 1.1% of phased positions, respectively) compared with 0–0.2% in *Bd*GPL. This was a surprising finding given that the three *Bd*CH isolates were separated by only 40 passages in the lab and were derived from a single isolate that had been relatively recently isolated in 2007. This suggests two hypotheses: Either *in vitro* passage under selective conditions promotes rapid recombination, or our isolate of *Bd*CH is descended from a population of *Bd* that is more recombinogenic than *Bd*GPL. To further study the amount of recombination within lineages and between isolates, we extracted haplotypes that were phased across all of the isolates within a given lineage and contained at least two alleles per loci (ranging in length from 11 nt to 33.3 Kb: Fig. S12). Because only 35 haplotypes were retained for the entire panel of *Bd*GPL isolates, we also extracted haplotypes from two *Bd*GPL subsets consisting of 3 and 5 isolates respectively, thus allowing higher numbers of crossovers to be retained. From each of these sets of haplotypes, we calculated a multilocus measure of linkage disequilibrium (the standardised index of association rBarD [29]) and applied Hudson's four-gamete test [30] in order to quantify the amount of recombination amongst isolates within each lineage (Table 2). Across the *Bd*GPL groups, >30% of phased positions were in significant disequilibrium compared with 16% and 11% for *Bd*CH and *Bd*CAPE respectively. RbarD appeared to be robust against sample size differences, and gave values from *Bd*GPL values of 0.79–0.82 compared against 0.58 and 0.61 for *Bd*CH and *Bd*CAPE. Finally, a smaller proportion of *Bd*GPL subset haplotypes failed the four-gamete test compared with *Bd*CAPE or *Bd*CH isolates. Each of these findings shows that recombination is causing diversity within each of the lineages. However, the emergent *Bd*GPL is far more clonal than either of the other two lineages.

**Table 2:** Haplotypes from isolates belonging to each of the separate lineages were tested for linkage disequilibrium using the index of association (I_A_), rBarD and the 4-gamete test.

Lineage	Isolates	Haplotypes	Length (nucleotides)	Loci	Significant Disequilibrium (%)	Mean rBarD	Fail 4-gamete test (%)	Variable sites per locus
*Bd*GPL	14	35	4,409	95	68.57	0.82	7 (20%)	2 nt = 54 (56.84%)3 nt = 28 (29.47%)4 nt = 13 (13.68%)
*Bd*GPL subset 1	3	919	341,325	2,822	31.12	0.79	61 (6.64%)	2 nt = 2,118 (75.05%)3 nt = 575 (20.38%)4 nt = 129 (4.57%)
*Bd*GPL subset 2	5	438	83,414	1,232	41.32	0.82	36 (8.22%)	2 nt = 861 (69.89%)3 nt = 301 (24.43%)4 nt = 70 (5.68%)
*Bd*CAPE	5	2,275	952,307	7,212	11.47	0.61	197 (8.66%)	2 nt = 5,377 (74.55%)3 nt = 1,709 (23.70%)4 nt = 126 (1.75%)
*Bd*CH	3	5,215	1,537,742	16,612	16.36	0.58	655 (12.56%)	2 nt = 11,920 (71.76%)3 nt = 4,184 (25.19%)4 nt = 508 (3.06%)

To check differences between lineages were not resulting from different numbers of isolates, 2 subsets were made from *Bd*GPL. Subset 1 consisted of isolates VC1, AP15 and JEL423. Subset 2 consisted of subset 1, ETH4 and MODS27. For each isolate subset, the total length (in nucleotides) of all haplotypes and the total number of loci with ≥2 alleles is given. Over 30% of the *Bd*GPL haplotypes from any of the subsets were in significant disequilibrium, whilst only 11% of the haplotypes in *Bd*CH and 16% of the haplotypes in *Bd*CAPE were in disequilibrium, suggesting these populations are recombining more than the clonal *Bd*GPL. The numbers of variable sites per locus are also shown, demonstrating all lineages to be as likely to have arisen from out-crossing.

**Figure 4:**
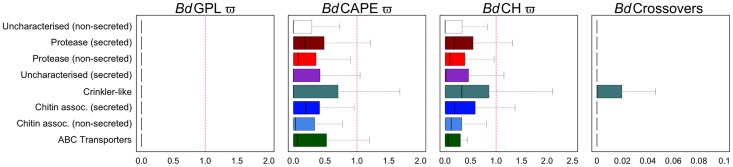
Boxplots for eight non-overlapping gene categories comprising every gene were compared for ratios of non-synonymous to synonymous mutations for each of the three lineages (dN/dS) and numbers of crossovers per phased positions (PP) within each gene (≥2PP) for all isolates (outliers omitted for both). Proteases and chitin-associated genes with predicted signal peptides had greater *dN*/*dS* ratios than those without for both *Bd*CAPE and *Bd*CH. CRN-like genes had the greatest upper quartile and upper tail showing these to be the most variable genes in the genome.

We next investigated whether recombination had occurred between these three lineages since their divergence, by calculating θ, Weir's [31] formulation of Wright's fixation index (*F*_ST_) for pairwise comparisons of each lineage across window lengths of 1.4 Kb and 10 Kb (Figs. S14 and 3B). We found that all three lineages were highly differentiated from one another across each chromosome, with only minor intra-chromosomal regions of high similarity (which mainly comprised a long stretch of rDNA located at the start of chromosome 14). This indicates that recombination amongst these lineages has not occurred since their separation. We then determined whether certain categories of genes were associated with higher-than-average rates of recombination using *t*-tests on numbers of crossovers after accounting for differing levels of heterozygosity and density of phased-sequences (Text S1: Identifying gene groups and names; Table S3, Figs. 4 and S15, S16). Surprisingly, we found only one group showing significant enrichment for crossovers: those showing homology to the C-terminal of the Crinkler (CRN) family of oomycete effector proteins found in the *Phytophthora* genus [32], [33]. Enrichment was found in both *Bd*GPL and *Bd*CAPE, whilst not in *Bd*CH. Haplotypes that failed the four-gamete test were predominantly from coding-regions, but had no clear pattern of enrichment for any gene category (Table S4).

### Patterns of mutation and selection

To identify genes that are present in the reference sequence and absent in our panel of isolates (presence/absence polymorphism), we examined the read-depth across each of the genes. Only five genes were identified from our panel (Table S5), including three amongst *Bd*CAPE isolates and two amongst *Bd*CH isolates. Therefore, whilst high-levels of aneuploidy are occurring, it does not appear to be resulting in frequent gene loss. To study the patterns of mutation across the nuclear genome, we categorized each of the mutations by their location in the genome in terms of coding regions (CDS), introns and intergenic regions (Table S6). In every isolate we sequenced, every variant type was found in greater abundance per kilobase in the non-coding regions (with the exception of 0.01 Kb^−1^ fewer heterozygous positions in the introns compared with the CDS for isolate MG1). This overall pattern can be explained through selection purging deleterious mutations from the CDS. In addition, we found homozygous polymorphisms to be highly supported in all lineages in terms of uniquely mapped reads, whilst un-phased bi-allelic heterozygous positions had a smaller total proportion in the divergent lineages compared with *Bd*GPL, suggesting some heterozygous positions may be miscalled due to paralogs.

We categorised each of the mutations within the CDS into synonymous and non-synonymous mutations (Table S6). SNPs were responsible for 169,000 synonymous changes and 197,000 non-synonymous changes. Genes with putative roles in pathogenicity were grouped by searching for secretion signals, protease domains and carbohydrate binding domains (Text S1: Identifying gene groups and names), and tested each of these for enrichment of homozygous SNPs (Tables S7 and S8) and heterozygous positions (Table S9) using hypergeometric tests. We found that gene groups that carried a secretion signal (proteases, chitin-binding and uncharacterized secreted) as well as CRN-like genes, were significantly enriched for both homozygous and heterozygous polymorphisms relative to the whole set of genes. Predicted chitin-binding proteins that lacked a secretion peptide were not enriched for either homozygous SNPs or heterozygous positions (Tables S7 and S9), and non-secreted proteases were only enriched for synonymous amino acid changes. Conversely, CRN-like genes are only enriched for non-synonymous homozygous SNPs and not synonymous SNPs.

We next measured the rates of synonymous substitution (*dS*), non-synonymous substitution (*dN*) and omega (*dN*/*dS* = ω) for every gene in every isolate and compared values by grouping isolates into their lineages (Fig. 4, Table S10). In total, we identified 1,450 genes with ω≥1 in at least one of our isolates (*Bd*CAPE = 816; *Bd*CH = 746; *Bd*GPL = 283), suggesting positive or diversifying selection. Although no clear pattern could be distinguished within *Bd*GPL (Fig. S17) owing to the high degree of relatedness amongst isolates and thus relative paucity of polymorphism, CRN-like genes in both *Bd*CAPE and *Bd*CH had the greatest median, upper quartile and upper tail values of omega (Fig. S18). In addition, average ω values for secreted chitin-associated genes and proteases were marginally higher than their non-secreted counter parts. Uncharacterized secreted genes also had a greater ω than either of those non-secreted gene groups. Finally, a significant enrichment of both CRN-like genes and uncharacterized (secreted) genes with ω≥1 were identified in both *Bd*CAPE and *Bd*CH (Table S11).

By analysing each of these 1,450 genes with ω≥1using branch site models (BSM) in PAML along each of the three lineages of *Bd*, we identified a subset of 482 genes that show evidence for positive selection in at least one of the lineages. For *Bd*CAPE and *Bd*CH, a greater percent of each of the secreted gene categories were found to have accumulated an excess of non-synonymous mutations compared with their non-secreted counterpart gene categories (Table S11). Nine genes were also identified in all three lineages (Fig. S19), including four uncharacterized secreted and five uncharacterised non-secreted genes. However, the most striking finding of this analysis was found among *Bd*GPL isolates where 349/482 (72%) of the genes showed a signature of positive selection compared with only 23% for each of the other two lineages. This finding suggests that *Bd*GPL has been undergoing greater levels of positive selection than either *Bd*CAPE or *Bd*CH, despite the low numbers of sites under selection owing to the high levels of relatedness within this lineage.

## Discussion

Recent studies have attributed aspects of *Bd*'s pathogenesis to the presence of a number of putative virulence factors that include proteases and chitin-binding proteins [32], [33], [34]. The former category contain M36 or S41 domains that are thought to degrade host-cellular components, and these protease families are known to have undergone extensive expansions in *Bd* since its divergence from free-living saprobes such as *Homolaphlyctis polyrhiza* [32]. Chitin binding proteins are thought to be involved in pathogenesis by allowing *Bd* to bind to keratinized host cells and to subsequently enter the host cells [34]. To date, the functional nature of the crinkler-like family in *Bd* has only been inferred owing to their homology to host-translocated proteins of known virulence in oomycetes [33]. Our data show that, across this global panel of 22 isolates and three lineages, the secretome and crinkler-like family of genes manifest higher diversity of homozygous and heterozygous SNPs, enrichment for non-synonymous mutations and greater *dN*/*dS* (ϖ) ratios when compared against classes of genes that do not contain a signal peptide. This shows that these gene families are evolving most rapidly in *Bd*, and that gene-products that interact with the amphibian host are undergoing diversifying (or reduced purifying) selection when compared with those gene-products that remain intracellular. Our findings suggest that *Bd* has had an evolutionary association with amphibians that predates the radiation of the lineages that we have characterised here, and is further evidence that this chytrid has an obligate rather than an opportunistic association with its amphibian hosts.

By mapping read-depth and SNPs across these genomes, we discovered that widespread genomic variation occurs within and amongst *Bd* isolates from the level of SNPs up to heterogeneity in ploidy amongst genomes and amongst chromosomes within a single genome. Individuals from all three lineages harboured CCNV along with predominantly or even entirely diploid, triploid and tetraploid genomes. Recent research by Rosenblum *et al.* [35] has also identified widespread CCNV across diverse lineages of *Bd* recovered largely from infected amphibians in the Americas, including a single haploid chromosome in isolate *Bd*GPL JEL289. This variation may itself, reflect only part of the full diversity in *Bd*,pathogensas +2/+3 shifts in ploidy, whole genomes in tetraploid, or chromosomes in pentaploid or greater, may occur and await discovery. Chromosomal genotype was shown to be highly plastic as significant changes in CCNV occurred in as few as 40 generations in culture. It is not known whether other chytrid species also undergo CCNV, or if this is a unique feature of *Bd* and hence may be intrinsic to its parasitic mode of life. Currently, CCNV is known to occur in a variety of protist microbial pathogens, including fungi, however it is currently not known whether this genomic-feature is specific to a parasitic life-style, or is a more general feature of eukaryote microbes; identifying the ubiquity of CCNV or otherwise across nonpathogenic species will therefore be of great interest. Further, the manner in which the plasticity of CCNV in *Bd* affects patterns of global transcription and hence the phenotype of each isolate also remains to be studied. However, it is clear from research on yeast, *Candida* and *Cryptococcus*, that CCNV significantly contributes to generating altered transcriptomic profiles, phenotypic diversity and rates of adaptive evolution even in the face of quantifiable costs; understanding the relationship between CCNV and *Bd*-phenotype will therefore likely be key to understanding its patterns of evolution at both micro- and macro-scales.

Whilst differing numbers of individual chromosomes presents a potential barrier to the standard model of meiosis, homologous recombination may still be occurring *via* mitotic processes within compatible genomes. In order to study recombination amongst our isolates, we determined the phase of our reads and constructed haplotypes that were suitable for traditional population genetic tests. This showed that, whilst the majority of the genomes from all three lineages manifest widespread linkage disequilibrium, recombination could still be detected across each chromosome and in all genomes. Crossovers (measured both as the proportion of SNPs that change phase and the numbers of haplotypes failing Hudson's four-gamete test) were found to occur much more frequently within the *Bd*CAPE and *Bd*CH lineages compared to *Bd*GPL, and these two lineages accordingly manifest lower average linkage disequilibrium. All of the *Bd*CH genomes that we sequenced stem from a single isolate collected in 2007. This suggests that either the high rates seen here have accrued since the isolate was taken into culture (suggesting a very rapid rate of *in vitro* recombination), or that we are characterising recombination events that occurred *prior* to the isolation of *Bd*CH and are segregating as a consequence of the multiple-ploidy nature of *Bd*. In support of the latter hypothesis, comparisons between population-level data for *Bd*GPL and *Bd*CAPE show that *Bd*GPL is far less recombinogenic and has been undergoing a largely clonal expansion since its emergence, consistent with previous observations made by James *et al.* [36]. These data suggests that the global *Bd*GPL population is derived from a less recombinogenic ancestor than either *Bd*CH or *Bd*CAPE, that contemporary recombination is not occurring at a rapid rate and, where it occurs, is the result of a selfing rather than outcrossing events.

The discovery of a lower proportion of variable sites across haplotypes in addition to the lower proportion of heterozygous positions in *Bd*GPL compared against *Bd*CAPE or *Bd*CH does not support the notion that *Bd*GPL is an outbred hybrid lineage as previously proposed [22]. The discovery of a new *Bd* lineage found in Brazil (*Bd*Brazil) along with an isolate that is a likely *Bd*GPL/*Bd*Brazil recombinant [24] strongly implies that *Bd* retains the ability to outcross, despite having a primarily clonal genome and life cycle. However, values of *F*_ST_ across our dataset show no introgression between the three lineages; this demonstrates that they have remained largely separate since their divergence and suggests that outcrossing between lineages of *Bd* is rare or, if it has occurs, remains spatially restricted. Further broad-scale collections of isolates and extension of our comparative-population genomic analyses will allow the assignment of more accurate rates of introgression across evolutionary timescales.

We show that rates of recombination are uneven across the genome, with CRN-like genes enriched for crossovers, suggests that either CRN-like genes might have features that favour recombination or that recombinants of these genes have a fitness advantage and are thus more likely to reach fixation than recombinants at other locations in the genome. CRNs were also enriched for non-synonymous polymorphisms, are characterised by a signal of directional selection, and are amongst the most polymorphic genes in *Bd*'s genome. Within the oomycete genus *Phytophthora*, CRNs manifest diverse carboxy-terminal domains and high rates of homologous recombination targeted to the conserved HVLVXXP motif, suggesting that the mosaic domains of CRNs are being shuffled by recombination [2]. Recently, a number of *Bd* CRNs have been shown to be highly expressed on host tissue *in vitro* [37]. Therefore, whilst these genes in *Bd* lack a secretion signal, their expression, accumulation of genetic variation in terms of recombination and ϖ values, and similarities with oomycete CRNs strongly suggest that a number of these CRN-like genes are functional in *Bd*. However, whether they contribute directly or indirectly to the virulence of *Bd* remains to be determined.

Our demonstration of multiple hierarchies of cryptic genomic variation in *Bd* in terms of CCNV, ubiquitous and potentially targeted recombination, and natural selection, points to an ability to generate diversity without the necessity of an obligate sexual stage. Our study has uncovered high levels of genotypic plasticity that are likely to cause widespread phenotypic plasticity even without the need to invoke outcrossing. These large and small-scale changes are therefore likely to contribute to rapid evolutionary rates in the face of an effective host response. Such ‘genomic instability’ may explain the diverse phenotypic responses observed in *Bd* [38], and may also explain the enormous diversity of hosts and biomes that this generalist pathogen has managed to infect.

## Materials and Methods

Full details are given in Text S1, Supplemental Materials and Methods. Briefly, twenty-two isolates that had been collected from nine countries and four continents were chosen for sequencing (Table 1). Paired-end Libraries were constructed according to the protocols provided by Illumina sequencing (Truseq kit). The genome sequence and feature file for the chytrid fungus *Batrachochytrium dendrobatidis* (*Bd*) strain JEL423 was downloaded from http://www.broadinstitute.org/ (GenBank project accession number AATT00000000). The feature file for JEL423 had all but the longest splice variants removed for each gene leaving 8794/8819 genes. We aligned our reads to the genome sequence using Burrows-Wheeler Aligner (BWA) v0.5.9 [26] with default parameters, converted to Samtools mpileup format using SAMtools v.0.1.18 [39] and polymorphisms called using the Binomial SNP-Caller from Pileup (BiSCaP) v0.11 [27]. For phylogenetic analysis we extracted polymorphisms covered ≥4 reads in all 22 isolates. FASTA files were converted into Nexus files and trees constructed using the Un-weighted Pair Group Method with Arithmetic Mean (UPGMA) algorithm in PAUP and visualised using Figtree [40] (Fig. S3). Gene groups were identified using gene-annotations, blastx searches (1e^−05^ e-value cut-off) to the non-redundant BLAST database, SignalP3.0 [41], Merops [42] and Procarb604 v1 [43].

Chromosome copy number variation (CCNV) was identified using changes in both depth of coverage and percent of reads specifying two most frequent alleles at any locus. To quantify these changes, we first performed *t-tests* (with a cut-off of p<5^−10^) on the mean depths across the largest supercontig (supercontig 1) against each subsequent supercontig for each isolate (Fig. S5). Next, we calculated the percent of reads specifying the two most frequent alleles (Fig. S7) for each chromosome in each isolate separately using a minimum depth of 4 reads for both alleles and binned values falling between 47–53% (expected even ploidy/bi-allelic) and 30–36% and 63–69% (expected odd ploidy/tri-allelic). To account for depth and mutation variation within a chromosome, we performed 1000 bootstraps for either predominance of bi-allelic or tri-allelic peaks (Table S2). Using a 5% cut-off (5%<x<95%) we found 305/330 largest 15 chromosomes gave confident odd or even allelic peaks and was largely concordant with changes predicted by *t*-tests.

To detect recombination, we identified haplotypes using reads that overlapped two or more bi-allelic heterozygous positions. Haplotypes from each isolate were then compared to haplotypes in other isolates. We also calculated the Index of association (I_A_), detecting linkage disequilibrium for a given set of haplotypes if VD>L (L_old_). We also calculated rBarD values and performed 4 gamete tests between every combination of loci in a haplotype (Fig. S9) to quantify the amount of recombination occurring within populations. In addition, we applied Weir's [30] estimator of Wright's Fixation Index (*F*_ST_) according to the equations given in Multilocus 1.3 [29].

For selection, we used the yn00 and codeml programs of PAML [44] implementing the Yang and Nielsen method [45] on every gene in every isolate and those with ω≥1 respectively. For codeml, we used the Branch site model (BSM) A (model = 2, NSsites = 2, fix_omega = 0) compared with the null model (model = 2, NSsites = 2, fix_omega = 1, omega = 1). Next, we calculated 2 * the log likelihood difference between the two compared models (2D′) with two degrees of freedom, and identified any with values greater than 8.1887 and 11.4076 (5% and 1% significance after Bonferroni correction). Enrichment for crossovers and polymorphisms was detected using hypergeometric tests and *t-tests*.

For *in vitro* divergence, an isolate of *B. dendrobatidis* from a Swiss *Alytes obstetricans* (isolate 0739) was subcultured into control (ACON) and peptide-treated (APEP) culture flasks containing 10 ml 1% tryptone media supplemented with 1% penicillin-streptomycin (Sigma) to reduce the risk of bacterial contamination. Cultures were incubated at 18°C and passaged every 4–5 d by scraping the side of the flask and transferring 1 ml into 9 ml fresh media. Peptide-treatment included addition to the media of 80 µg ml-1 skin defense peptides collected from *Pelophylax esculentus* (n = 15 combined) according to Daum *et al.* (2012) [46]. This was equivalent to the IC_50_, or the concentration at which growth of *Bd* was inhibited by 50%.

## Supporting Information

Figure S1 The previous SOLiD reads (5) and the new Illumina paired end reads of *Bd* isolate JEL423 were aligned to a modified JEL423 reference sequence. Additionally, simulated reads from a heterozygous reference sequence were made to the depths of the Illumina and SOLiD datasets. Single Nucleotide Polymorphisms (SNPs) and heterozygous positions were then called and the False Discovery Rates (FDR) ascertained. The SNP-caller BiSCaP v0.11 was tested using default settings, and SAM/BCFTools with VCFUtils was tested for its ability to call SNPs using its default settings. SNPs were also filtered for those found without first modifying the reference sequence (f = filtered). (**A**) 1 nt/Kb simulated SNPs or heterozygous positions (12,458 in total) within the coding region (CDS) (**B**) 1 nt/100 nt simulated SNPs or heterozygous positions (124,588 in total) within the CDS region. The new Illumina data was able to recover >95% of true positive SNPs and >80% true positive heterozygous positions using BiSCaP v0.11, outperforming the previous lower-depth SOLiD sequences.
(PNG)

Figure S2 The percent of ECVA polymorphic sites shared between each of the 22 isolates. Greater overlap (≥30%) highlighted in red. (**A**) The overlap of homozygous SNPs varied between 3% and 97% (**B**) The overlap of heterozygous positions varied between 3% and 75%.
(PNG)

Figure S3 Phylogenetic trees were made using the UPGMA algorithm in PAUP from ECVA polymorphic positions identified in the nuclear genomes demonstrating three divergent lineages (*Bd*GPL, *Bd*CAPE and *Bd*CH shown in red blue and green respectively). (**A**) A tree from 275 Kb ECVA polymorphic positions identified from Illumina sequencing. (**B**) A tree from 36 Kb ECVA polymorphic positions from Illumina and SOLiD sequencing. (**C**) A tree from 218 Kb EVCA homozygous positions identified from Illumina sequencing. (**D**) A tree from 8 Kb EVCA homozygous positions identified from Illumina and SOLiD sequencing.
(PNG)

Figure S4 CCNV in the *Bd* nuclear genomes was identified using allele-frequencies and mean read depths across each chromosome normalised to the alignment depth for each isolate. Many *Bd*GPL isolates can be seen to include more copies of chromosome 2 and 3, while the 3 *Bd*CH and 3 of the 5 *Bd*CAPE isolates have fewer copies of chromosome 9 and 11. Fewer copies of chromosome 9, 11 and 16 appear to be found in many of the isolates.
(PNG)

Figure S5 *t*-tests for the mean depth of read coverage across each chromosome against chromosome 1 revealed significant p-values demonstrating uneven chromosome copy number. Stringent cut-offs for ploidy differences relative to the largest chromosome (Chr. 1) of each isolate were chosen: p<5^−10^. Chromosomes with p-values below this cut-off, with a mean depth that is greater than chromosome 1 are highlighted in blue, while those with a mean depth lower than chromosome 1 are shown in green. All 308 chromosomal p-values (excluding chr1) are shown in the bottom plot ordered from smallest to greatest.
(PNG)

Figure S6 The percent of reads specifying the two most frequent alleles per chromosome using 2 representative isolates from each lineage of *Bd*. The most common allele is shown in black and the second most common allele is shown in blue. Bins were used to summarise the expected peaks for odd, even and odd numbers of chromosomes and shown in red (lines show bin value cut-offs and dots show values). Individual chromosomes with a predominantly bi-allelic value are shown with a blue border, and those with a predominant tri-allelic value are shown with a black border.
(PNG)

Figure S7 Sliding non-overlapping windows of 10 Kb across the 22 *Bd* nuclear genomes showing homozygous SNPs minus heterozygous positions. Predominance of homozygous SNPs is shown in red and predominance of heterozygous positions in shown in blue. Windows across *Bd*GPL isolates demonstrate highly uneven distribution of heterozygosity attributed to recombination whereas polymorphisms are more evenly spread across the genomes of *Bd*CAPE and *Bd*CH isolates.
(PNG)

Figure S8 Heterozygous positions had their phase determined using overlapping reads. Reads from each isolate are shown as a separate black line on the graphs. Only bi-allelic polymorphisms were compared for phasing. Predominantly, overlapping reads agreed with a single bi-allelic phase. (**A**) All reads over all phased positions. A 90% cut-off was used to filter ambiguous phased positions or those with an excess of mismatches as shown by the red line. (**B**) Positions that agreed 90–100% for a single phase are shown as a percent of all reads.
(PNG)

Figure S9 Illustrations of how phased haplotypes were extracted from the alignment. Heterozygous positions that did not pass the minimum depth or percent phased cut-offs, along with examples of pairwise crossovers and outcomes for a four-gamete test between three isolates.
(PNG)

Figure S10 Pairwise comparisons for shared phased heterozygous positions. (**A**) Total numbers of matching phased heterozygous positions in same phase (Kb) (**B**) Percent of matching phase positions from the total number of shared phased positions.
(PNG)

Figure S11 Phased heterozygous positions demonstrating crossovers were identified between every isolate. (**A**) Total numbers of crossovers identified. (**B**) Percent of crossovers from the total number of shared phased positions.
(PNG)

Figure S12 Lengths of haplotypes (in nucleotides) that included at least two alleles per loci in every isolate of a given group, and were therefore suitable for population genetic analysis. *Bd*GPL subset (s.s.) 1 consists of isolates VC1, AP15 and JEL423. Subset 2 consists of subset 1, ETH4 and MODS27.
(PNG)

Figure S13 Intra-lineage heterozygote's, the percent of heterozygote's that were phased (PP), the percent of PP's that demonstrated a crossover (XO) and the RbarD were plotted using non-overlapping windows across the genome (length 10 Kb). Both phased positions and crossovers were found across each of the chromosomes in each of the lineages of *Bd*, suggesting recombination is not confined to small or large chromosomes, or the ends of any given chromosome. The same is seen with rBarD values.
(PNG)

Figure S14 The Fixation Index (*F*_ST_) was calculated for each pairwise lineage across window lengths of 1.4 Kb (**A**) and 10 Kb (**B**). All three lineages are differentiated from one another across each chromosome, with some intra-chromosomal variation. Notably, the stretch of rDNA located at the start of chromosome 14 appears to have a reduced genetic distance between each of the three lineages of *Bd*. *Bd*GPL subset (ss) 1 consists of isolates VC1, AP15 and JEL423. Subset 2 consists of subset 1, ETH4 and MODS27.
(PNG)

Figure S15 The total numbers of crossovers found within genes demonstrated variation between gene families. All crossovers were compared against total number of heterozygous and phased positions, transcript length and tribe size. Proteases and chitin recognition proteins had a greater number of crossovers than would be expected by random over their combined number of phased positions.
(PNG)

Figure S16 Crossovers at unique locations (non-redundant, NR) occurred differentially across gene families. NR crossovers were compared against total number of heterozygous and phased positions, transcript length and tribe size. Proteases and chitin recognition proteins had a greater number of crossovers than would be expected by random over their combined number of phased positions.
(PNG)

Figure S17 The ratio of non-synonymous mutation per non-synonymous site (*dN*) vs synonymous mutation per synonymous site (*dS*) from alignments to *Bd* JEL423 for each of the gene families for all isolates belonging to the *Bd*GPL. The line designates the ω value (*dN*/*dS*), whereby everything above the line has ω>1 and represents genes undergoing the greatest levels of variation.
(PNG)

Figure S18 The ratio of non-synonymous mutation per non-synonymous site (*dN*) vs synonymous mutation per synonymous site (*dS*) from alignments to *Bd* JEL423 for each of the gene families for all isolates belonging to the three lineages. The lines designate the ω value (*dN*/*dS*), whereby everything above the line has ω>1 and represents genes undergoing the greatest levels of variation. Summaries of ω values for all genes in each of the three lineages are shown in the final three plots.
(PNG)

Figure S19 A Venn diagram showing the total number of genes undergoing positive selection according to the Branch site model (BSM), where genes had 2D′>8.1887. The nine genes were identified in all three lineages were four uncharacterised (secreted) with transcript ID's 05565, 02533, 00379, 06783 and five uncharacterised (non-secreted) with transcript ID's 03962, 07794, 05877, 02935, 08088.
(PNG)

Table S1 Polymorphisms and reference bases were identified in 22 *Bd* nuclear genomes relative to *Bd* JEL423 using BiSCaP v0.11 with default settings. (**A**) Tallies of each category of loci found in each separate isolate. (**B**) The percent of uniquely mapped reads over each type of category of loci. Bi-allelic heterozygous positions had a reduced percent of uniquely mapped reads in the 2 divergent lineages of *Bd*, which may result from structural variants. Additionally, 72.48% of the homozygous SNPs and heterozygous positions were phased, which came from reads >86% uniquely mapped to the genome in any given isolate.
(PNG)

Table S2 The two most common allele frequencies over each base of each chromosome were determined by percent of read agreement with the reference base. Using 1000 Bootstrap replicates of these values, we recorded how often 47–53% reads agreeing with an allele predominated over 30–36% or 63–69% reads agreeing with an allele. Shown in white are chromosomes with >95% of replicates showing a predominantly bi-allele signature (even-ploidies). Chromosomes with <5% bootstrap support for an even number of chromosomes therefore had a high support for unbalanced allele frequencies (odd-ploidies), and shown in blue. Chromosomes not fulfilling these criteria are shown in green and considered ambiguous.
(PNG)

Table S3 Genes were tested for enrichment in non-redundant (NR; at unique loci) crossovers (XO) and NR XO/NR phased position (NRPP) compared to the values for all genes using Hypergeometric tests and *t*-tests respectively. For *t*-tests, all genes with <2 NRPP (the minimum required for a crossover) were excluded. Although both CRN-like and uncharacterised (secreted) were enriched for crossovers at unique loci (non-redundant), only CRN-like (between lineages) were enriched for XO/NRPP.
(PNG)

Table S4 Haplotypes over coding sequence that failed the four-gamete test were predominantly from coding-regions. Haplotypes overlapping a number of genes were included in the counts for each gene (385 extra counts to total number of haplotypes). After accounting for these extra counts, an additional 1,162 haplotypes were still found to come from coding regions compared with those from intergenic or intron regions. However, no gene group had a clear enrichment for haplotypes that failed the four-gamete test. *Bd*GPL subset (s.s.) 1 consisted of isolates VC1, AP15 and JEL423. Subset 2 consisted of subset 1, ETH4 and MODS27.
(PNG)

Table S5 Only five presence absence (PA) polymorphisms relative to *Bd*GPL JEL423 were identified amongst *Bd*CAPE and *Bd*CH isolates, whilst none were identified amongst *Bd*GPL isolates.
(PNG)

Table S6 Homozygous (A) and bi-allelic heterozygous (B) polymorphisms were found in the coding and non-coding regions of the *Bd* nuclear genomes. The total numbers of each variant-type are followed by their numbers per kilobase of genomic region in parentheses. For heterozygous positions, the affect on the transcript (synonymous/non-synonymous) was determined using the alternative allele. Where two alternative alleles to the reference sequence were found (infrequently), the first present within the VCF was chosen. With the exception of the reference strain *Bd* JEL423, the ratios of non-synonymous to synonymous changes were between 1.12–2.00 and 1.22–2.13 for homozygous and heterozygous positions respectively.
(PNG)

Table S7 ABC transporters, Chitin associated genes and CRN-like genes were tested for enrichment in homozygous SNPs. The total number, average and standard deviation of non-redundant homozygous SNPs for each gene family were calculated for all isolates, and lineage specific isolates. A Hypergeometric test was used to identify significant enrichment for variants where P<0.01 (*), P<0.001 (**) and P<0.0001 (***).
(PNG)

Table S8 Proteases, uncharacterized secreted genes and uncharacterized genes were tested for enrichment in homozygous SNPs. The total number, average and standard deviation of non-redundant homozygous SNPs for each gene family were calculated for all isolates, and lineage specific isolates. A Hypergeometric test was used to identify significant enrichment for variants where P<0.01 (*), P<0.001 (**) and P<0.0001 (***).
(PNG)

Table S9 Secreted and CRN-like genes are significantly enriched for heterozygous positions at unique loci. The total number, average and standard deviation of non-redundant heterozygous and phased positions for each gene family were calculated for all isolates and lineage specific isolates. A Hypergeometric test was used to identify significant enrichment for heterozygosity where P<0.01 (*), P<0.001 (**) and P<0.0001 (***).
(PNG)

Table S10 The average rates of synonymous substitution (*dS*), non-synonymous substitution (*dN*) and omega (*dN*/*dS* = ω) for every gene in every isolate.
(PNG)

Table S11 Number and category of genes with ω≥1 (1,450 in total) that also were found to have undergone positive selection using the Branch Site Models in codeml. (**A**) The total numbers of genes, the numbers of genes with ω≥1 among all isolates, and how many of those genes had 2D′≥11.4076 (1% significance after Bonferroni correction) and 11.4076>2D′>8.1887 (5% significance after Bonferroni correction). The final column shows the percent of genes with 2D′>8.1887 from those with ω≥1. (**B**) For each lineage, the numbers of genes with ω≥1 and those that also had 2D′>8.1887. Following both of these columns are the results from a hypergeometric test for enrichment. For the genes with ω≥1, the test is for enrichment from the entire set of genes, whilst for the genes with 2D′>8.1887, the test is for enrichment from just the genes with ω≥1. (**C**) Overlap of genes with 2D′>8.1887.
(PNG)

Text S1 Supplemental Materials and Methods. Full details of methods and analysis described in this manuscript.
(DOC)

## References

[1] Fisher MC, Henk DA, Briggs CJ, Brownstein JS, Madoff LC, et al (2012) Emerging fungal threats to animal, plant and ecosystem health. Nature 484: 186–194. 22498624 10.1038/nature10947 PMC3821985

[2] Raffaele R, Kamoun S (2012) Genome evolution in filamentous plant pathogens: why bigger can be better. Nat Rev Microbiol 10: 417–430. 22565130 10.1038/nrmicro2790

[3] Ma L, van der Does HC, Borkovich KA, Coleman JJ, Daboussi M, et al (2010) Comparative genomics reveals mobile pathogenicity chromosomes in *Fusarium* . Nature 464: 367–73. 20237561 10.1038/nature08850 PMC3048781

[4] Win J, Morgan W, Bos J, Krasileva KV, Cano LM, et al (2007) Adaptive evolution has targeted the C-terminal domain of the RXLR effectors of plant pathogenic Oomycetes. Plant Cell 19: 2349–2369. 17675403 10.1105/tpc.107.051037 PMC2002621

[5] Heitman J (2010) Evolution of eukaryotic microbial pathogens via covert sexual reproduction. Cell Host Microbe 8: 86–99. 20638645 10.1016/j.chom.2010.06.011 PMC2916653

[6] Wittenberg AHJ, van der Lee TAJ, M'Barek SB, Ware SB, Goodwin SB, et al (2009) Meiosis drives extraordinary genome plasticity in the haploid fungal plant pathogen *Mycosphaerella graminicola* . PLoS ONE 4: e5863. 19516898 10.1371/journal.pone.0005863 PMC2689623

[7] Cooke DEL, Cano LM, Raffaele S, Bain RA, Cooke LR, et al (2012) Genome analyses of an aggressive and invasive lineage of the Irish potato famine pathogen. PLoS Pathog 8: e1002940. 23055926 10.1371/journal.ppat.1002940 PMC3464212

[8] Büttner P, Koch F, Voigt K, Quidde T, Risch S, et al (1994) Variations in ploidy among isolates of *Botrytis cinerea*: implications for genetic and molecular analyses. Curr Genet 25: 445–50. 8082191 10.1007/BF00351784

[9] Carr J, Shearer G Jr (1998) Genome size, complexity, and ploidy of the pathogenic fungus *Histoplasma capsulatum* . J Bacteriol 180: 6697–6703. 9852017 10.1128/jb.180.24.6697-6703.1998 PMC107776

[10] Sheltzer JM, Blank HM, Pfau SJ, Tange Y, George BM, et al (2011) Aneuploidy drives genomic instability in Yeast. Science 333: 1026–30. 21852501 10.1126/science.1206412 PMC3278960

[11] Abbey D, Hickman M, Gresham D, Berman J (2011) High-resolution SNP/CGH microarrays reveal the accumulation of loss of heterozygosity in commonly used *Candida albicans* strains. G3: Genes Genomes Genetics 1: 523–30. 22384363 10.1534/g3.111.000885 PMC3276171

[12] Lengeler KB, Cox GM, Heitman J (2001) Serotype AD strains of *Cryptococcus neoformans* are diploid or aneuploid and are heterozygous at the mating-type locus. Infect Immun 69: 115–22. 11119496 10.1128/IAI.69.1.115-122.2001 PMC97862

[13] Hu G, Wang J, Choi J, Jung WH, Liu I, et al (2011) Variation in chromosome copy number influences the virulence of *Cryptococcus neoformans* and occurs in isolates from AIDS patients. BMC Genomics 12: 526. 22032296 10.1186/1471-2164-12-526 PMC3221739

[14] Sionov E, Lee H, Chang YC, Kwon-Chung KJ (2010) *Cryptococcus neoformans* overcomes stress of azole drugs by formation of disomy in specific multiple chromosomes. PLoS Pathog 6: e1000848. 20368972 10.1371/journal.ppat.1000848 PMC2848560

[15] Reedy JL, Floyd AM, Heitman J (2009) Mechanistic plasticity of sexual reproduction and meiosis in the *Candida* pathogenic species complex. Curr Biol 19: 891–9. 19446455 10.1016/j.cub.2009.04.058 PMC2788334

[16] Forche A, Magee PT, Selmecki A, Berman J, May G (2009) Evolution in *Candida albicans* populations during a single passage through a mouse host. Genetics 182: 799–811. 19414562 10.1534/genetics.109.103325 PMC2710160

[17] Kwon-Chung KJ, Chang YC (2012) Aneuploidy and drug resistance in pathogenic fungi. PLoS Path 8: e1003022. 10.1371/journal.ppat.1003022 PMC3499572 23166494

[18] Forche A, Alby K, Schaefer D, Johnson AD, Berman J, et al (2008) The parasexual cycle in *Candida albicans* provides an alternative pathway to meiosis for the formation of recombinant strains. PLoS Biol 6: e110. 18462019 10.1371/journal.pbio.0060110 PMC2365976

[19] Arnold B, Bomblies K, Wakeley J (2012) Extending coalescent theory to autotetraploids. Genetics 192: 195–204. 22714411 10.1534/genetics.112.140582 PMC3430536

[20] Schoustra SE, Debets AJM, Slakhorst M, Hoekstra RF (2007) Mitotic recombination accelerates adaptation in the fungus *Aspergillus nidulans* . PLoS Genet 3: e68. 17465683 10.1371/journal.pgen.0030068 PMC1857732

[21] Fisher MC, Garner TWJ, Walker SF (2009) Global emergence of *Batrachochytrium dendrobatidis* and amphibian chytridiomycosis in space, time, and host. Annu Rev of Microbiol 63: 291–310. 19575560 10.1146/annurev.micro.091208.073435

[22] Farrer RA, Weinert LA, Bielby J, Garner TWJ, Balloux F, et al (2011) Multiple emergence of genetically diverse amphibian-infecting chytrids include a globalised hypervirulent lineage. Proc Natl Acad Sci U S A 108: 18732–6. 22065772 10.1073/pnas.1111915108 PMC3219125

[23] Goka K, Yokoyama J, Une Y, Kuroki T, Suzuki K, et al (2009) Amphibian chytridiomycosis in Japan: Distribution, haplotypes and possible route of entry into Japan. Mol Ecol 18: 4757–74. 19840263 10.1111/j.1365-294X.2009.04384.x

[24] Schloegel LM, Toledo LF, Longcore JE, Greenspan SE, Vieira CA, et al (2012) Novel, panzootic and hybrid genotypes of amphibian chytridiomycosis associated with the bullfrog trade. Mol Ecol 21: 5162–5177. 22857789 10.1111/j.1365-294X.2012.05710.x

[25] *Batrachochytrium dendrobatidis* Sequencing Project, Broad Institute of Harvard and MIT (http://www.broadinstitute.org/).

[26] Li H, Durbin R (2009) Fast and accurate short read alignment with Burrows-Wheeler transform. Bioinformatics 25: 1754–60. 19451168 10.1093/bioinformatics/btp324 PMC2705234

[27] Farrer RA, Henk DA, MacLean D, Studholme DJ, Fisher MC (2013) Using false discovery rates to benchmark SNP-callers in next-generation sequencing projects. Sci Rep 3: 1512. 23518929 10.1038/srep01512 PMC3604800

[28] Morgan JAT, Vredenburg VT, Rachowicz LJ, Knapp RA, Stice MJ, et al (2007) Population genetics of the frog-killing fungus *Batrachochytrium dendrobatidis* . Proc Natl Acad Sci USA 104: 13845–50. 17693553 10.1073/pnas.0701838104 PMC1945010

[29] Agapow P, Burt A (2001) Indices of multilocus linkage disequilibrium. Mol Ecol Notes 1: 101–2.

[30] Hudson R, Kaplan N (1985) Statistical properties of the number of recombination events in the history of a sample of sequences. Genetics 111: 147–164. 4029609 10.1093/genetics/111.1.147 PMC1202594

[31] Weir BS (1996) Genetic Data Analysis II. Sinauer, Sunderland.

[32] Joneson S, Stajich JE, Shiu SH, Rosenblum EB (2011) Genomic transition to pathogenicity in chytrid fungi. PLoS Pathog 7: e1002338. 22072962 10.1371/journal.ppat.1002338 PMC3207900

[33] Sun G, Yang Z, Kosch T, Summers K, Huang J (2011) Evidence for acquisition of virulence effectors in pathogenic chytrids. BMC Evol Biol 11: 195. 21740557 10.1186/1471-2148-11-195 PMC3161006

[34] Abramyan J, Stajich JE (2012) Species-specific chitin binding module 18 (CBM18) expansion in the amphibian pathogen *Batrachochytrium dendrobatidis* . mBio 3: e00150–12. 22718849 10.1128/mBio.00150-12 PMC3569864

[35] Rosenblum EB, James TY, Zamudio KR, Poorten TJ, Rodriguez D, et al (2013) Complex history of the amphibian-killing chytrid fungus revealed with genome resequencing data. Proc Natl Acad Sci U S A 110: 9385–9390. 23650365 10.1073/pnas.1300130110 PMC3677446

[36] James TY, Litvintseva AP, Vilgalys R, Morgan JAT, Taylor JW, et al (2009) Rapid global expansion of the fungal disease chytridiomycosis into declining and healthy amphibian populations. PLoS Pathog 5: 1–12. 10.1371/journal.ppat.1000458 PMC2680619 19478871

[37] Rosenblum EB, Poorten TJ, Joneson S, Settles M (2012) Substrate-specific gene expression in *Batrachochytrium dendrobatidis*, the chytrid pathogen of amphibians. PLoS ONE 7: e49924. 23185485 10.1371/journal.pone.0049924 PMC3502224

[38] Woodhams DC, Alford RA, Briggs CJ, Johnson M, Rollins-Smith LA (2008) Life-history trade-offs influence disease in changing climates: strategies of an amphibian pathogen. Ecology 89: 1627–39. 18589527 10.1890/06-1842.1

[39] Li H, Handsaker B, Wysoker A, Fennell T, Ruan J, et al (2009) The Sequence Alignment/Map format and SAMtools. Bioinformatics 25: 2078–9. 19505943 10.1093/bioinformatics/btp352 PMC2723002

[40] Drummond AJ, Rambaut A (2007) BEAST: Bayesian evolutionary analysis by sampling trees. BMC Evol Biol 7: 214. 17996036 10.1186/1471-2148-7-214 PMC2247476

[41] Bendtsen JD, Nielsen H, Heijne GV, Brunak S (2004) Improved prediction of signal peptides: SignalP 3.0. J Mol Biol 340: 783–795. 15223320 10.1016/j.jmb.2004.05.028

[42] Rawlings ND, Barrett AJ, Bateman A (2010) MEROPS: the peptidase database. Nucleic Acids Res 38: D227–D233. 19892822 10.1093/nar/gkp971 PMC2808883

[43] Malik A, Firoz A, Jha V, Ahmad S (2010) PROCARB: A Database of Known and Modelled Carbohydrate-Binding Protein Structures with Sequence-Based Prediction Tools. Adv Bioinformatics 436036. 20671979 10.1155/2010/436036 PMC2909730

[44] Yang Z (2007) PAML 4: Phylogenetic Analysis by Maximum Likelihood. Mol Biol Evol 24: 1586–91. 17483113 10.1093/molbev/msm088

[45] Yang Z, Nielsen R (2000) Estimating Synonymous and Nonsynonymous Substitution Rates Under Realistic Evolutionary Models. Mol Biol Evol 17: 32–43. 10666704 10.1093/oxfordjournals.molbev.a026236

[46] Daum JM, Davis LR, Bigler L, Woodhams DC (2012) Hybrid advantage in skin peptide immune defenses of water frogs (*Pelophylax esculentus*) at risk from emerging pathogens. Infect Genet Evol 12: 1854–1864. 22940461 10.1016/j.meegid.2012.07.024

[47] Berger L, Speare R, Daszak P, Green DE, Cunningham AA, et al (1998) Chytridiomycosis causes amphibian mortality associated with population declines in the rain forests of Australia and central America. Proc Natl Acad Sci U S A 95: 9031–6. 9671799 10.1073/pnas.95.15.9031 PMC21197

