# Supplementary figures and images for: Chromosomal Copy Number Variation, Selection and Uneven Rates of Recombination Reveal Cryptic Genome Diversity Linked to Pathogenicity
Source: PLoS Genet. 2013 Aug 15;9(8):e1003703. doi: 10.1371/journal.pgen.1003703 (PMC3744429; doi:10.1371/journal.pgen.1003703)

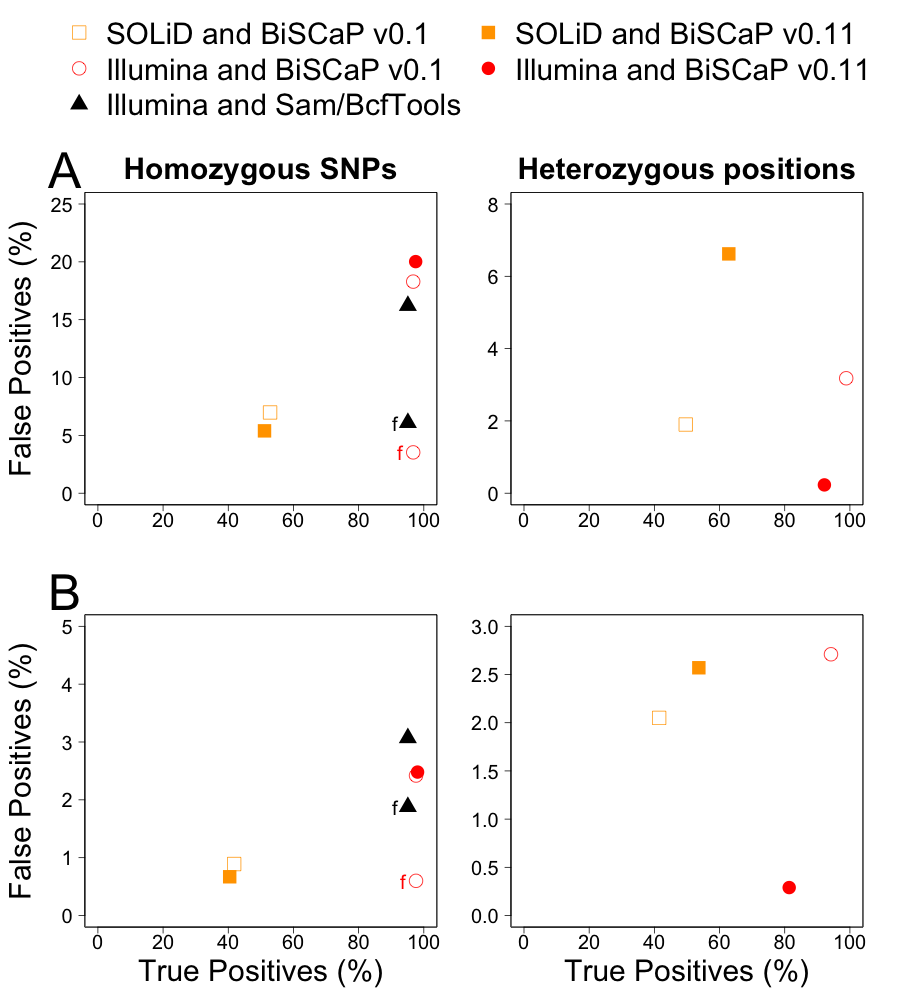

Supplement: Figure S1 — The previous SOLiD reads (5) and the new Illumina paired end reads of Bd isolate JEL423 were aligned to a modified JEL423 reference sequence. Additionally, simulated reads from a heterozygous reference sequence were made to the depths of the Illumina and SOLiD datasets. Single Nucleotide Polymorphisms (SNPs) and heterozygous positions were then called and the False Discovery Rates (FDR) ascertained. The SNP-caller BiSCaP v0.11 was tested using default settings, and SAM/BCFTools with VCFUtils was tested for its ability to call SNPs using its default settings. SNPs were also filtered for those found without first modifying the reference sequence (f = filtered). (A) 1 nt/Kb simulated SNPs or heterozygous positions (12,458 in total) within the coding region (CDS) (B) 1 nt/100 nt simulated SNPs or heterozygous positions (124,588 in total) within the CDS region. The new Illumina data was able to recover >95% of true positive SNPs and >80% true positive heterozygous positions using BiSCaP v0.11, outperforming the previous lower-depth SOLiD sequences. (PNG) [file pgen.1003703.s001.png]

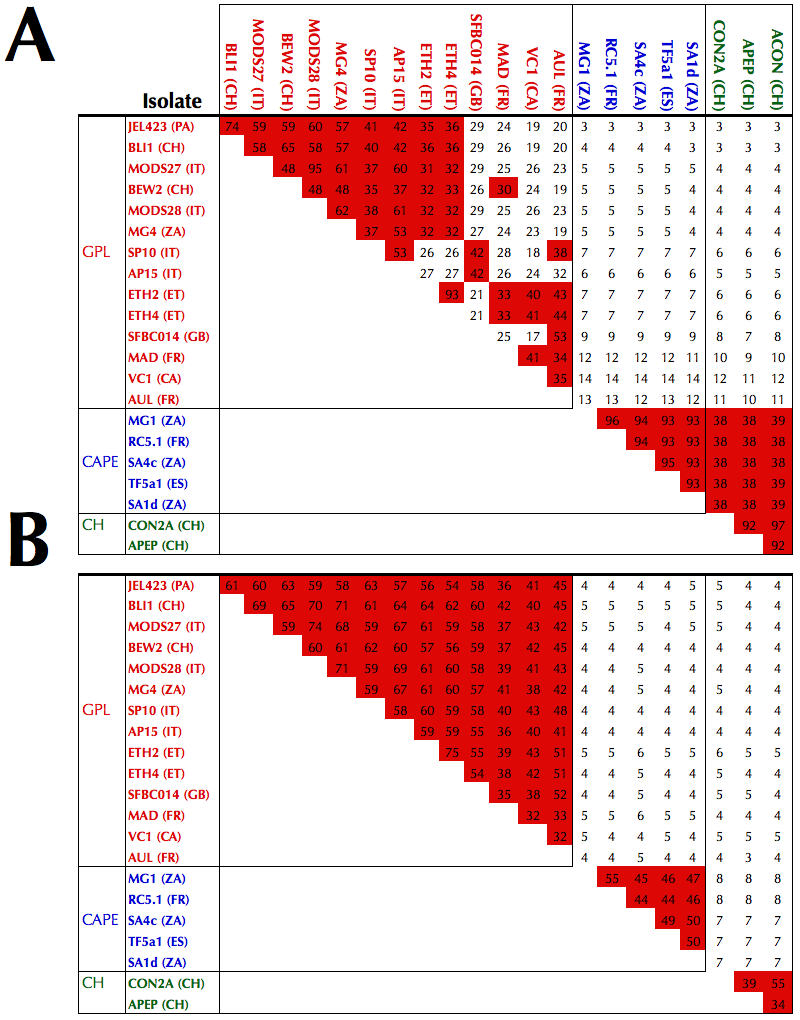

Supplement: Figure S2 — The percent of ECVA polymorphic sites shared between each of the 22 isolates. Greater overlap (≥30%) highlighted in red. (A) The overlap of homozygous SNPs varied between 3% and 97% (B) The overlap of heterozygous positions varied between 3% and 75%. (PNG) [file pgen.1003703.s002.png]

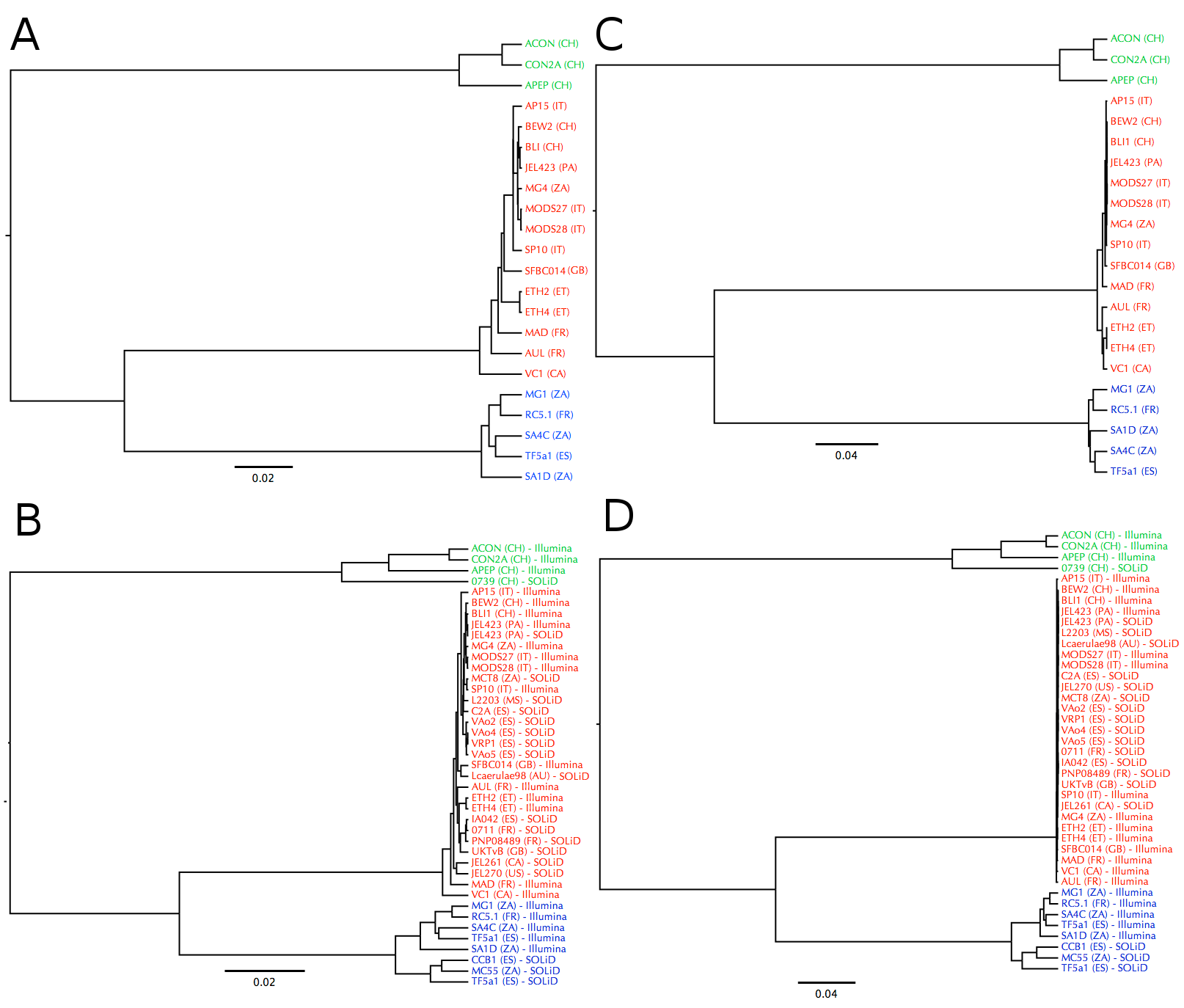

Supplement: Figure S3 — Phylogenetic trees were made using the UPGMA algorithm in PAUP from ECVA polymorphic positions identified in the nuclear genomes demonstrating three divergent lineages (BdGPL, BdCAPE and BdCH shown in red blue and green respectively). (A) A tree from 275 Kb ECVA polymorphic positions identified from Illumina sequencing. (B) A tree from 36 Kb ECVA polymorphic positions from Illumina and SOLiD sequencing. (C) A tree from 218 Kb EVCA homozygous positions identified from Illumina sequencing. (D) A tree from 8 Kb EVCA homozygous positions identified from Illumina and SOLiD sequencing. (PNG) [file pgen.1003703.s003.png]

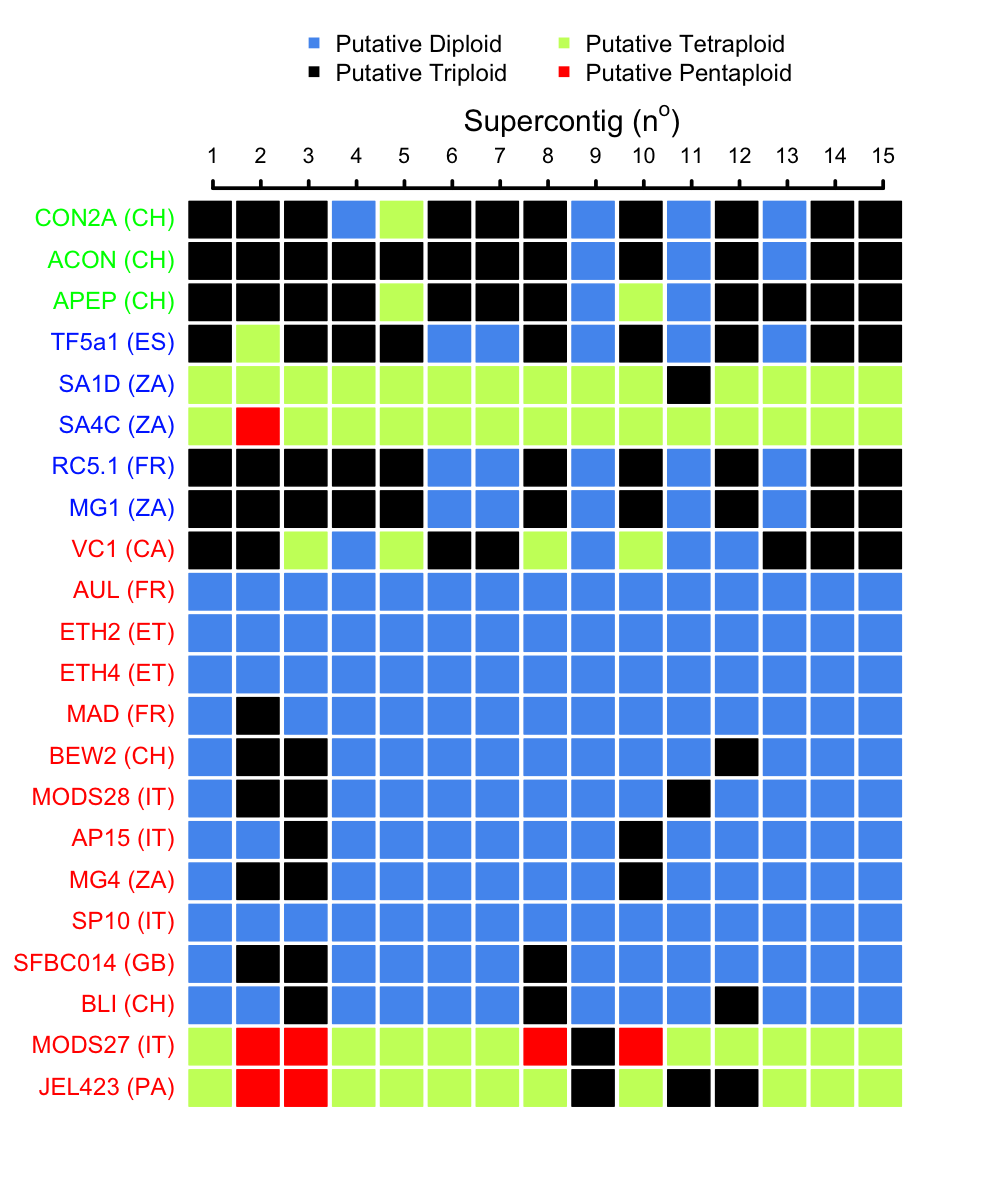

Supplement: Figure S4 — CCNV in the Bd nuclear genomes was identified using allele-frequencies and mean read depths across each chromosome normalised to the alignment depth for each isolate. Many BdGPL isolates can be seen to include more copies of chromosome 2 and 3, while the 3 BdCH and 3 of the 5 BdCAPE isolates have fewer copies of chromosome 9 and 11. Fewer copies of chromosome 9, 11 and 16 appear to be found in many of the isolates. (PNG) [file pgen.1003703.s004.png]

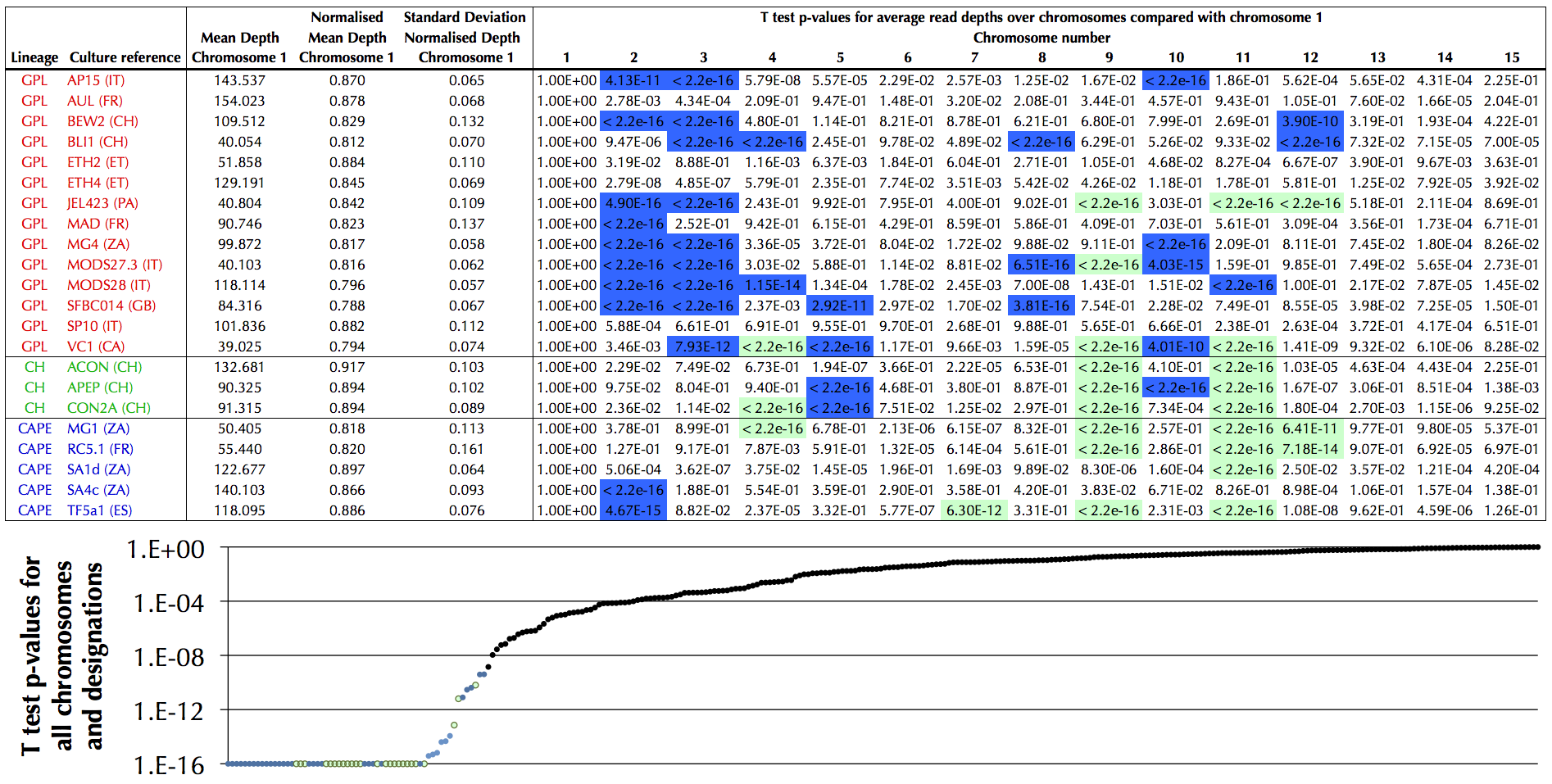

Supplement: Figure S5 — t-tests for the mean depth of read coverage across each chromosome against chromosome 1 revealed significant p-values demonstrating uneven chromosome copy number. Stringent cut-offs for ploidy differences relative to the largest chromosome (Chr. 1) of each isolate were chosen: p<5−10. Chromosomes with p-values below this cut-off, with a mean depth that is greater than chromosome 1 are highlighted in blue, while those with a mean depth lower than chromosome 1 are shown in green. All 308 chromosomal p-values (excluding chr1) are shown in the bottom plot ordered from smallest to greatest. (PNG) [file pgen.1003703.s005.png]

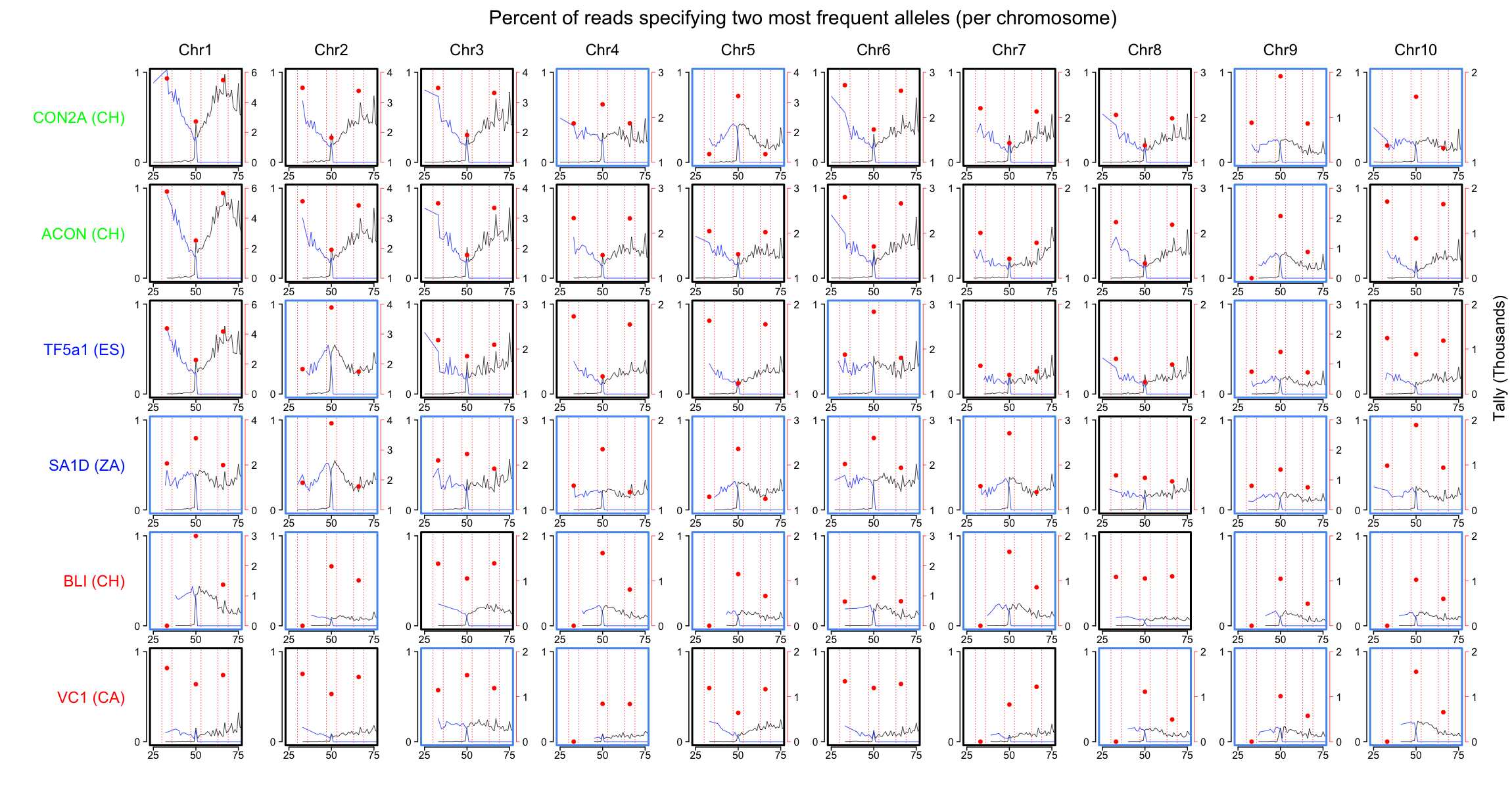

Supplement: Figure S6 — The percent of reads specifying the two most frequent alleles per chromosome using 2 representative isolates from each lineage of Bd. The most common allele is shown in black and the second most common allele is shown in blue. Bins were used to summarise the expected peaks for odd, even and odd numbers of chromosomes and shown in red (lines show bin value cut-offs and dots show values). Individual chromosomes with a predominantly bi-allelic value are shown with a blue border, and those with a predominant tri-allelic value are shown with a black border. (PNG) [file pgen.1003703.s006.png]

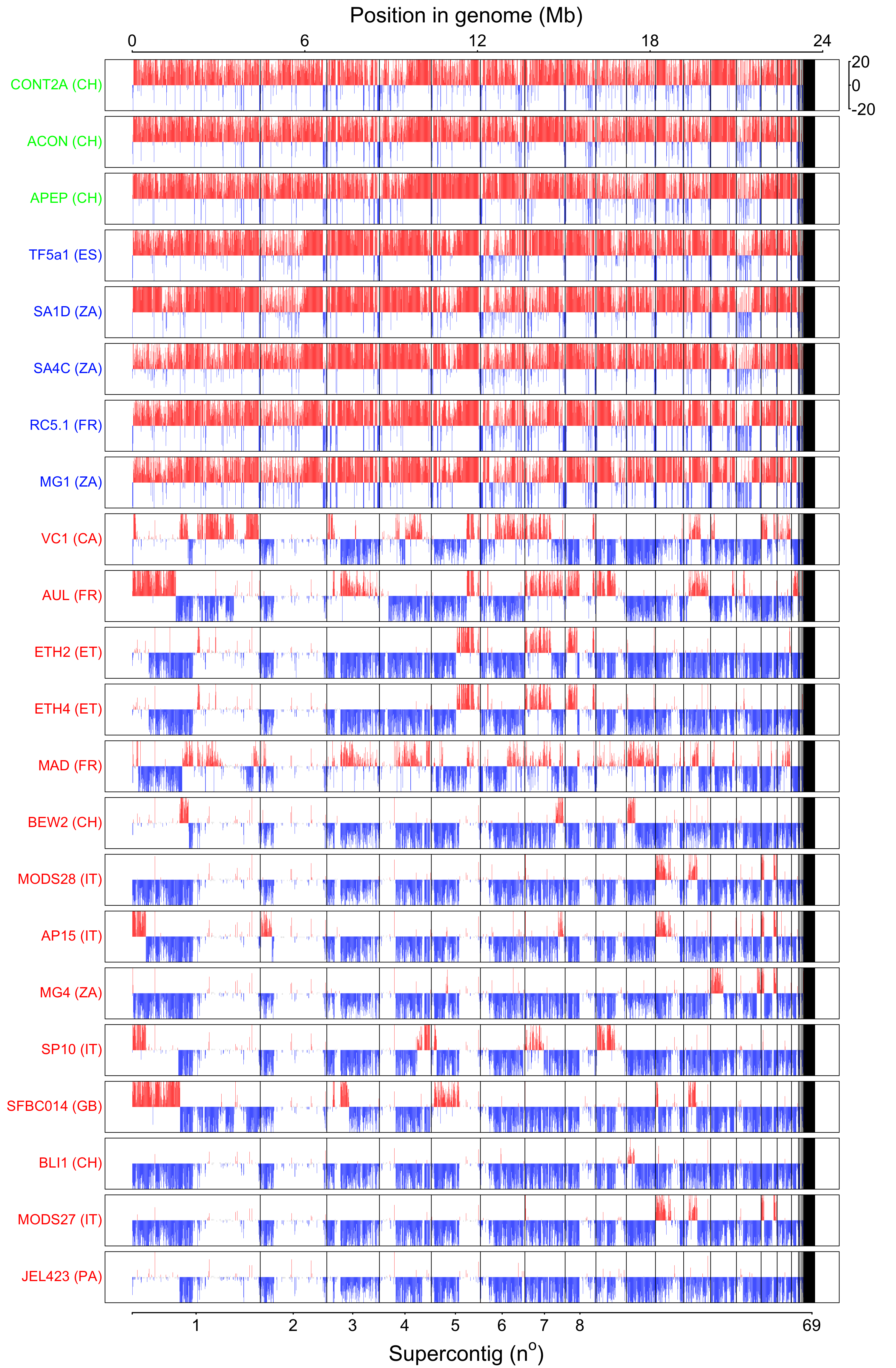

Supplement: Figure S7 — Sliding non-overlapping windows of 10 Kb across the 22 Bd nuclear genomes showing homozygous SNPs minus heterozygous positions. Predominance of homozygous SNPs is shown in red and predominance of heterozygous positions in shown in blue. Windows across BdGPL isolates demonstrate highly uneven distribution of heterozygosity attributed to recombination whereas polymorphisms are more evenly spread across the genomes of BdCAPE and BdCH isolates. (PNG) [file pgen.1003703.s007.png]

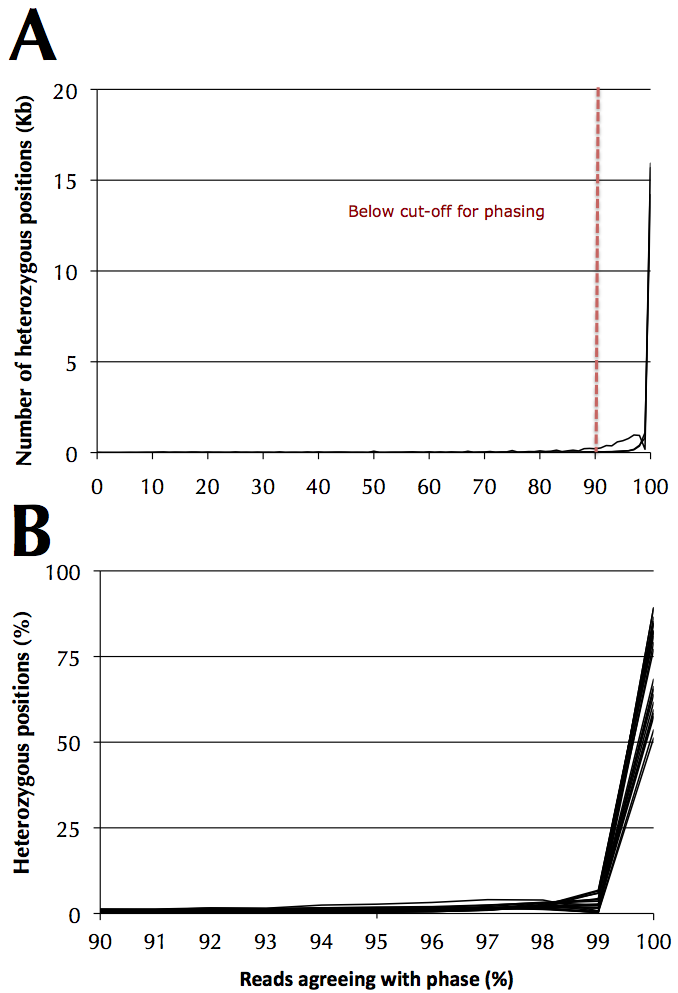

Supplement: Figure S8 — Heterozygous positions had their phase determined using overlapping reads. Reads from each isolate are shown as a separate black line on the graphs. Only bi-allelic polymorphisms were compared for phasing. Predominantly, overlapping reads agreed with a single bi-allelic phase. (A) All reads over all phased positions. A 90% cut-off was used to filter ambiguous phased positions or those with an excess of mismatches as shown by the red line. (B) Positions that agreed 90–100% for a single phase are shown as a percent of all reads. (PNG) [file pgen.1003703.s008.png]

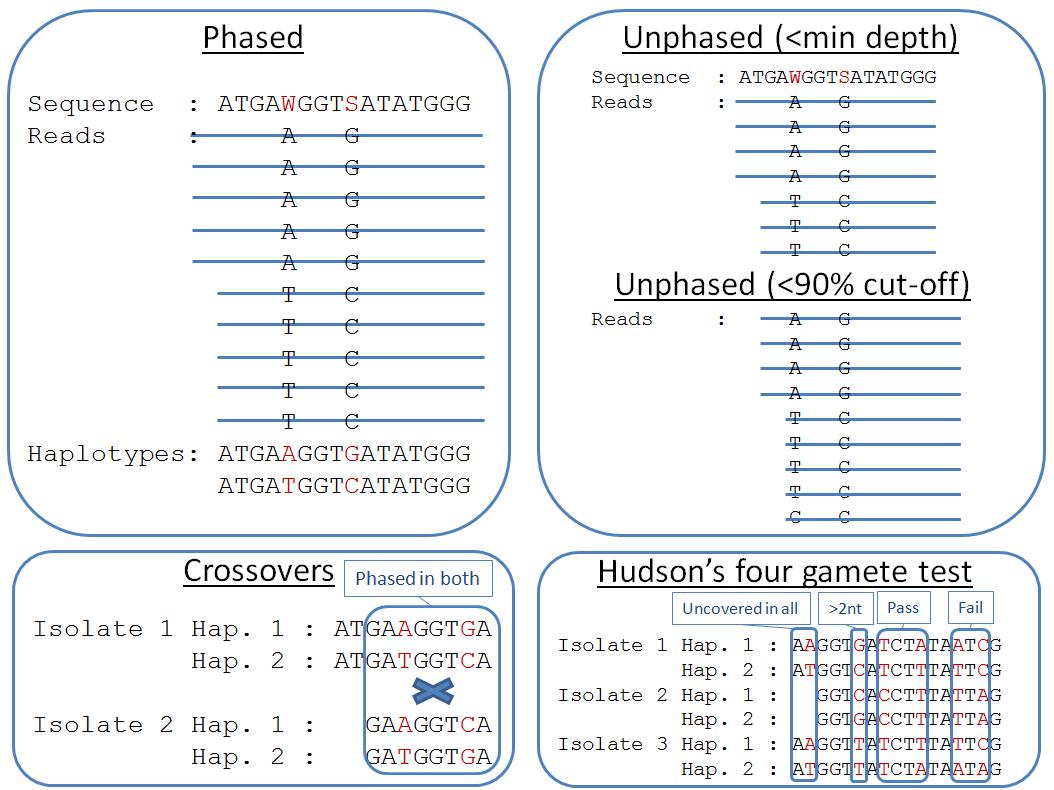

Supplement: Figure S9 — Illustrations of how phased haplotypes were extracted from the alignment. Heterozygous positions that did not pass the minimum depth or percent phased cut-offs, along with examples of pairwise crossovers and outcomes for a four-gamete test between three isolates. (PNG) [file pgen.1003703.s009.png]

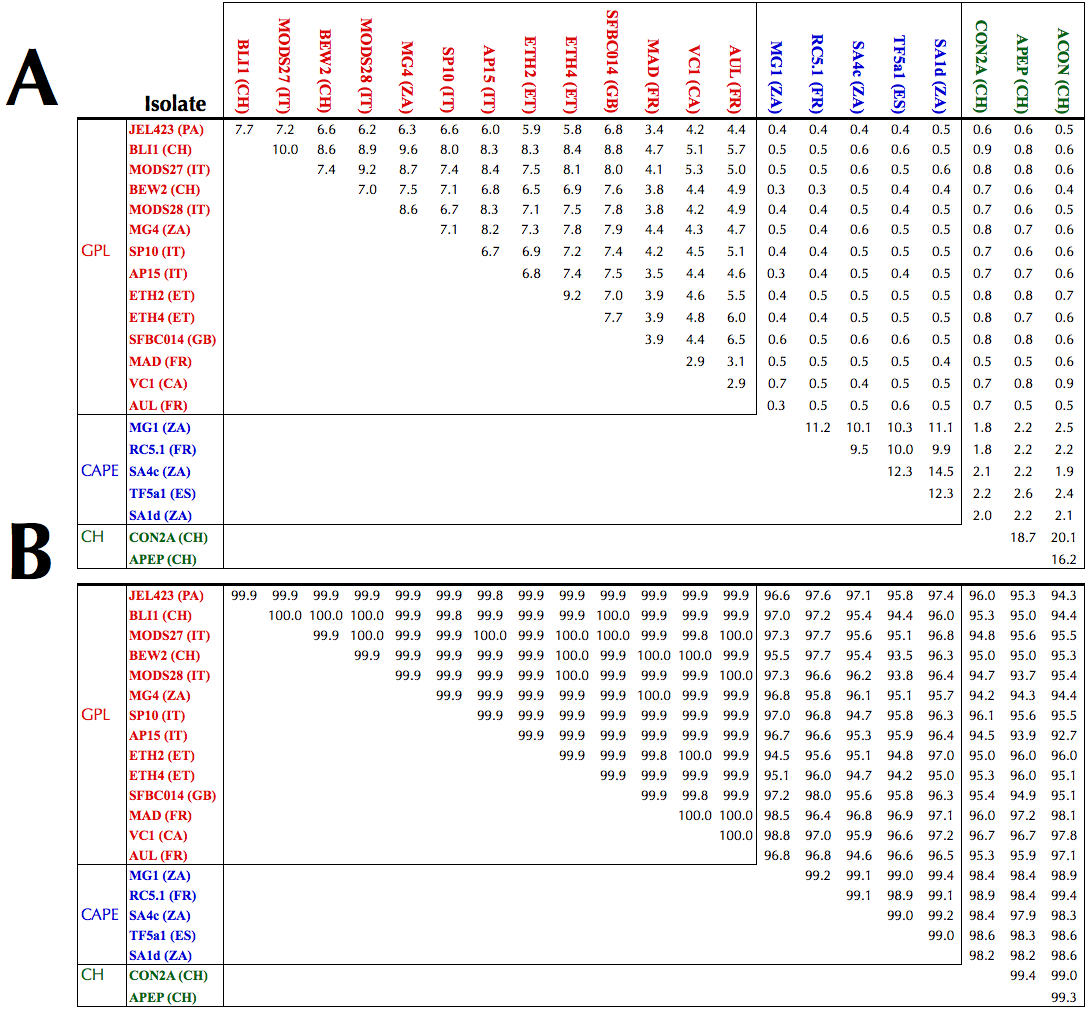

Supplement: Figure S10 — Pairwise comparisons for shared phased heterozygous positions. (A) Total numbers of matching phased heterozygous positions in same phase (Kb) (B) Percent of matching phase positions from the total number of shared phased positions. (PNG) [file pgen.1003703.s010.png]

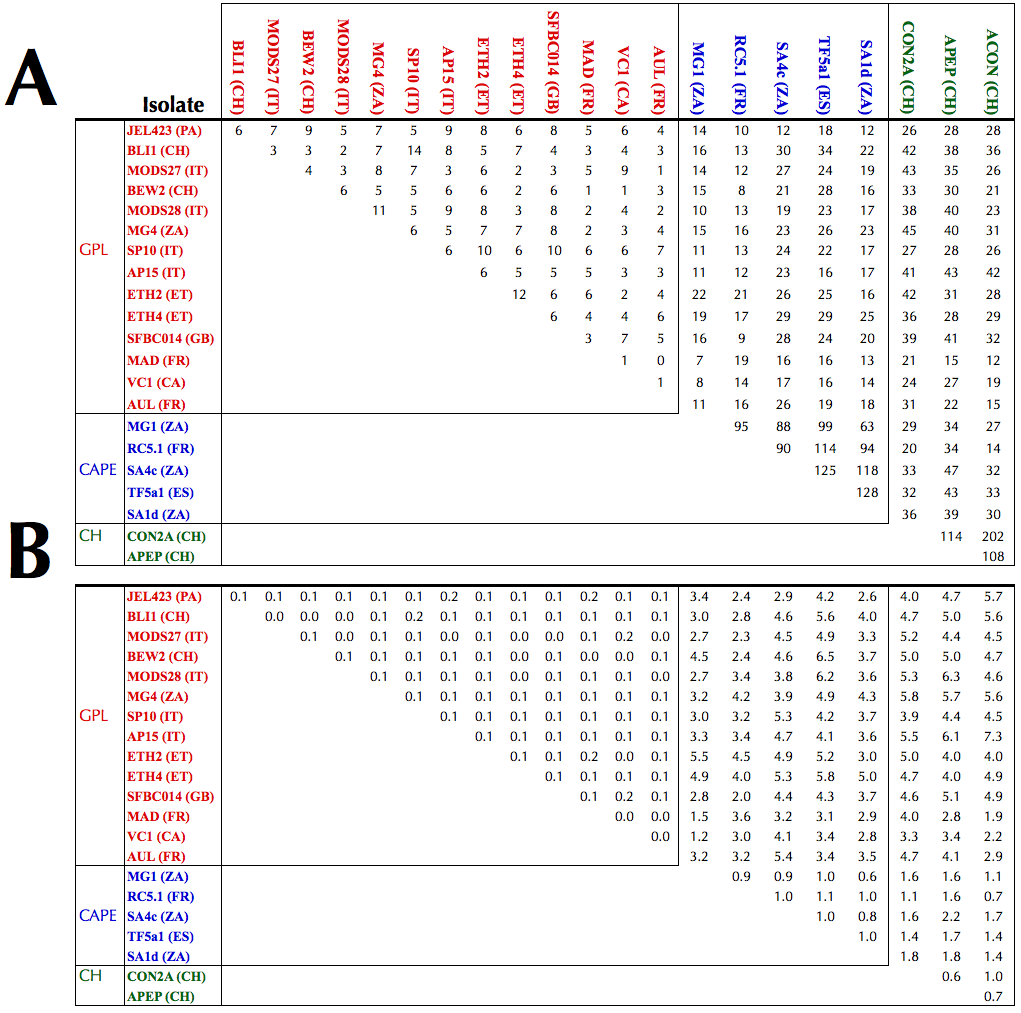

Supplement: Figure S11 — Phased heterozygous positions demonstrating crossovers were identified between every isolate. (A) Total numbers of crossovers identified. (B) Percent of crossovers from the total number of shared phased positions. (PNG) [file pgen.1003703.s011.png]

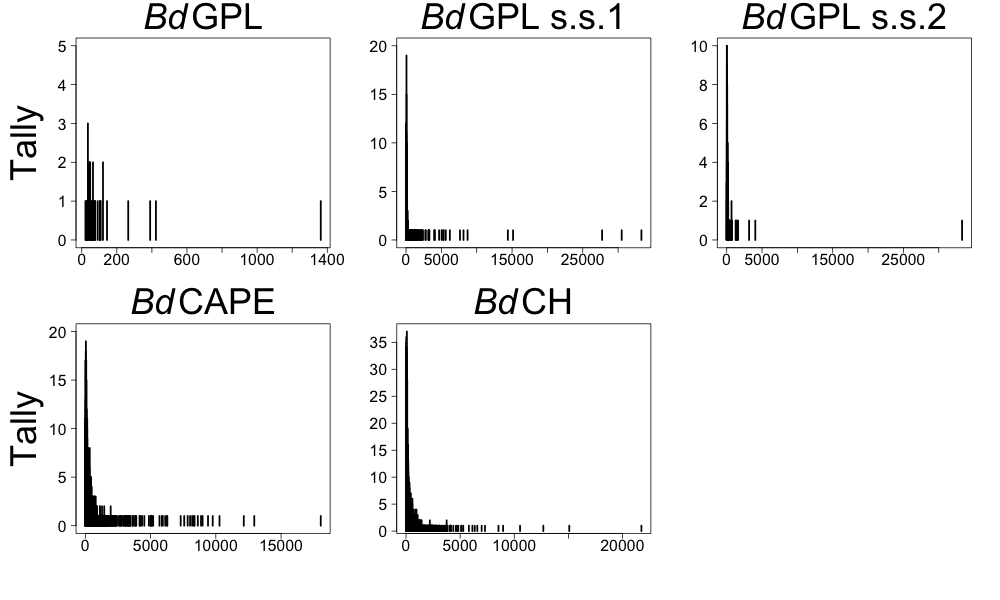

Supplement: Figure S12 — Lengths of haplotypes (in nucleotides) that included at least two alleles per loci in every isolate of a given group, and were therefore suitable for population genetic analysis. BdGPL subset (s.s.) 1 consists of isolates VC1, AP15 and JEL423. Subset 2 consists of subset 1, ETH4 and MODS27. (PNG) [file pgen.1003703.s012.png]

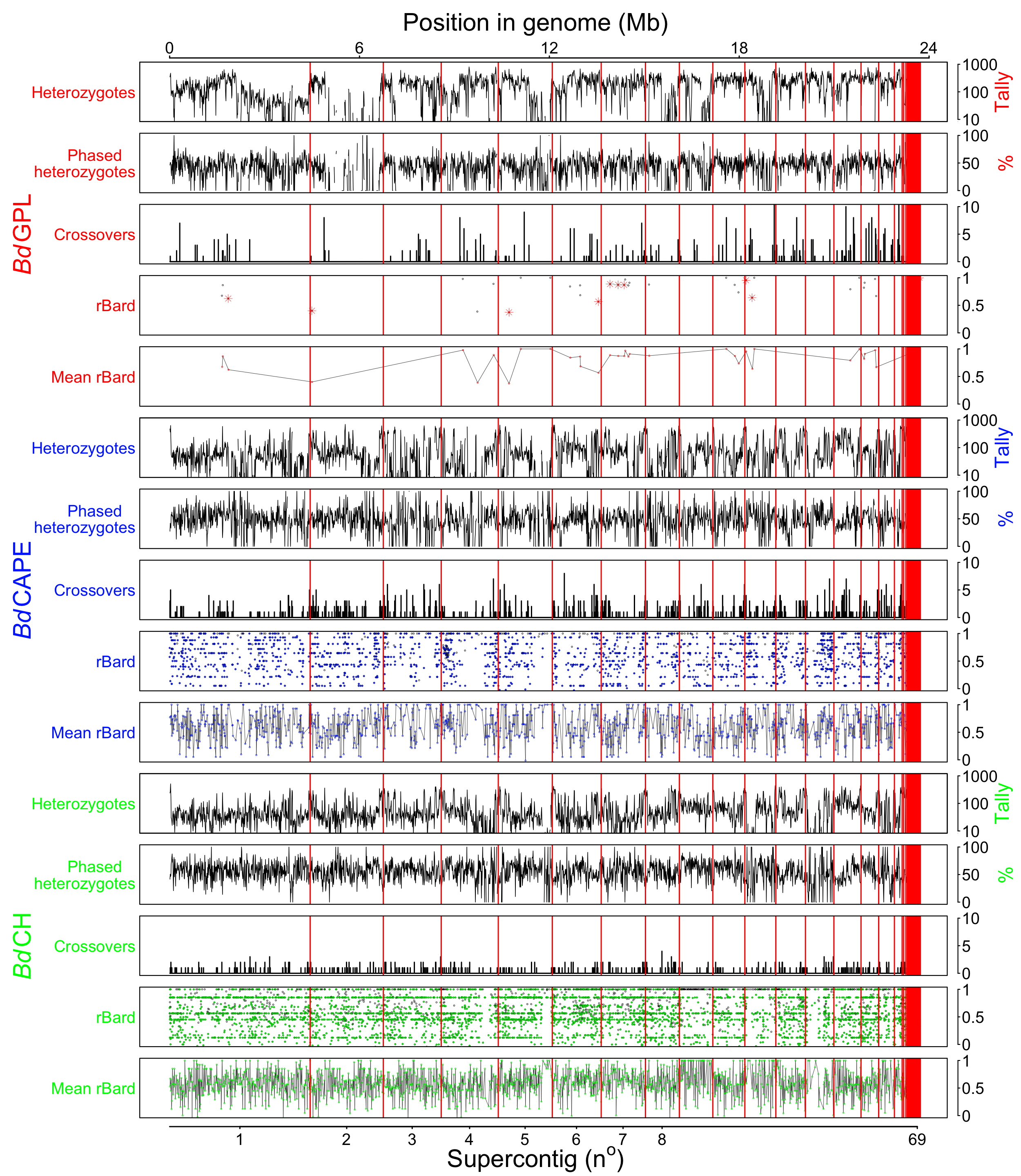

Supplement: Figure S13 — Intra-lineage heterozygote's, the percent of heterozygote's that were phased (PP), the percent of PP's that demonstrated a crossover (XO) and the RbarD were plotted using non-overlapping windows across the genome (length 10 Kb). Both phased positions and crossovers were found across each of the chromosomes in each of the lineages of Bd, suggesting recombination is not confined to small or large chromosomes, or the ends of any given chromosome. The same is seen with rBarD values. (PNG) [file pgen.1003703.s013.png]

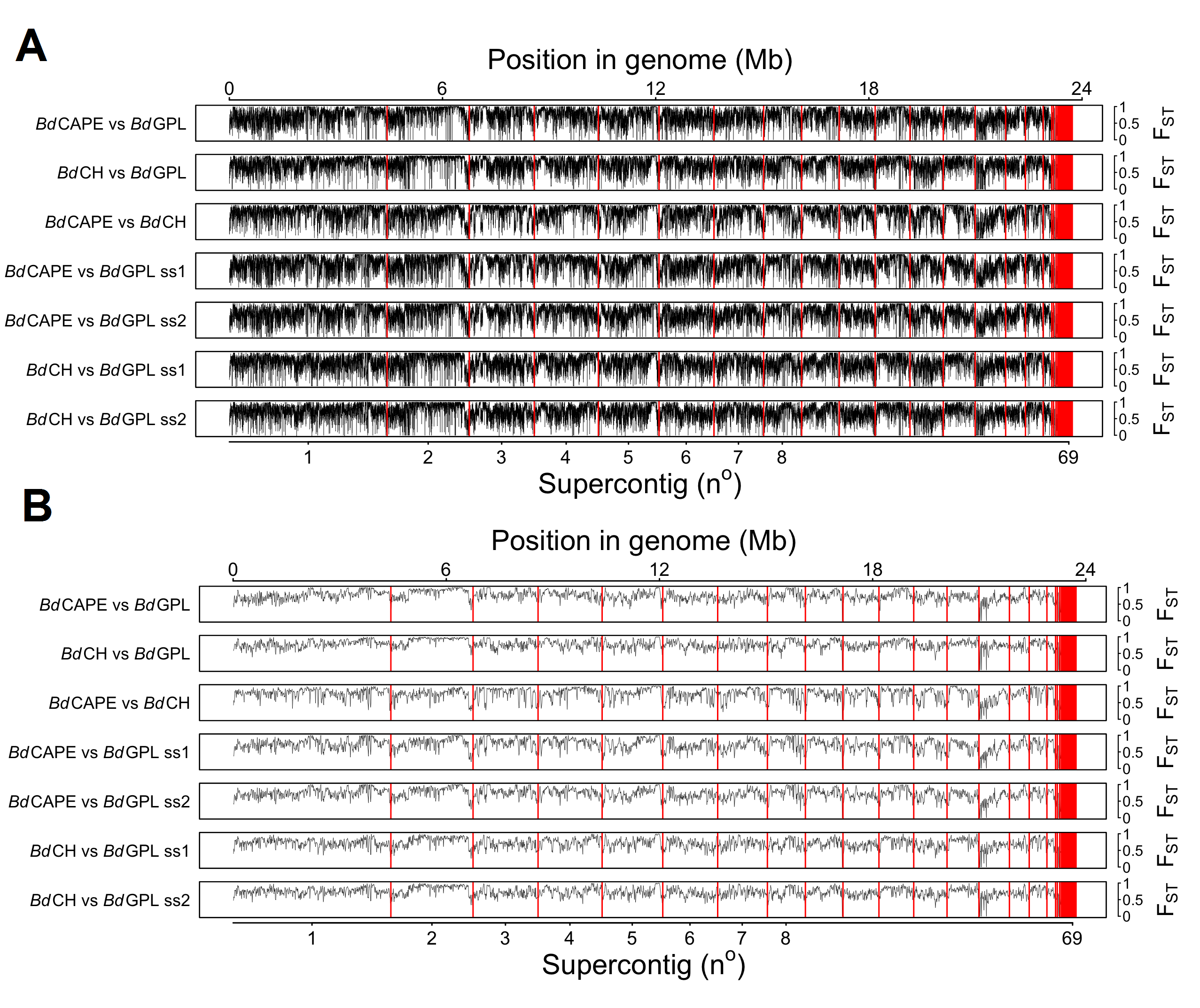

Supplement: Figure S14 — The Fixation Index (F ST) was calculated for each pairwise lineage across window lengths of 1.4 Kb (A) and 10 Kb (B). All three lineages are differentiated from one another across each chromosome, with some intra-chromosomal variation. Notably, the stretch of rDNA located at the start of chromosome 14 appears to have a reduced genetic distance between each of the three lineages of Bd. BdGPL subset (ss) 1 consists of isolates VC1, AP15 and JEL423. Subset 2 consists of subset 1, ETH4 and MODS27. (PNG) [file pgen.1003703.s014.png]

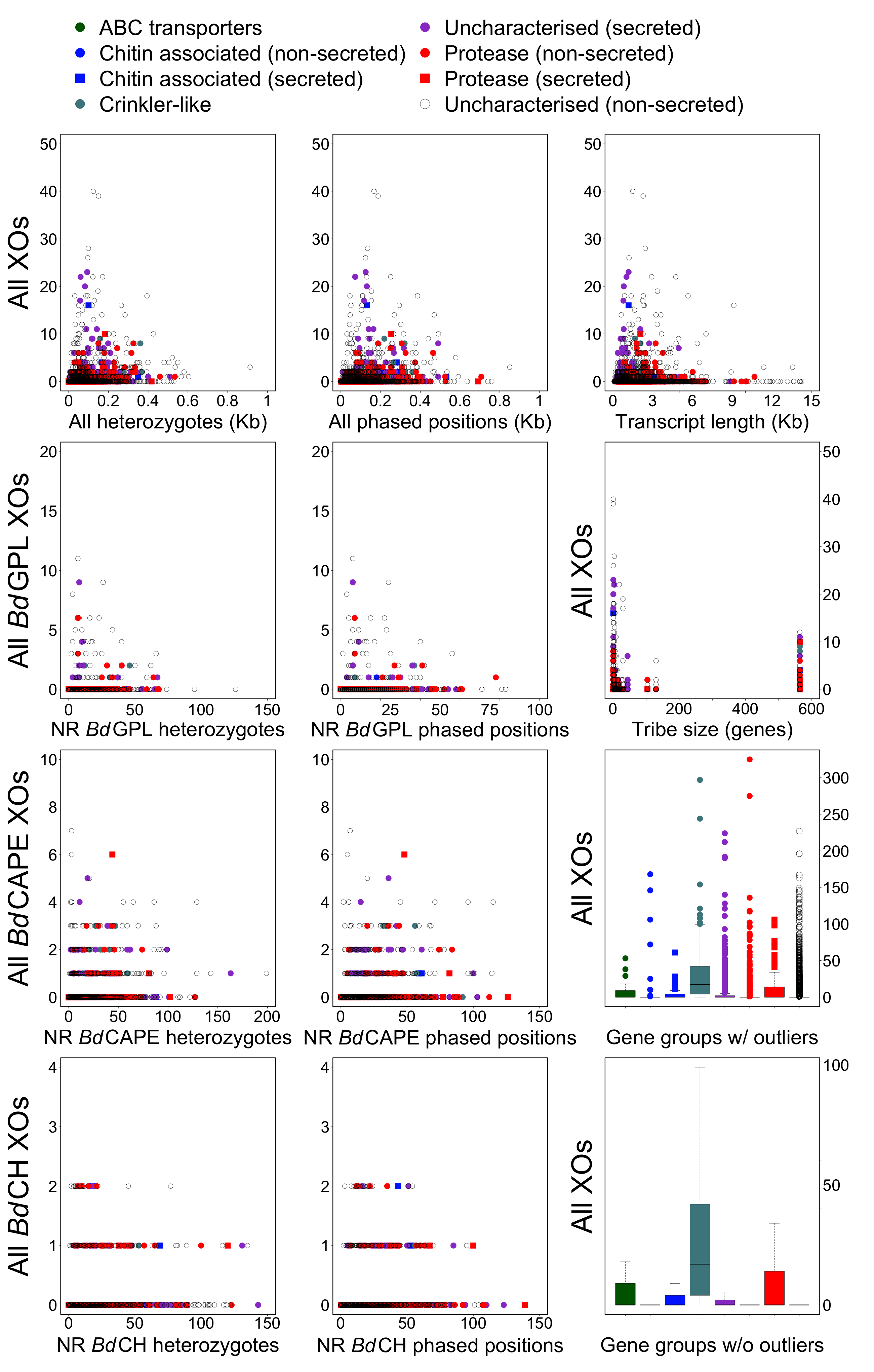

Supplement: Figure S15 — The total numbers of crossovers found within genes demonstrated variation between gene families. All crossovers were compared against total number of heterozygous and phased positions, transcript length and tribe size. Proteases and chitin recognition proteins had a greater number of crossovers than would be expected by random over their combined number of phased positions. (PNG) [file pgen.1003703.s015.png]

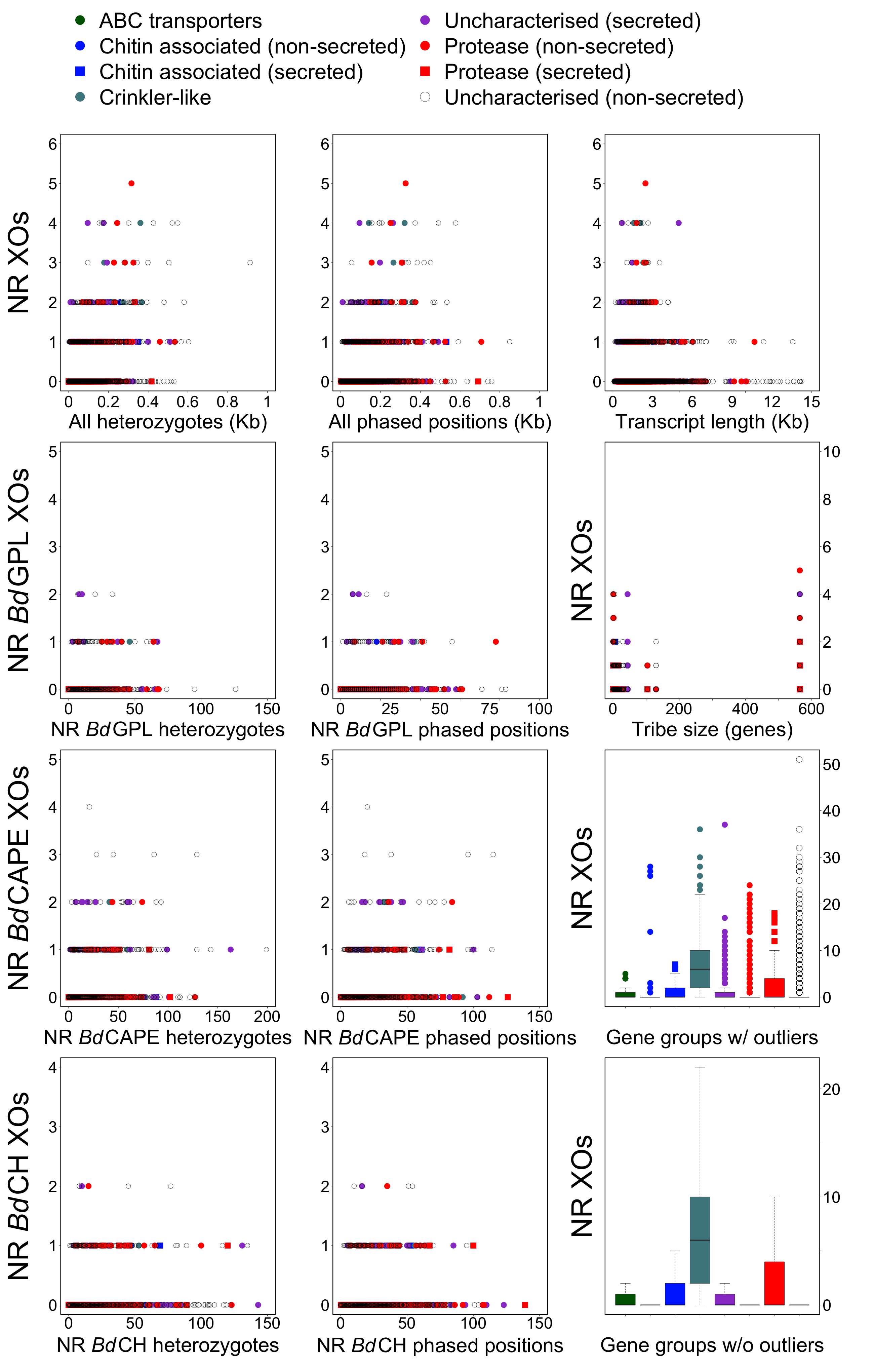

Supplement: Figure S16 — Crossovers at unique locations (non-redundant, NR) occurred differentially across gene families. NR crossovers were compared against total number of heterozygous and phased positions, transcript length and tribe size. Proteases and chitin recognition proteins had a greater number of crossovers than would be expected by random over their combined number of phased positions. (PNG) [file pgen.1003703.s016.png]

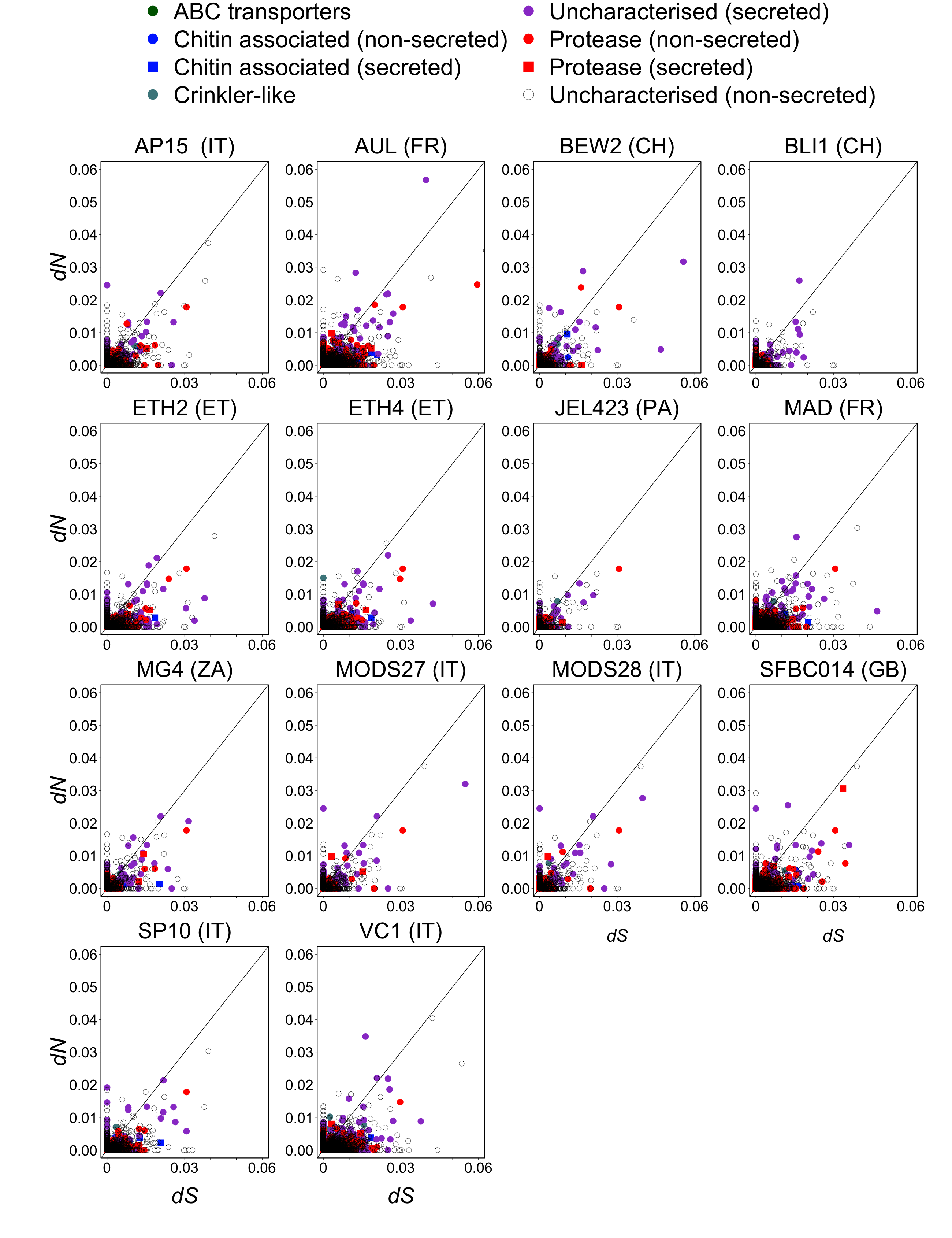

Supplement: Figure S17 — The ratio of non-synonymous mutation per non-synonymous site (dN) vs synonymous mutation per synonymous site (dS) from alignments to Bd JEL423 for each of the gene families for all isolates belonging to the BdGPL. The line designates the ω value (dN/dS), whereby everything above the line has ω>1 and represents genes undergoing the greatest levels of variation. (PNG) [file pgen.1003703.s017.png]

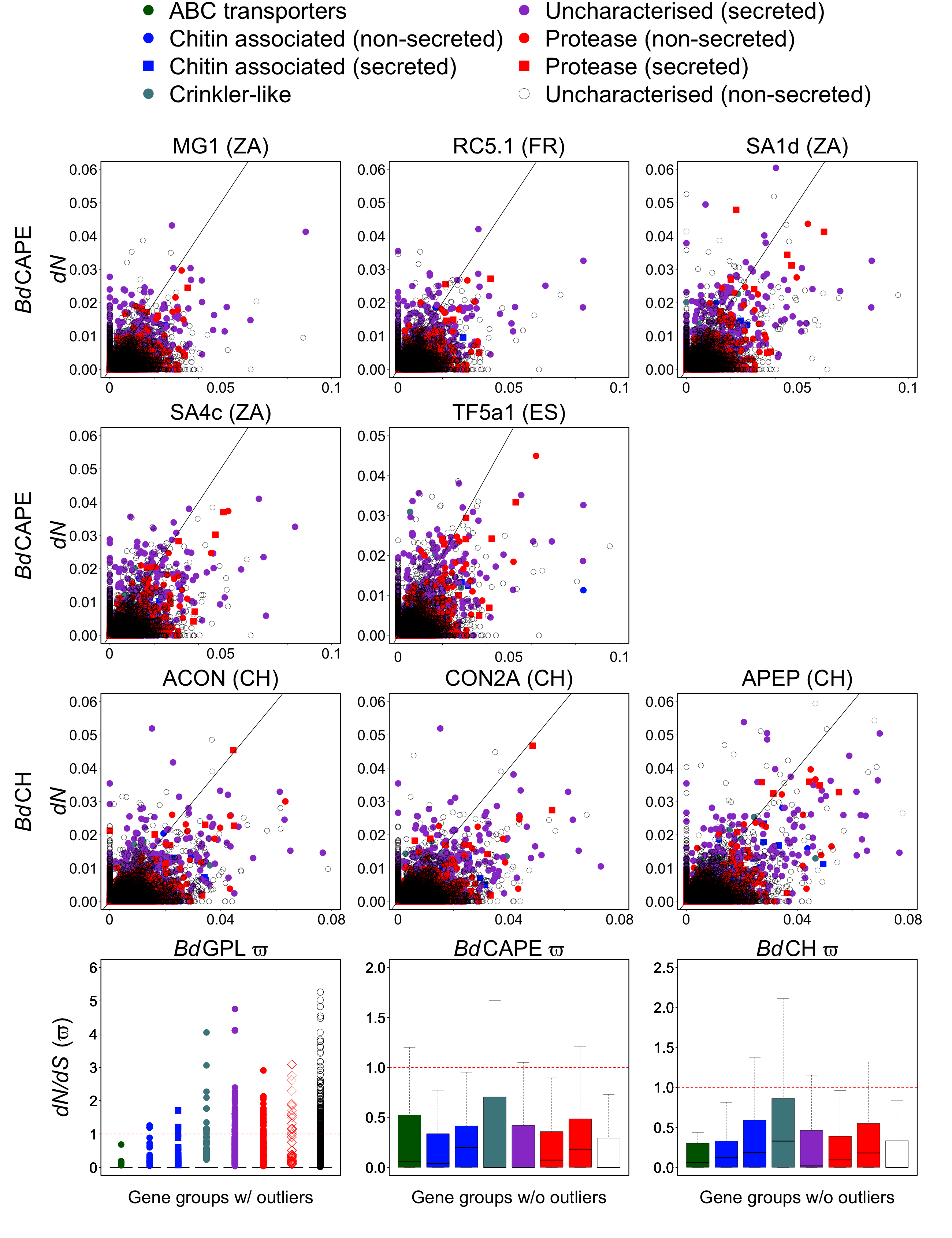

Supplement: Figure S18 — The ratio of non-synonymous mutation per non-synonymous site (dN) vs synonymous mutation per synonymous site (dS) from alignments to Bd JEL423 for each of the gene families for all isolates belonging to the three lineages. The lines designate the ω value (dN/dS), whereby everything above the line has ω>1 and represents genes undergoing the greatest levels of variation. Summaries of ω values for all genes in each of the three lineages are shown in the final three plots. (PNG) [file pgen.1003703.s018.png]

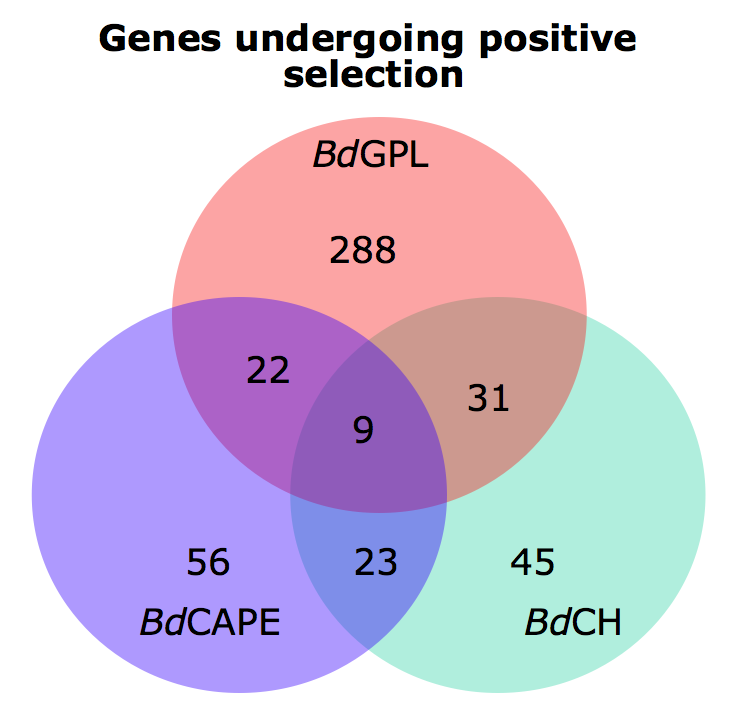

Supplement: Figure S19 — A Venn diagram showing the total number of genes undergoing positive selection according to the Branch site model (BSM), where genes had 2D′>8.1887. The nine genes were identified in all three lineages were four uncharacterised (secreted) with transcript ID's 05565, 02533, 00379, 06783 and five uncharacterised (non-secreted) with transcript ID's 03962, 07794, 05877, 02935, 08088. (PNG) [file pgen.1003703.s019.png]

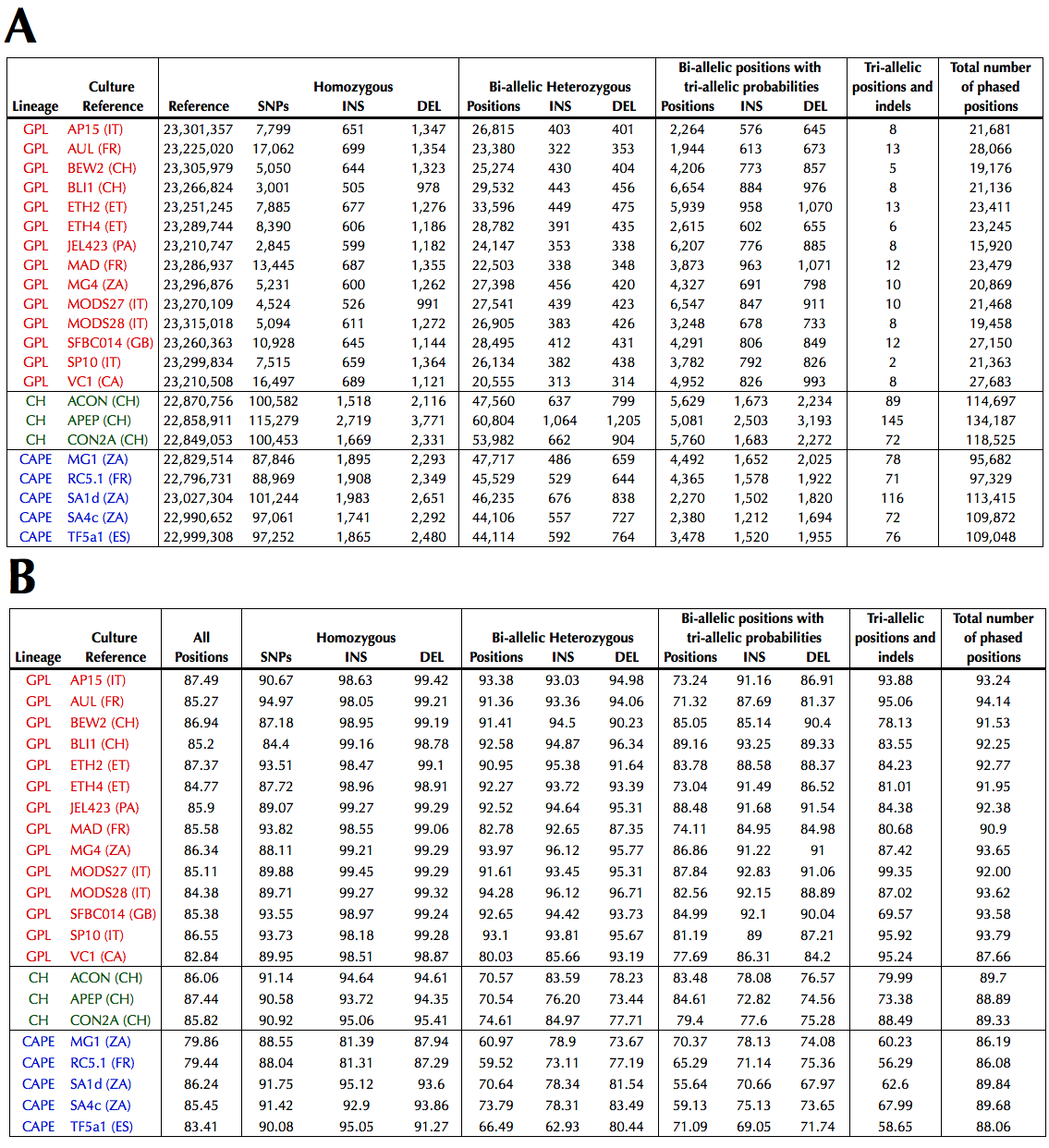

Supplement: Table S1 — Polymorphisms and reference bases were identified in 22 Bd nuclear genomes relative to Bd JEL423 using BiSCaP v0.11 with default settings. (A) Tallies of each category of loci found in each separate isolate. (B) The percent of uniquely mapped reads over each type of category of loci. Bi-allelic heterozygous positions had a reduced percent of uniquely mapped reads in the 2 divergent lineages of Bd, which may result from structural variants. Additionally, 72.48% of the homozygous SNPs and heterozygous positions were phased, which came from reads >86% uniquely mapped to the genome in any given isolate. (PNG) [file pgen.1003703.s020.png]

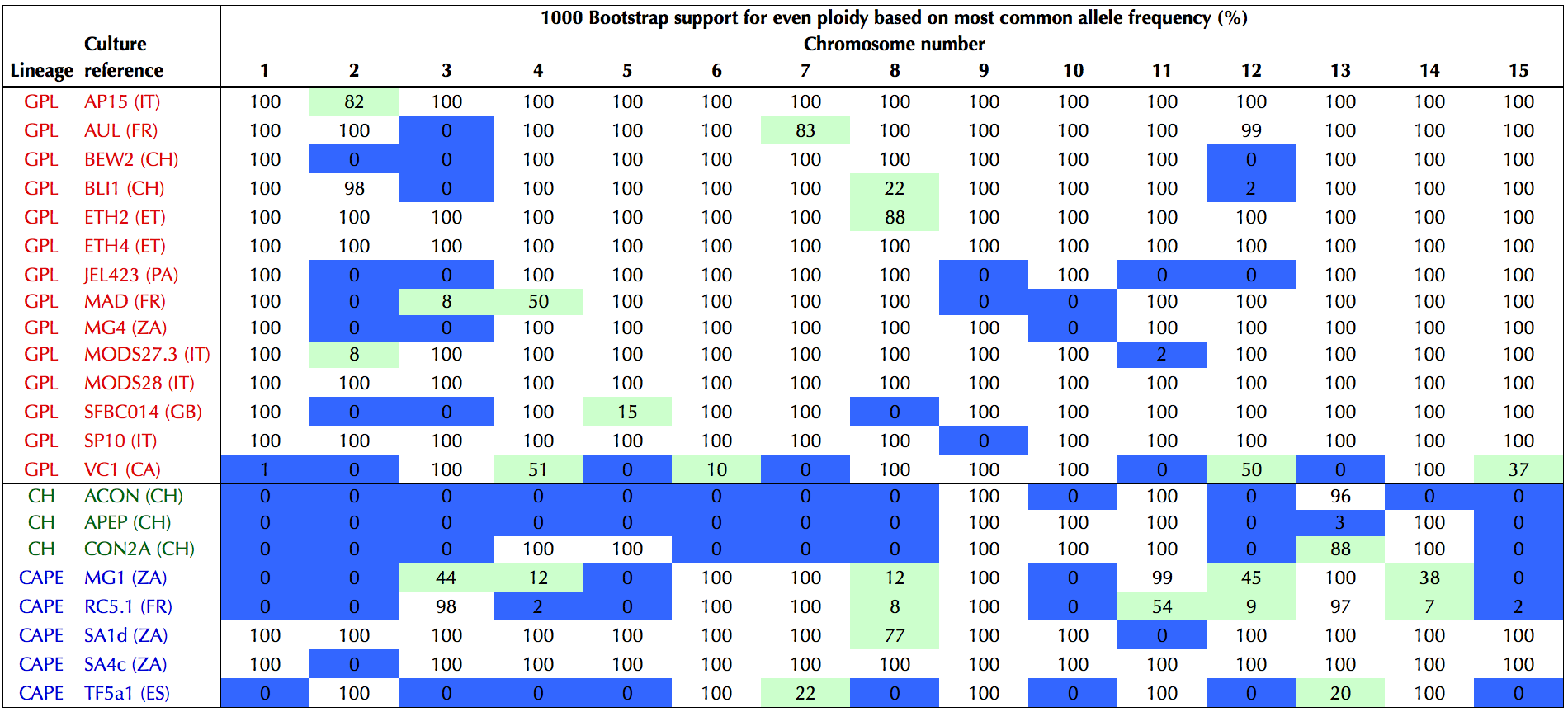

Supplement: Table S2 — The two most common allele frequencies over each base of each chromosome were determined by percent of read agreement with the reference base. Using 1000 Bootstrap replicates of these values, we recorded how often 47–53% reads agreeing with an allele predominated over 30–36% or 63–69% reads agreeing with an allele. Shown in white are chromosomes with >95% of replicates showing a predominantly bi-allele signature (even-ploidies). Chromosomes with <5% bootstrap support for an even number of chromosomes therefore had a high support for unbalanced allele frequencies (odd-ploidies), and shown in blue. Chromosomes not fulfilling these criteria are shown in green and considered ambiguous. (PNG) [file pgen.1003703.s021.png]

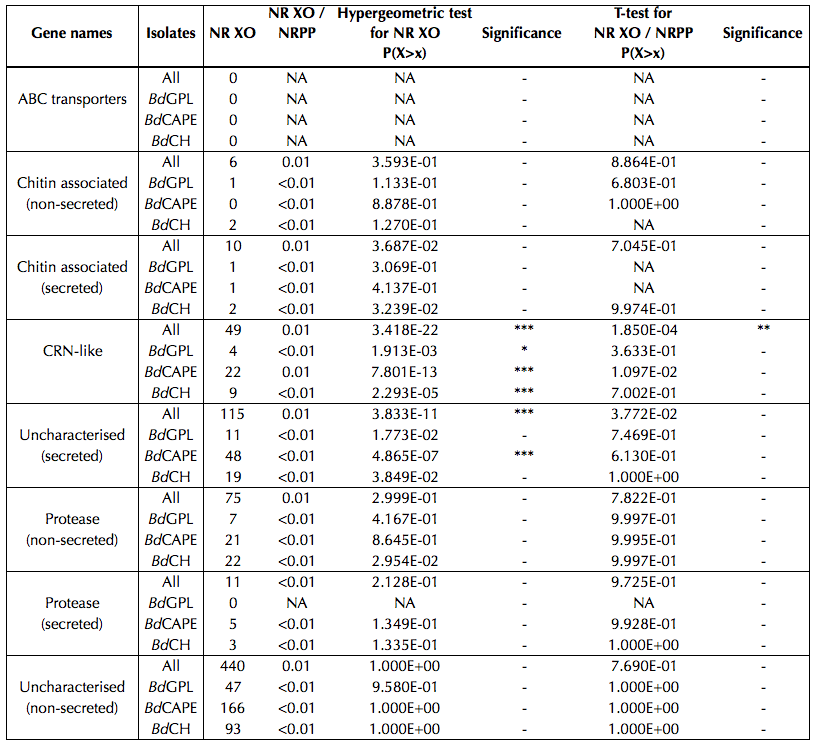

Supplement: Table S3 — Genes were tested for enrichment in non-redundant (NR; at unique loci) crossovers (XO) and NR XO/NR phased position (NRPP) compared to the values for all genes using Hypergeometric tests and t-tests respectively. For t-tests, all genes with <2 NRPP (the minimum required for a crossover) were excluded. Although both CRN-like and uncharacterised (secreted) were enriched for crossovers at unique loci (non-redundant), only CRN-like (between lineages) were enriched for XO/NRPP. (PNG) [file pgen.1003703.s022.png]

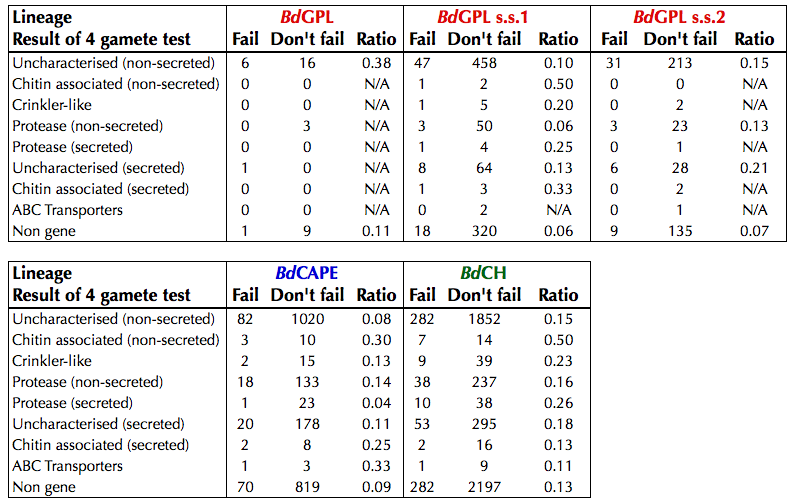

Supplement: Table S4 — Haplotypes over coding sequence that failed the four-gamete test were predominantly from coding-regions. Haplotypes overlapping a number of genes were included in the counts for each gene (385 extra counts to total number of haplotypes). After accounting for these extra counts, an additional 1,162 haplotypes were still found to come from coding regions compared with those from intergenic or intron regions. However, no gene group had a clear enrichment for haplotypes that failed the four-gamete test. BdGPL subset (s.s.) 1 consisted of isolates VC1, AP15 and JEL423. Subset 2 consisted of subset 1, ETH4 and MODS27. (PNG) [file pgen.1003703.s023.png]

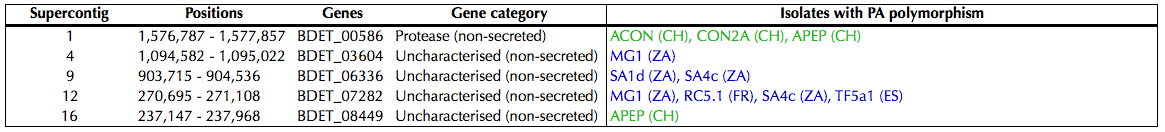

Supplement: Table S5 — Only five presence absence (PA) polymorphisms relative to BdGPL JEL423 were identified amongst BdCAPE and BdCH isolates, whilst none were identified amongst BdGPL isolates. (PNG) [file pgen.1003703.s024.png]

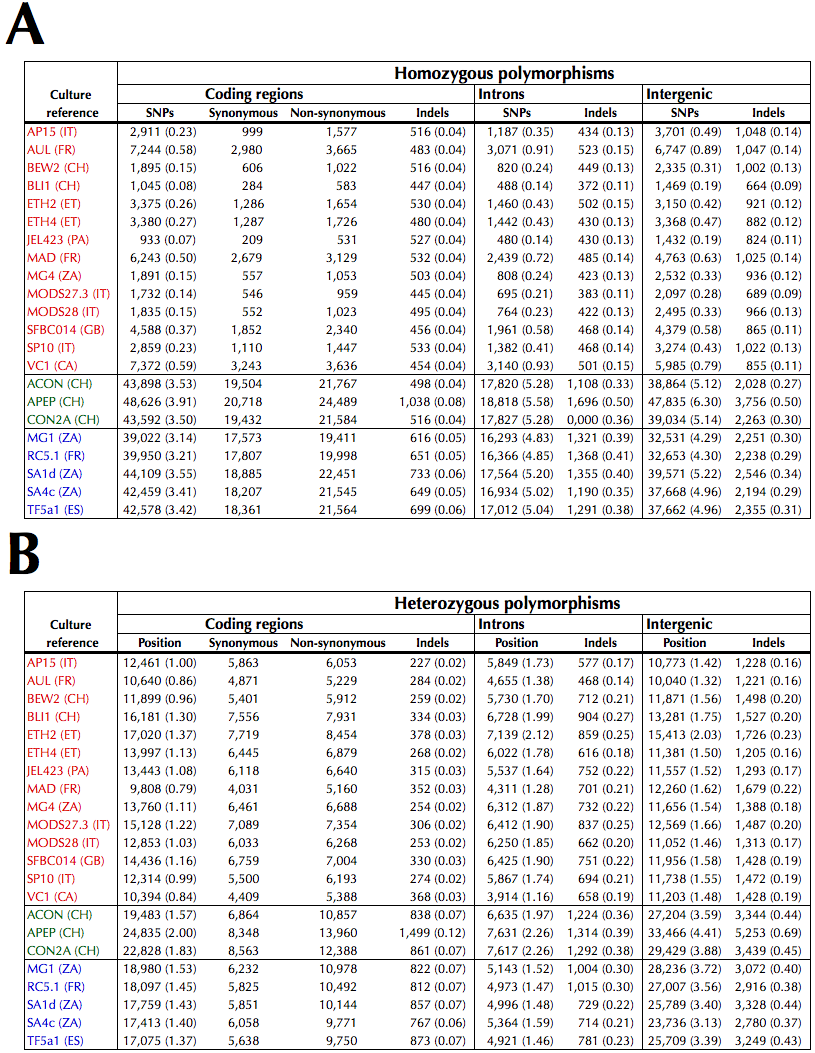

Supplement: Table S6 — Homozygous (A) and bi-allelic heterozygous (B) polymorphisms were found in the coding and non-coding regions of the Bd nuclear genomes. The total numbers of each variant-type are followed by their numbers per kilobase of genomic region in parentheses. For heterozygous positions, the affect on the transcript (synonymous/non-synonymous) was determined using the alternative allele. Where two alternative alleles to the reference sequence were found (infrequently), the first present within the VCF was chosen. With the exception of the reference strain Bd JEL423, the ratios of non-synonymous to synonymous changes were between 1.12–2.00 and 1.22–2.13 for homozygous and heterozygous positions respectively. (PNG) [file pgen.1003703.s025.png]

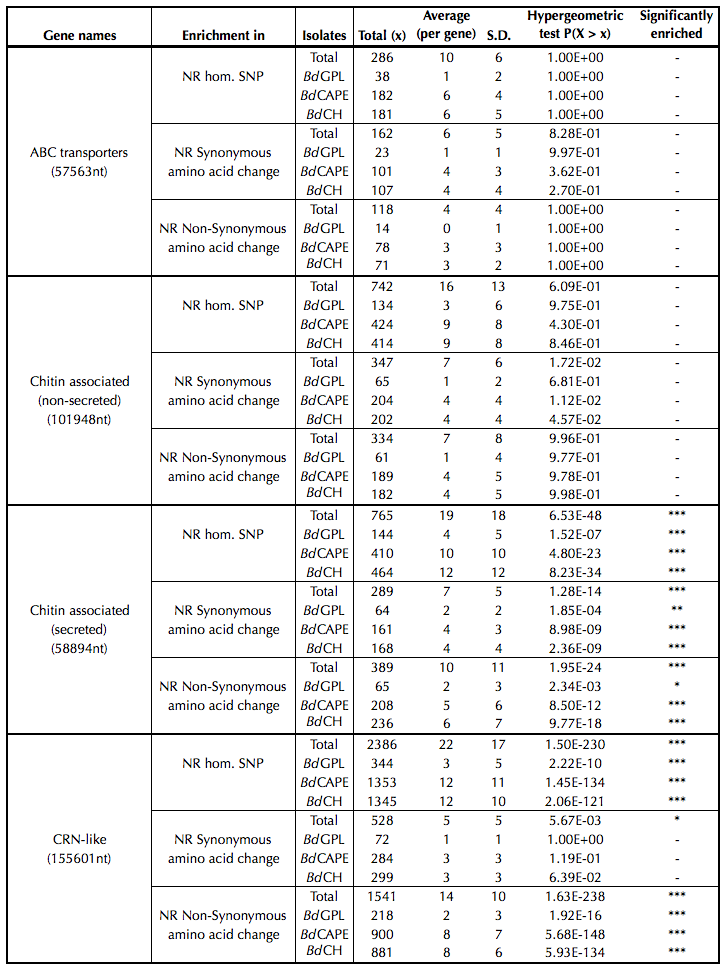

Supplement: Table S7 — ABC transporters, Chitin associated genes and CRN-like genes were tested for enrichment in homozygous SNPs. The total number, average and standard deviation of non-redundant homozygous SNPs for each gene family were calculated for all isolates, and lineage specific isolates. A Hypergeometric test was used to identify significant enrichment for variants where P<0.01 (*), P<0.001 (**) and P<0.0001 (***). (PNG) [file pgen.1003703.s026.png]

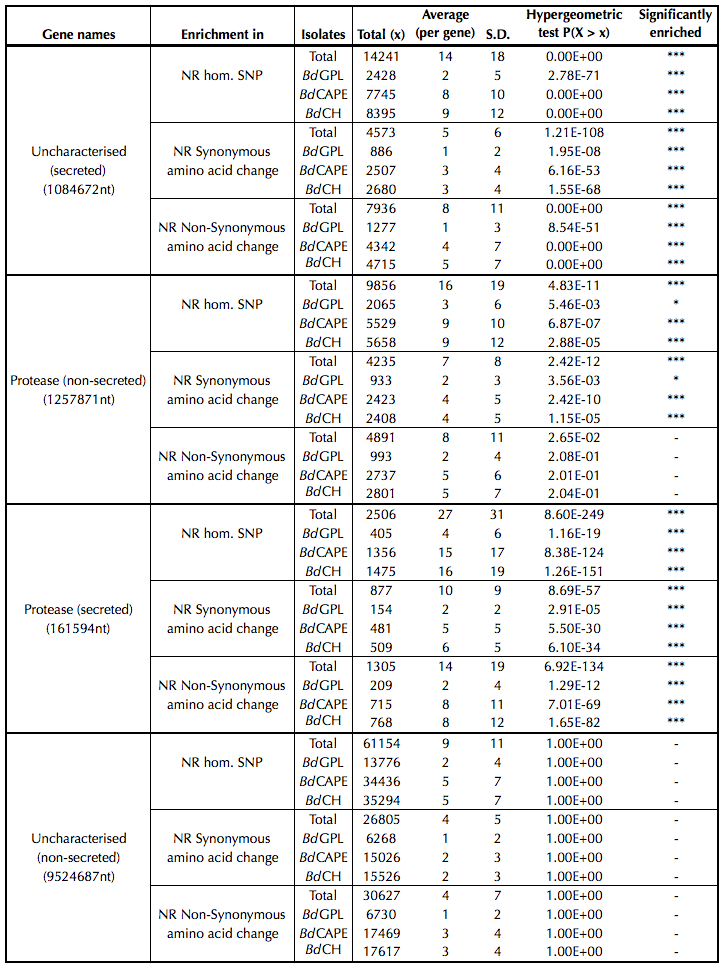

Supplement: Table S8 — Proteases, uncharacterized secreted genes and uncharacterized genes were tested for enrichment in homozygous SNPs. The total number, average and standard deviation of non-redundant homozygous SNPs for each gene family were calculated for all isolates, and lineage specific isolates. A Hypergeometric test was used to identify significant enrichment for variants where P<0.01 (*), P<0.001 (**) and P<0.0001 (***). (PNG) [file pgen.1003703.s027.png]

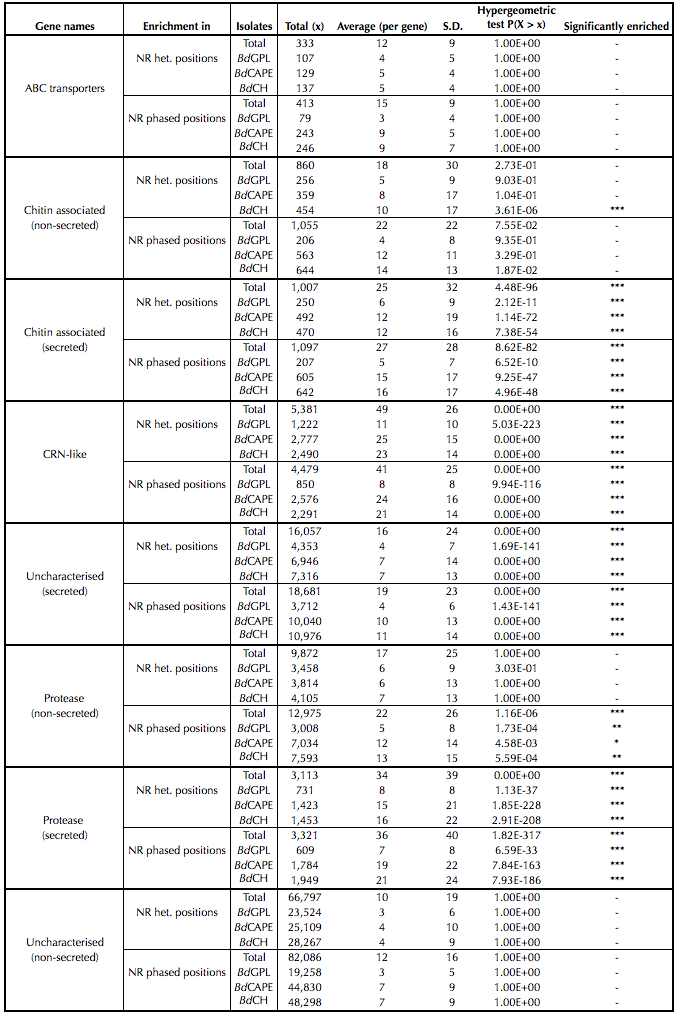

Supplement: Table S9 — Secreted and CRN-like genes are significantly enriched for heterozygous positions at unique loci. The total number, average and standard deviation of non-redundant heterozygous and phased positions for each gene family were calculated for all isolates and lineage specific isolates. A Hypergeometric test was used to identify significant enrichment for heterozygosity where P<0.01 (*), P<0.001 (**) and P<0.0001 (***). (PNG) [file pgen.1003703.s028.png]

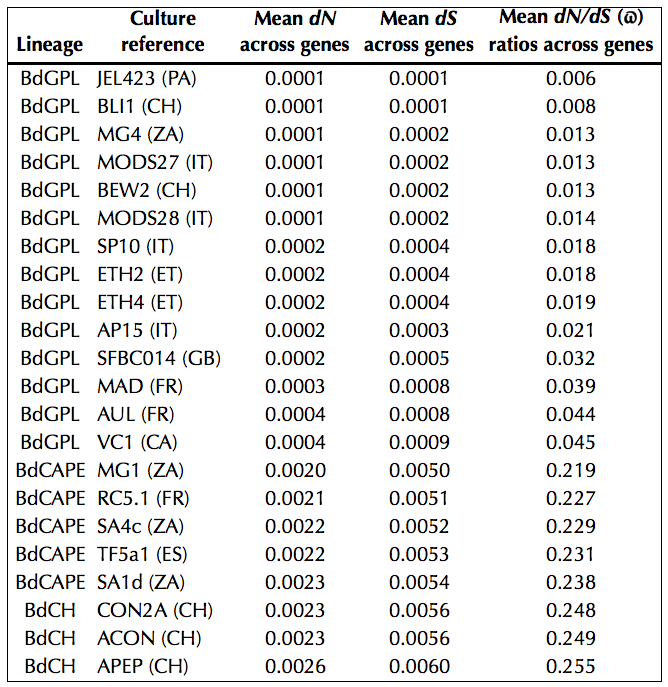

Supplement: Table S10 — The average rates of synonymous substitution (dS), non-synonymous substitution (dN) and omega (dN/dS = ω) for every gene in every isolate. (PNG) [file pgen.1003703.s029.png]

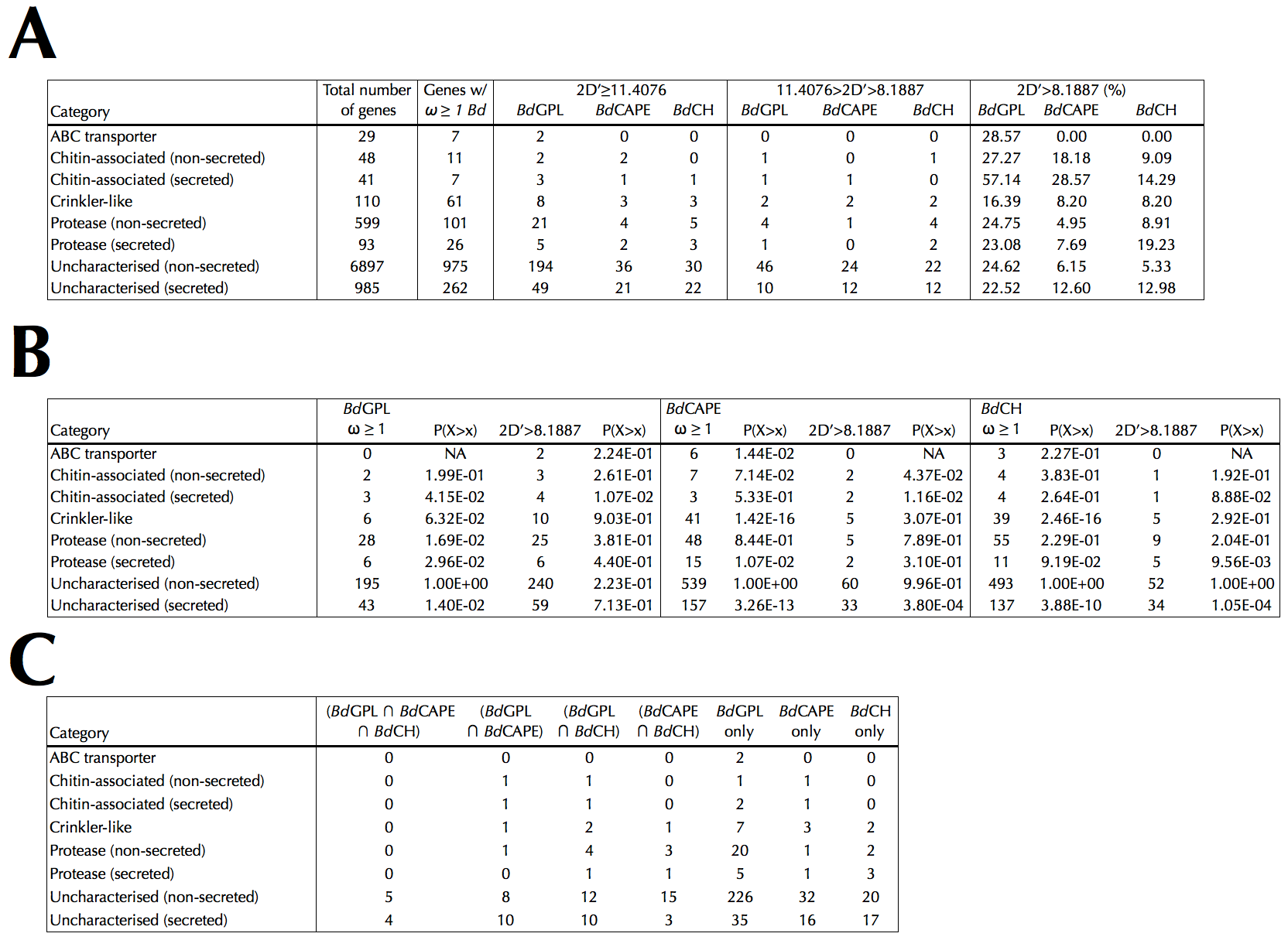

Supplement: Table S11 — Number and category of genes with ω≥1 (1,450 in total) that also were found to have undergone positive selection using the Branch Site Models in codeml. (A) The total numbers of genes, the numbers of genes with ω≥1 among all isolates, and how many of those genes had 2D′≥11.4076 (1% significance after Bonferroni correction) and 11.4076>2D′>8.1887 (5% significance after Bonferroni correction). The final column shows the percent of genes with 2D′>8.1887 from those with ω≥1. (B) For each lineage, the numbers of genes with ω≥1 and those that also had 2D′>8.1887. Following both of these columns are the results from a hypergeometric test for enrichment. For the genes with ω≥1, the test is for enrichment from the entire set of genes, whilst for the genes with 2D′>8.1887, the test is for enrichment from just the genes with ω≥1. (C) Overlap of genes with 2D′>8.1887. (PNG) [file pgen.1003703.s030.png]
