# Supplementary material for: Chromosomal Copy Number Variation, Selection and Uneven Rates of Recombination Reveal Cryptic Genome Diversity Linked to Pathogenicity
Source: PLoS Genet. 2013 Aug 15;9(8):e1003703. doi: 10.1371/journal.pgen.1003703 (PMC3744429; doi:10.1371/journal.pgen.1003703)
Supplement: Text S1 — Supplemental Materials and Methods. Full details of methods and analysis described in this manuscript. (DOC) [file pgen.1003703.s031.doc]

Supplemental Online Material for

Chromosomal copy number variation, selection and uneven rates of recombination reveal cryptic genome diversity linked to pathogenicity

Rhys A. Farrer^1,2‡^, Daniel A. Henk^1^, Trenton W. J. Garner^2^, Francois Balloux^3^, Douglas C. Woodhams^4^ and Matthew C. Fisher^1‡^

^1^The Dpt. Infectious Disease Epidemiology, Imperial College London, UK; ^2^Institute of Zoology, Zoological Society of London, UK; ^3^Dpt. of Genetics, Evolution and Environment, University College London, UK ^4^Dept. of Ecology & Evolutionary Biology, University of Colorado, USA

‡ To whom correspondence should be addressed. Email: r.farrer09@imperial.ac.uk; matthew.fisher@imperial.ac.uk

**This file includes:**

Text S1

References

Table of contents

Library preparation and sequencing 3

*In vitro* divergence of independent replicate lines of *Bd*CH 3

Optimization of alignments and SNP calling parameters 4

Phylogenetic analysis of nuclear genomes 6

Chromosome Copy Number Variation (CCNV) 7

Identifying gene groups and names 9

Recombination and hybridization amongst *Bd* isolates 10

Patterns of mutation and selection 15

References 18

Summary of results

Our analyses show threefold processes that contribute to the generation of *de novo* diversity in the emergent amphibian pathogen, *Batrachochytrium dendrobatidis* *(Bd*). Firstly, we show that the majority of wild isolates manifest lineage-specific biases in ploidy, with widespread chromosomal copy number variation (CCNV) seen in most isolates, and occurring over short timescales (< 40 generations). Secondly, we show that cryptic recombination occurs within all lineages of *Bd*, leading to significant regions of the genome being in linkage equilibrium. Thirdly, we show that recombination is not evenly distributed across the genome, and is associated with classes of genes of known importance for virulence in other pathosystems. Finally, we show that these classes of genes are under directional selection, and that this has predominantly targeted the globally-emerging virulent lineage *Bd*GPL

**Library preparation and sequencing**

Twenty-two isolates that had been collected from nine countries and four continents were chosen for sequencing (Table 1). Paired-end Libraries were constructed according to the protocols provided by Illumina sequencing (Truseq kit). DNA was sheared into 150-500bp fragments using a Covaris S2 sonicator. After end-repair and size selection using magnetic beads, barcoded adapters (Illumina 12plex tags) were ligated to the fragments and PCR amplified using 10 cycles. The final libraries were quality-controlled on a bioanalyzer and quantified with qPCR. Two pools of Libraries were used, and sequenced on 2 lanes of an Illumina HiSeq 2000 machine. The output read length was 100bp. Sequence data was processed using RTA version 1.12.4.2, with default filter and quality settings. Sequence files were generated with CASAVA 1.7. Fastq Conversions were performed with a custom script.

The genome sequence and feature file for the chytrid fungus *Batrachochytrium dendrobatidis* (*Bd*) strain JEL423 was downloaded from http://www.broadinstitute.org/ (GenBank project accession number AATT00000000). The feature file for JEL423 had all but the longest splice variants removed for each gene leaving 8794/8819 genes. We aligned all of our reads to the genome sequence using Burrows-Wheeler Aligner (BWA) v0.5.9 [1] with default parameters and converted to Samtools mpileup format using SAMtools v.0.1.18 [2].

***In vitro* divergence of independent replicate lines of *Bd*CH**

An isolate of *B. dendrobatidis* from a Swiss *Alytes obstetricans* (isolate 0739) was subcultured into control (ACON) and peptide-treated (APEP) culture flasks containing 10 ml 1% tryptone media supplemented with 1% penicillin-streptomycin (Sigma) to reduce the risk of bacterial contamination. Cultures were incubated at 18°C and passaged every 4-5 d by scraping the side of the flask and transferring 1 ml into 9 ml fresh media. Peptide-treatment included addition to the media of 80 µg ml^-1^ skin defense peptides collected from *Pelophylax esculentus* (n=15 combined) according to Daum *et al.* (2012) [3]. This was equivalent to the IC_50_, or the concentration at which growth of *Bd* was inhibited by 50%. These three isolates were included in the panel of 22 for whole-genome sequencing.

Phenotypic changes following selection were assessed on the above sequenced isolates and additional replicates from treatment and control conditions. Growth of the isolates in five replicate wells in media containing 100 µg ml^-1^ skin defense peptides in water was compared to growth of the isolate with water added (positive control) or with water added and heat killed (negative control) according to standard protocols for determining growth inhibition [3].

After 40 passages with or without amphibian skin defence peptides added to media, *Bd* growth was measured to determine the effect of selection on resistance to peptides. Mean growth inhibition (% ± SE) of *Bd* given 100 µg ml^-1^ peptide was: 14.23 ± 11.48 for *Bd*CH APEP, 59.44 ± 14.50 for *Bd*CH ACON, and 55.74 ± 5.98 for *Bd*GPL. When replicate cultures were compared, the average growth inhibition of *Bd*CH after selection for peptide resistance was 11.43 ± 3.02. The value was significantly reduced compared to controls at 48.81 ± 6.72 (independent t-test, t=-3.980, df=3, p=0.028).

**Optimization of alignments and SNP calling parameters**

We resequenced the genome for isolate JEL423 to act as a control for sequencing, alignment and SNP calling. To test for ability to accurately call polymorphisms from the alignments, we aligned the reads from JEL423 to two modified reference *Bd* JEL423 genomes (1nt/1Kb and 1nt/100nt within the coding regions; CDS) reflecting the different levels of sequence divergence found within and between different lineages of *Bd* (Fig. S1) using the comparison of false discovery rate (FDR) method [4]. We next tested the SNP-callers: Binomial SNP-Caller from Pileup (BiSCaP) v0.1 and v0.11 using a minimum depth of 4 reads [4] and the SAMtools mpileup command piped to Bcftools v0.1.17-dev and filtered using vcfutils.pl with default parameters [2]. We tested each method with variable minimum required read-depths and compared results after filtering SNPs called from alignment to a non-modified reference.

In order to assess heterozygous variants, we randomly chose and modified 1nt/Kb and 1nt/100nt within the CDS using the “HET” setting of IRMS.pl, which first generates a duplicate (homologous) genome. We then simulated single-end reads from these modified sequences to the same depth as the ‘real’ data using simLibrary and simNGS (http://www.ebi.ac.uk/goldman-srv/simNGS/) using the default runfile (s_3_4x), which describes how “noise and cluster intensities are distributed in a real run of an Illumina machine”, and aligned those reads to the non-modified reference genomes. Finally, the same SNP-callers used for the FDR of homozygous SNPs were used to call heterozygous bases. To compare the heterozygous-base calls with those achievable from our previous SOLiD datasets, we applied the same method using simulated 10.17X deep 30mers.

We achieved a variety of success rates (true positives, TP > false positives, FP) at calling the polymorphisms depending on the SNP-calling method and its settings. SNP-calling from our previous *Bd* JEL423 SOLiD sequences [5] achieved 40-50% TP for both simulated divergences and both versions of BiSCaP, whilst the Illumina data presented here achieved >95% TP using either BiSCaP versions 0.1 or v0.11 or SAM/BCFTools for both simulated divergences. Simulated reads to the depth and read-length of the previous SOLiD sequences were used to assess its ability at called heterozygous positions. BiSCaP v0.11 achieved 40-60% TP and 1-6% FP for both simulated divergences. Aligning simulated reads to the depth and read-length of the new Illumina sequences achieved 81.38% TP and 0.29% FP for the greater divergence and 92.17% TP and 0.23% FP for the lesser divergence. Filtering SNPs called from a non-modified reference revealed that many of the FP SNPs identified by either BiSCaP or SAM/BCFTools were independently found without the simulated divergence, and are therefore likely to reflect either real errors in the reference genome or discrepancies between the two *Bd* JEL423 isolates. Using BiSCaP v0.11 with a minimum of four reads (after taking into account polymorphic positions identified using a non-modified reference) called the optimum numbers of both TP and FP and was therefore used to call homozygous SNPs and bi-allelic heterozygous positions across each of the 22 genomes.

Across the 22 nuclear genomes, we identified 218Kb non-redundant (at unique loci) SNPs (average 9.3SNPs/Kb) and 279Kb non-redundant bi-allelic heterozygous positions (average 11.9hets/Kb) (Table S1A). To check our variants were not a function of difficulties in mapping reads accurately, we checked the number of best hits in the alignment (SAM file) for every read over every variant. For this analysis, we ignored entries missing the optional field specifying the number of best hits (XO tag). Reads with exactly 1 best hit were considered uniquely mapped, whilst those with zero or >1 were considered as non-unique (Table S1B). We found that on average >85% of our reads were uniquely mapped across the genomes, with homozygous SNPs having >90% uniquely mapped reads and bi-allelic heterozygous positions having >82% uniquely mapped reads. However, isolates belonging to the two divergent lineages had less uniquely mapped reads over bi-allelic heterozygous (averaging 68%, compared with 91% in *Bd*GPL). This reduction in uniquely mapped reads may result from structural variations such as gene presence/absence polymorphisms. Therefore, false positive rates over heterozygous positions may be greater than that found with our FDR experiment. However, phased positions comprising both homozygous SNPs and heterozygous positions had between 86-94% uniquely mapped reads in any given isolate, while the average across the genomes were between 79-87% uniquely mapped reads, suggesting this issue did not propagate into poorly resolved haplotypes.

Of the total number of polymorphisms identified, 424,631 were at unique loci, and 275,009 were called as either reference or polymorphic in all 22 isolates (entirely covered and verified in all; ECVA). Extensive overlap of ECVA polymorphisms was found by calculating for every two pairwise isolate ((sample1 ∩ sample2) / ((sample1 + sample2) / 2)) x 100. We found the greatest levels of variation amongst homozygous SNPs within *Bd*GPL (between 17% and 95%) compared with *Bd*CAPE (93-96%) or *Bd*CH (92-97%) (Fig. S2A). *Bd*GPL also had the greatest variation amongst heterozygous positions (between 32% and 75%) compared with *Bd*CAPE (44-55%) or *Bd*CH (34-55%) (Fig. S2B).

**Phylogenetic analysis of the nuclear genomes**

Entirely covered and verified in all (ECVA) polymorphisms comprising 275,009 positions were extracted from each of the isolates and concatenated into FASTA files for phylogenetic analysis. Bi-allelic loci were represented in the FASTA file by the corresponding ambiguity codes. FASTA files were converted into Nexus files and a tree constructed using the Un-weighted Pair Group Method with Arithmetic Mean (UPGMA) algorithm in PAUP and visualised using Figtree [6] (Fig. S3A). In addition, we extracted 36,309 positions fulfilling the same criteria for the newly sequenced isolates and the previous 20 isolates sequenced using the SOLiD platform [5] comprising 42 isolates including *Bd*GPL JEL423 (PA) and *Bd*CAPE TF5a1 (ES) sequenced by both platforms (Fig. S3B). Finally, we performed the same analysis using just the homozygous positions (reference or SNP in all) comprising 218,269 positions across the Illumina sequenced isolates and 8,457 positions across the Illumina and SOLiD sequenced isolates (Figs. S3C and S3D respectively). All four trees demonstrated three divergent lineages previously identified [5]. Amongst *Bd*GPL, JEL423 for both platforms came together in both trees. Among the more distant lineages, the Illumina sequenced *Bd*CAPE isolates clustered separately from the SOLiD sequenced *Bd*CAPE isolates, including in the replicate isolate TF5a1, likely demonstrating sequencing errors and lack of depth in the previous SOLiD datasets.

**Chromosome Copy Number Variation (CCNV)**

We predicted ploidy levels across the genomes using depth of coverage and percent of reads specifying two most frequent alleles at a loci. Depth of read coverage across the genome was visualised using non-overlapping sliding windows of length 1414nt (half the mean length of the *Bd* transcripts) and 10Kb. This analysis identified abnormal numbers of chromosomes (CCNV) in every lineage of *Bd* and across at least nine separate chromosomes (Figs. 1 and S4). To quantify these changes, we performed T-tests on the mean depths across the largest supercontig (supercontig 1) against each subsequent supercontig for each isolate (Fig. S5). Using a stringent cut-off of p<5^-10^, we found a significant increase in copy number for 36 of the supercontigs and a decrease in 25 against the base ploidy level. Chromosome 14 had a large peak of depth in all isolates (Fig. 1), which occurred over a long stretch of rDNA sequence identified using the nr BLAST database. We therefore excluded Chr14 from the following analysis of CCNV.

To determine the ploidy of the largest chromosomes (1-13,15-16) of the 23 isolates (97% of the total genome length), we calculated the percent of reads specifying the two most frequent alleles (Fig. S7) for each chromosome in each isolate separately with a minimum depth cut-off of 4 reads for both alleles. By binning the percent of reads aligned over each base in the genome agreeing with a nucleotide between 47-53% (expected even ploidy/bi-allelic) and 30-36% and 63-69% (expected odd ploidy/tri-allelic). We found that supercontigs with significant increase or decrease in depth also corresponded to changes in allele-frequencies (i.e. from high peaks between 30-36 and 63-69 to a high peak between 47-53) and were consistent within a genome. To check that these values could be found given different subsets of each chromosome in each isolate, we calculated 1000 bootstraps for either predominance of bi-allelic or tri-allelic peaks (Table S2). Using a 5% cut-off (5%<x<95%) we found 305/330 largest 15 chromosomes gave confident odd or even allelic peaks. Chromosome 1 in each isolate had bootstrap support for having even or odd alleles, which could therefore used to predict the base-line ploidy (along with T-tests for sequence depth) for that isolate.

Specifically, should supercontig 1 for an isolate have predominantly tri-allelic peaks, then all chromosomes with no significant change in mean depth and also predominantly tri-allelic peaks were considered triploid. Conversely, should supercontig 1 have predominantly bi-allelic peaks, then all supercontigs with no significant change in mean depth and also predominantly bi-allelic peaks were considered diploid. When a supercontig had different allele peaks (bi- instead of tri- or visa-versa) to that of supercontig 1, and a significant change in depth, it was considered a ploidy below or above that of supercontig 1. When mean depth and allelic-peaks did not match, depth alone could be used to predict the ploidy.

Two *Bd*GPL isolates (JEL423 and MODS27) were found to have supercontig 1 containing predominantly bi-allelic peak, yet other supercontigs with significant decreases in depth (p-values of T-tests). Over these supercontigs there were no decrease in heterozygotes called and tri-allelic peaks were also identified. For these isolates, supercontig 1 and all other similar supercontigs (depths and allele-frequencies) were therefore predicted to be tetraploid, and the chromosome with lower depth was considered trisomic. Two *Bd*CAPE isolates SA1d (ZA) (shown in Fig. S6) and SA4c (ZA) were also predicted as having predominantly tetraploid genomes by this criteria.

Using depth and allele-frequencies we calculated the ploidy for 345 chromosomes. 287 (>83%) had mean depths and consistent corresponding bi- or tri-allelic peaks across the isolates. We identified 52 chromosomes that had changed ploidy, 37 of which (71%) had both depths and corresponding peaks. 14/16 *Bd*GPL isolates were predominantly diploid with as many as 3 chromosomes in greater copy number. All of the *Bd*CAPE and *Bd*CH isolates were predominantly polyploid with evidence of chromosome loss or gain in every isolate.

**Identifying gene groups and names**

All 8819 *Bd* JEL423 transcripts were searched for secretion signals, protease domains and carbohydrate binding protein domains using SignalP3.0 [7], Merops [8] and Procarb604 v1 [9] respectively. Gene annotation (downloaded) and blastx searching was performed against the non-redundant BLAST database with Blast2Go [10] using a 1e-05 e-value cut-off, which found hits to >64% of the transcripts. BLAST annotations were also screened for the key terms ‘crinkler’, ‘peptidase’, ‘protease’, ‘metalloproteinase’, ‘chitin’, ‘abc transporter’ and ‘abc multidrug transporter’ to further identify candidate gene groups. In total we identified 599 non-secreted proteases, 93 secreted proteases, 41 secreted chitin-associated genes, 48 non-secreted chitin-associated genes, 110 Crinkler (CRN-like), 985 non-categorised secreted genes, 29 ABC transporters, and the remaining 6897 (78%) uncharacterized genes. Many genes were identified in the same functional group using the different methods, and no gene was identified in more than one group.

In addition to grouping genes using naming and searching protease, carbohydrate and secretion peptide databases, we grouped genes entirely based on sequence. First, all protein sequences were Blastp searched against themselves, again with a 1e-05 e-value cut-off. Gene tribes were identified using the MCL software [11] with settings stream-mirror, stream-neg-log10 and I=2 (recommended settings). Where genes had multiple transcripts, only the longest was considered. From these 2 methods we identified 621 clusters of genes with 2 or more transcripts that matched a gene from the NCBI Sequence database, encompassing 3132 genes (36% of total transcripts).

The largest gene tribe (Tribe 1) included 564 genes encompassing genes from all 8 categories of genes, including a large number of CRN-like, Proteases and ABC Transporter genes (from largest to smallest): 75/110 (68%) CRN-like genes, 14/29 (48%) ABC-transporters, 39/93 (42%) proteases (secreted), 15/48 (31%) chitin-associated (non-secreted), 7/41 (17%) chitin-associated (secreted), 69/599 (12%) proteases (non-secreted), 33/985 (3%) uncharacterised (secreted) genes and the rest (305) uncharacterized (non-secreted). Although this prevented using this grouping method along side the method based on blast searches, it demonstrates that some shared homology exist between many of these genes that are putatively involved in virulence, despite having potentially very different roles and undergoing different selection pressures.

**Recombination and hybridization amongst *Bd* isolates**

We identified >10X the number of ‘covered in all’ polymorphic loci from our previous round of SOLiD sequencing [5] due at least in part to increased sequence depth and read-length. To check if the same pattern of patchy heterozygosity could be found, we made new plots of 'SNPs minus heterozygous positions' across the genome using a 10Kb non-overlapping window. We identified the same pattern across the genome including an absence of heterozygosity across the majority of chromosome 2 in the *Bd*GPL (Fig. S7). The mechanism generating this uneven distribution remains unknown however may reflect a shared ancient meiotic segregation that unifies all isolates of *Bd*GPL, and is unique to *Bd*GPL.

In order to further investigate recombination, we phased the bi-allelic heterozygous positions (in Variant Call Format) identified by BiSCaP v0.11 according to overlapping reads. Firstly, the corresponding SAMTools sorted BAM file to each isolates VCF was searched for reads that mapped to the genome without any indels, and also covered two or more heterozygous positions. The phase for each of the overlapping heterozygous positions was then calculated. To ensure well supported haplotypes corresponding to only two alleles per loci, any heterozygotes with less than four overlapping reads over each of the bases, and those with <90% agreeing with a single phase (such as heterozygous positions in different phases or different bases, either of which may be due to polyploidy or error) were filtered from all analysis (see Section: Chromosome Copy Number Variation). Phased heterozygotes were given unique numbered identifiers and placed in an ordered VCF for each isolate, which could be used to distinguish which positions were in phase with others. Homozygous SNPs within phased groups were also combined into that group. A diagram of the phasing is shown in Fig S8.

Across the 22 isolates, 71% of the identified heterozygous positions were eligible for pre-filtered phasing whereby each had at least four reads overlapping that and at least one other heterozygous locus and >90% of the reads specified the two nucleotides of the heterozygote (thereby further filtering any potential tri-allelic heterozygous position wrongly called as bi-allelic). Of these 604,895 positions, 487,605 (80.61%) of the overlapping reads inferred a single phase (using the >90% cut-off; Fig. S9). In total, 412,883 (84.67%) of the eligible heterozygous positions inferred a single bi-allelic phase without any exceptions (100% reads agreeing with phase). By lineage, *Bd*CAPE and *Bd*CH isolates had a lower proportion of resolved bi-allelic phased positions (70 and 74% respectively) compared with 93% in *Bd*GPL isolates, which may have resulted from greater numbers of tri-allelic loci in trisomic chromosomes. A separate possibility is due to the greater divergence of *Bd*CAPE and *Bd*CH isolates to the reference genome, and the subsequent issues with alignments. Although the corresponding tested divergence had no increased FDR for SNP-calls (see Section: Optimization of alignments and SNP calling parameters), other issues such as gene-duplications followed by mutation may increase the false positive rate.

In order to extend haplotypes, we included all homozygous SNPs within a given phased group and created new phase groups consisting of consecutive homozygous SNPs. Finally, we identified consecutive "SNP, Bi-allelic HET, SNP", which can therefore also be considered either a new separate phase group or joined to the previous phase group if no other un-phased variant occurs between them. As a proportion of the total homozygous SNPs and bi-allelic heterozygous positions called across each of the 22 isolates, 72.48% were phased using these methods (Table S1).

To compare linkage between isolates, we first performed pair-wise comparisons of haplotypes found between each pair of isolates (Fig. S10). Between *Bd*GPL isolates, >99.8% of the ‘phased in both’ heterozygous positions were found in the same phase. Within *Bd*CAPE and *Bd*CH isolates, >98.9% and >99% were within the same phase respectively. Even pairwise comparisons for isolates belonging to separate lineages had >92% positions in the same phase, thereby supporting the hypothesis that the population of *Bd* is predominantly clonal and non-recombining. However, a total of 4,974 crossovers were also identified, 2,007 of which were at unique locations in the genome. Every pairwise comparison of phased positions (except between *Bd*GPL isolates MAD (FR) and AUL (FR)) revealed at least one crossover (Fig. S11), even when comparing isolates that likely share a very recent common ancestor.

Crossovers were most prevalent amongst *Bd*CAPE and *Bd*CH isolates (between 0.6 and 1.1% of phased positions) compared with 0-0.2% in *Bd*GPL. This was a surprising finding given that the three *Bd*CH isolates were separated by only 40 passages in the lab (see Section: *In vitro* Divergence of Independent Replicate Lines of *Bd*CH) and given our previous results demonstrating unique hallmarks of recombination only in the *Bd*GPL [5]. While it is unclear how the rate of recombination within a given lineage may have changed since its divergence with other lineages, a number of possibilities could explain these differences. Firstly, *Bd*CAPE and *Bd*CH are able to recombine within their lineage (either by selfing or out-crossing) and *Bd*GPL (possibly following an ancestral meiotic recombination event) has had a principally clonal expansion with reduced opportunities for meiosis. Alternatively, *Bd*GPL has been undergoing non-meiotic recombination such as gene-conversion but not selfing or out-crossing (thereby showing patchy heterozygosity, and a reduced level of crossovers). A small proportion of the identified crossovers could have arisen from two separate mutations *de novo* at that locus. However, the consistency found between isolates in a given lineage suggests this is not having a large effect, if any. As a separate speculative comment, it is worth noting the association between higher levels of recombination within *Bd*CAPE and *Bd*CH and their hypovirulence compared with *Bd*GPL.

To further look for evidence of recombination, a ’phased in all’ file (PIA) was constructed from overlapping phase groups identified across all VCFs within a given lineage. Phased groups that were <10nt long were filtered out to avoid short repeats or closely associated heterozygotes resulting from poorly resolved indels as reported here [12]. Next, overlying variants (homozygous SNPs and phased heterozygous positions) were extracted from each VCF. Isolates lacking any variant at those positions had their corresponding SAMTools pileup lines extracted, and reference-base calling was performed using the same default settings of BiSCaP [4] as used for the variants. All positions that were not called as a reference-base were excluded from further analysis. Positions lacking any variant, but were called as a reference base were included. Next, all fixed loci in a given lineage were filtered out. Haplotypes with less than two remaining loci or any that have been reduced to <10nt were also filtered. The remaining haplotypes consisting of those polymorphic loci were constructed (2 haplotypes per isolate as phased groups were extracted from bi-allelic regions). In this way, a total of 2,275 haplotypes for *Bd*CAPE and 5,215 haplotypes for *Bd*CH were extracted ranging in length from 10nt up to 21.744Kb, with similar lengths found in both lineages (Fig. S12 and Table 2). However, only 35 haplotypes for BdGPL fulfilled those criteria. To test *Bd*GPL with a greater number of haplotypes, and to check if the different numbers of isolates contained in each lineage affected the linkage analysis, we made two subsets from *Bd*GPL and reran the analysis. Subset 1 consisted of VC1, AP15 and JEL423 (919 haplotypes), and the 2^nd^ subset including subset 1 as well as ETH4 and MODS27 (438 haplotypes), which were chosen based on their representation of the total diversity in the UPGMA tree (Fig. S3).

From these haplotypes, we were then able to calculate the Index of association (I_A_) whereby linkage disequilibrium for a given set of haplotypes is suggested if VD > L (L_old_). We also calculated rBarD values and performed four-gamete tests between every combination of loci in a haplotype (fig. S9) to quantify the amount of recombination occurring within populations. In addition, we performed Weir’s [13] formulation of Wright’s Fixation Index (*F*_ST_) according to the equations given in Multilocus 1.3 [14].

Across the *Bd*GPL groups, >30% of phased positions were in significant disequilibrium compared with 16% and 11% for *Bd*CH and *Bd*CAPE respectively. RbarD values appeared to be more robust against sample size differences, and gave haplotypes from *Bd*GPL values of 0.79-0.82 compared with 0.58 and 0.61 for *Bd*CH and *Bd*CAPE. Finally, a smaller proportion of *Bd*GPL subset haplotypes failed the four-gamete test compared with *Bd*CAPE or *Bd*CH isolates. Each of these findings shows that recombination is occurring within each of lineages. However, the emergent *Bd*GPL is far more clonal than the other naturally occurring lineages. To check for genome-positional enrichment, the locations of each intra-lineage heterozygote, the percent of which were phased, and those that demonstrated a crossover were plotted across the genome using a non-overlapping 1.4Kb window (Fig. S13). The location of each haplotype, its corresponding rBarD value, those that failed the 4 gamete test, as well as the mean rBarD were also plotted (Fig. S13). Every chromosome in each of the three lineages contributed roughly equal numbers of all of these variables.

To look for evidence of hybridization, we counted the number of variable sites per locus across the haplotypes with an expectation that a hybrid lineage should have a greater number of variable sites at a given locus. Between 70-75% of *Bd*GPL subsets 1 and 2 loci consisted of 2 different bases compared with 75% in *Bd*CAPE and 72% in *Bd*CH (Table 2). Additionally, *Bd*GPL subsets had between 20-24% of loci consisting of 3 different bases compared to 24% in *Bd*CAPE and 25% in *Bd*CH. These values suggest that none of the three lineages stand out as an expected hybrid given the expectation for a greater percent of variable sites per locus. We next calculated the fixation index (*F*_ST_) for each pairwise lineage across window lengths of 1.4Kb and 10Kb (Fig. S14) using all homozygous (SNPS or reference) and bi-allelic heterozygous positions (and excluding all loci with neither in all). All three lineages are differentiated from one another across each chromosome, with only minor intra-chromosomal variation, such as the stretch of rDNA located at the start of chromosome 14. Both the *F*_ST_ and the number of different bases at haplotypes loci suggest that *Bd*GPL, *Bd*CAPE and *Bd*CH are not recombining with one another.

Finally, to study which genes were recombining; we ran Hypergeometric tests for any non-redundant crossover (NR XO) position for each lineage separately (intra-lineage) across each of the gene categories (see Section: Identifying gene groups and names). Surprisingly, all three lineages had significant crossover-enrichment for CRN-like genes, and *Bd*CAPE as well as inter-lineage counts had enrichment for uncharacterised (secreted), both of which might be implicated in *Bd* pathogenicity (Table S3, Figs. S15-S16). To check if this was simply a result of greater heterozygosity providing an increased proportion that was phased and therefore our ability to detect recombination, we normalised to the number of phased position from which XO could be called (NRXO/NRPP). Again we tested for significant enrichment using *t*-tests (Table S3), now finding that only CRN-like genes remained enriched amongst all *Bd* isolates (inter-lineage). Haplotypes that failed the four-gamete test were predominantly from coding-regions, but had no clear pattern of enrichment for any gene category (Table S4). It is unclear and remains to be seen whether these genes are recombining at a greater rate due to an unrelated process, or if they are actively involved in recombination (e.g. a recombination motif) between homologous sequences.

**Patterns of mutation and selection**

To first look for genes that are present in the reference sequence and absent in our panel of isolates (presence/absence polymorphism), we identified any genes with zero depth of coverage (no reads aligned to them). From this analysis we identified only 5 genes that were absent from our panel (Table S5). None of these genes were absent amongst BdGPL isolates. Three of these genes were uncharacterised (non-secreted) genes and were *Bd*CAPE specific. The remaining two were an uncharacterised (non-secreted) gene and a protease (non-secreted) gene that were *Bd*CH specific. Therefore, whilst high-levels of aneuploidy are occurring, it does not appear to be resulting in gene loss as might be expected.

To study the patterns of mutation across the nuclear genome, we categorized each of the variant-types by their location in the genome in terms of coding regions (CDS), introns and intergenic regions (Table S6). In every isolate we sequenced, every variant type was found in greater abundance per kilobase in the intergenic and intron regions compared with the CDS (with the exception of 0.01/Kb fewer heterozygous positions in the introns compared with the CDS for isolate MG1). Specifically, introns had 1.43-1.90X SNPs, 1-1.79X heterozygous positions, 3.01-8.72X homozygous indels and 3.14-10.62X heterozygous indels compared with the CDS for each of the isolates (per Kb). Intergenic regions had 1.25-2.52X SNPs, 1.33-2.47X heterozygous positions, 2.44-7.19X homozygous indels and 5.75-9.48X heterozygous indels than the CDS for each isolate (per Kb). We further grouped heterozygous and homozygous indels in each of the three regions, identifying the introns to have 4.37-6.73X more than the CDS, and the intergenic regions to have 4.12-6.79X more than the CDS (again, both per kb).

Next, we categorized each of the mutations within CDS regions as synonymous and non-synonymous mutations (Table S6). A total of 391Kb of homozygous SNPs were detected amongst the 22 isolates within the CDS, accounting for 169Kb synonymous changes and 197Kb non-synonymous changes. Similarly, heterozygous positions accounted for a greater number of non-synonymous changes (179Kb) than synonymous changes (138Kb). With the exception of the reference strain *Bd* JEL423, the ratios of synonymous to non-synonymous change were between 0.49-0.91 and 0.56-0.97 for homozygous and heterozygous positions respectively. These results are in contrast to the previous SOLiD sequencing [5], whereby greater rates of synonymous change were identified. Although shared covered positions revealed similar SNPs and therefore comparable phylogenetic trees (Figs. S3B and S3D), the greater depth of sequencing, longer 100nt paired-end reads (compared to ~10X deep single 30nt SOLiD reads), and improved SNP-calling (Fig S1) each enable more robust variants to be identified, especially over hyper-variable regions such as mutations that are in close proximity.

Each of the gene groups (see Section: Identifying gene groups and names) was tested for enrichment of homozygous SNPs (Table S7-8) and heterozygous positions (Table S9) using Hypergeometric tests. Gene groups that carried a secretion signal (proteases, chitin-binding and uncharacterized secreted) as well as CRN-like genes were significantly enriched for both homozygous and heterozygous polymorphisms relative to the whole set of genes. Predicted chitin-binding proteins that lacked a secretion peptide were not enriched for either type of polymorphism, and proteases were enriched only for synonymous amino acid changes. Conversely, CRN-like genes are only enriched for non-synonymous homozygous SNPs and not synonymous SNPs. This is further demonstrated by the ratio of 2.95-3.17:1 non-synonymous to synonymous SNPs at unique loci across all three lineages of *Bd*, compared with <1.76:1 for any other gene families tested. These findings are the first demonstration that gene families are evolving differentially in *Bd*, and that genes putatively interacting with the amphibian host as well as CRN-like genes (whose function are unknown, but see Section: Recombination and hybridization amongst *Bd* isolates) are undergoing increased diversifying selection compared with those that remain within the pathogen.

To further measure selection across the genome we measured the rates of synonymous substitution (*dS*), non-synonymous substitution (*dN*) and omega (*dN*/*dS* = ω) for every gene in every isolate using the yn00 program of PAML [15] implementing the Yang and Nielsen method [16] (Table S10). In total, we identified 1,450 genes with *dN*/*dS* ≥ 1 in at least one of our isolates, suggesting positive or diversifying selection. Of those genes, 283 were identified among *Bd*GPL isolates, 816 were identified among *Bd*CAPE isolates and 746 were identified among *Bd*CH isolates. Although no clear pattern could be distinguished within the *Bd*GPL (Fig. S17), CRN-like genes in both *Bd*CAPE and *Bd*CH had the greatest upper quartile and upper tail ω (Fig. S18). In addition, ω values for secreted chitin-associated genes and secreted proteases were marginally higher than their non-secreted counter parts. Uncharacterized secreted genes also had a greater ω than either of those non-secreted gene groups. Finally, a significant enrichment of CRN-like genes and uncharacterized (secreted) genes with ω≥1 were identified in both of these lineages (Table S11).

To further look for selection along the three lineages of *Bd*, we analysed each of those genes with the Branch site model (BSM) A (model=2, NSsites=2, fix_omega=0) compared with the null model (model=2, NSsites=2, fix_omega=1, omega=1) for each lineage (using the UPGMA tree from all verified homozygous loci in all isolates) implemented in the program codeml in PAML [15]. Next, we calculated 2 * the log likelihood difference between the two compared models (2D‘) with two degrees of freedom, and identified any with values greater than 8.1887 and 11.4076 (5% and 1% significance after Bonferroni correction).

A total of 482/1,450 (33%) of the genes with *dN*/*dS* ≥1 were found to have undergone positive selection in one of the lineages (2D’>8.1887, Table S10). Of those 482 genes, 349 were found among *Bd*GPL isolates, 109 among *Bd*CAPE isolates and 107 among *Bd*CH isolates. Nine genes were identified in all three lineages: four uncharacterised (secreted) with transcript ID's 05565, 02533, 00379, 06783 and five uncharacterised (non-secreted) with transcript ID's 03962, 07794, 05877, 02935, 08088 (Fig. S19). However, no clear pattern between gene categories and overlap could be seen (Table S11)

For *Bd*CAPE and *Bd*CH, a greater percent of each of the secreted gene categories were found to have undergone positive selection compared with their non-secreted counterpart gene categories. Specifically, 21% (28% for *Bd*CAPE and 14% for *Bd*CH) of the chitin-associated (secreted) with ω≥1 also had 2D'>8.1887, while only 14% (18% for *Bd*CAPE and 9% for *Bd*CH) of the chitin-associated (non-secreted) also had 2D'>8.1887.13% of the protease (secreted) genes with ω≥1 also had 2D'>8.1887, while only 6.9% of the protease (non-secreted) genes also had 2D'>8.1887. Finally, 13% uncharacterised (secreted) with ω≥1 also had 2D'>8.1887, while only 5.7% of uncharacterised (non-secreted) genes also had 2D'>8.1887. For *Bd*CAPE and *Bd*CH, a moderate enrichment for uncharacterized (secreted) were identified with 2D'>8.1887 from those genes with ω≥1, and a small enrichment for protease (secreted) in *Bd*CH (Table S11). Although no clear enrichment for genes with ω≥1 and 2D'>8.1887 was identified for *Bd*GPL, it is striking that 72% of the genes undergoing positive selection were identified on this lineage compared with only 23% for each of the other two lineages. This finding suggests that *Bd*GPL has been undergoing greater levels of positive selection than either *Bd*CAPE or *Bd*CH.

**References**

1. Li H, Durbin R (2009) Fast and accurate short read alignment with Burrows-Wheeler transform. *Bioinformatics* **25**:1754–60.

2. Li H, Handsaker B, Wysoker A, Fennell T, Ruan J, *et al.* (2009) The Sequence Alignment/Map format and SAMtools. *Bioinformatics* **25***:*2078-9.

3. Daum JM, Davis LR, Bigler L, Woodhams DC (2012) Hybrid advantage in skin peptide immune defenses of water frogs (*Pelophylax esculentus*) at risk from emerging pathogens. *Infect. Genet. Evol.* **12**:1854­1864.

4. Farrer RA, Henk DA, MacLean D, Studholme DJ, Fisher MC (2013) Using false discovery rates to benchmark SNP-callers in next-generation sequencing projects. *Sci. Rep.* **3**:1512 (scripts available at http://cfdr.sourceforge.net/).

5. Farrer RA, Weinert LA, Bielby J, Garner TWJ, Balloux F, *et al*. (2011) Multiple emergence of genetically diverse amphibian-infecting chytrids include a globalised hypervirulent lineage. *Proc. Natl. Acad. Sci. U. S. A.* **108**:18732-6.

6. Drummond AJ, Rambaut A (2007) BEAST: Bayesian evolutionary analysis by

sampling trees. *BMC Evol. Biol.* **7**:214.

7. Bendtsen JD, Nielsen H, Heijne GV, Brunak S (2004) Improved prediction of signal peptides: SignalP 3.0. *J. Mol. Biol.* **340**:783-795.

8. Rawlings ND, Barrett AJ, Bateman A (2010) MEROPS: the peptidase database. *Nucleic Acids Res.* **38**:D227–D233.

9. Malik A, Firoz A, Jha V, Ahmad S (2010) PROCARB: A Database of Known and Modelled Carbohydrate-Binding Protein Structures with Sequence-Based Prediction Tools. *Adv. Bioinformatics* 436036.

10. Götz S, García-Gómez JM, Terol J, Williams TD, Nagaraj SH, *et al.* (2008) High-throughput functional annotation and data mining with the Blast2GO suite. *Nucleic Acids Res.* **36**:3420–3435.

11. Dongen SV (2000) A cluster algorithm for graphs, Technical Report INS-R0010, National Research Institute for Mathematics and Computer Science in the Netherlands, Amsterdam.

12. McKenna A, Hanna M, Banks E, Sivachenko A, Cibulskis K, *et al.* (2010) The

Genome Analysis Toolkit: A MapReduce framework for analyzing next-generation DNA sequencing data. *Genome Res.* **20**:1297-1303.

13. Weir BS (1996) Genetic Data Analysis II. Sinauer, Sunderland.

14. Agapow P, Burt A (2001) Indices of multilocus linkage disequilibrium. *Mol.*

*Ecol. Notes* **1**:101–2.

15. Yang Z (2007) PAML 4: Phylogenetic Analysis by Maximum Likelihood. *Mol. Biol. Evol.* **24**:1586-91.

16. Yang Z, Nielsen R (2000) Estimating Synonymous and Nonsynonymous

Substitution Rates Under Realistic Evolutionary Models. *Mol. Biol. Evol.* **17**:32-43.
